# Recapitulating thyroid cancer histotypes through engineering embryonic stem cells

**DOI:** 10.1038/s41467-023-36922-1

**Published:** 2023-03-11

**Authors:** Veronica Veschi, Alice Turdo, Chiara Modica, Francesco Verona, Simone Di Franco, Miriam Gaggianesi, Elena Tirrò, Sebastiano Di Bella, Melania Lo Iacono, Vincenzo Davide Pantina, Gaetana Porcelli, Laura Rosa Mangiapane, Paola Bianca, Aroldo Rizzo, Elisabetta Sciacca, Irene Pillitteri, Veronica Vella, Antonino Belfiore, Maria Rita Bongiorno, Giuseppe Pistone, Lorenzo Memeo, Lorenzo Colarossi, Dario Giuffrida, Cristina Colarossi, Paolo Vigneri, Matilde Todaro, Giorgio Stassi

**Affiliations:** 1 Department of Surgical Oncological and Stomatological Sciences, University of Palermo, Palermo, Italy; 2 Department of Health Promotion Sciences, Internal Medicine and Medical Specialties, University of Palermo, Palermo, Italy; 3 Department of Clinical and Experimental Medicine, A.O.U. Policlinico-Vittorio Emanuele, Center of Experimental Oncology and Hematology, University of Catania, Catania, Italy; 4 Villa Sofia-Cervello Hospital, Palermo, Italy; 5 Queen Mary University, Experimental Medicine & Rheumatology, London, United Kingdom; 6 Endocrinology Unit, Department of Clinical and Experimental Medicine, University of Catania, Garibaldi-Nesima Hospital, Catania, Italy; 7 Department of Experimental Oncology, Mediterranean Institute of Oncology, Viagrande, Catania, Italy; 8 A.O.U.P. “Paolo Giaccone”, University of Palermo, Palermo, Italy

**Keywords:** Thyroid cancer, Cancer stem cells

## Abstract

Thyroid carcinoma (TC) is the most common malignancy of endocrine organs. The cell subpopulation in the lineage hierarchy that serves as cell of origin for the different TC histotypes is unknown. Human embryonic stem cells (hESCs) with appropriate in vitro stimulation undergo sequential differentiation into thyroid progenitor cells (TPCs-day 22), which maturate into thyrocytes (day 30). Here, we create follicular cell-derived TCs of all the different histotypes based on specific genomic alterations delivered by CRISPR-Cas9 in hESC-derived TPCs. Specifically, TPCs harboring *BRAF*^*V600E*^ or *NRAS*^*Q61R*^ mutations generate papillary or follicular TC, respectively, whereas addition of *TP53*^*R248Q*^ generate undifferentiated TCs. Of note, TCs arise by engineering TPCs, whereas mature thyrocytes have a very limited tumorigenic capacity. The same mutations result in teratocarcinomas when delivered in early differentiating hESCs. Tissue Inhibitor of Metalloproteinase 1 (TIMP1)/Matrix metallopeptidase 9 (MMP9)/Cluster of differentiation 44 (CD44) ternary complex, in cooperation with Kisspeptin receptor (KISS1R), is involved in TC initiation and progression. Increasing radioiodine uptake, KISS1R and TIMP1 targeting may represent a therapeutic adjuvant option for undifferentiated TCs.

## Abstract (web summary)

Thyroid carcinoma (TC) is the most common malignancy of endocrine organs. Here, the authors show the ability of human embryonic stem cells (hESCs) to recapitulate the different TC histotypes upon specific genomic alterations delivered by CRISPR-Cas9 and identify KISS1R and TIMP1 targeting as a therapeutic adjuvant option for undifferentiated TCs.

## Introduction

Follicular cell-derived TCs mostly develop through the aberrant growing of endodermal layer-derived follicular epithelial cells and comprise the main histological differentiated and undifferentiated subtypes^1,2^. Differentiated TCs include the most common papillary (PTC, 80-85%) and follicular (FTC, 10-15%) TCs. Although most differentiated TCs are indolent tumors with a favorable outcome, 5–20% of them have a high risk of relapse and death^3^. Anaplastic TC (ATC) represents 1-2% of all TC cases and behaves very aggressively by invading adjacent tissues and metastasizing distant organs^4^. ATC can be derived either from the follicular thyroid epithelium gland by losing the biological features, including the iodine uptake (de novo), or more frequently from a dedifferentiation process of a pre-existing differentiated TC, following the accumulation of multiple mutations^5^. Despite the intense efforts to improve therapeutic regimens, there are no effective therapies available for metastatic TC patients. The majority of TC metastases do not express the sodium/iodine symporter (NIS) and are not responsive to radioactive iodine, the most effective non-surgical therapy in TC^6–8^. Thus, there is an urgent need to develop experimental models to study the fundamental mechanisms underlying tumorigenesis and metastasis formation, particularly in radioiodine-resistant patients.

Despite major advances in the know-how of TC’s hallmarks and biological behavior, the cell subpopulation in the lineage hierarchy that serves as cell of origin for the different TC histotypes, following the acquisition of somatic mutations, remains unknown. A critical issue argues whether the different TC histotypes mirror a distinct transformed cell subpopulation or the same target cell harboring different genetic mutations. The existence of different follicular histotypes of cells-derived TCs endowed with distinctive biological behavior led to formulate several carcinogenesis models that describe the cell of origin^1,2^. However, these models do not reflect the phenotypic and genetic intertumoral heterogeneity of follicular cell-derived TCs. In adults mammals, tissue homeostasis and repair of injured organs depends on small reservoirs of tissue-specific stem cells able to self-renew and differentiate. Indeed, the hierarchical organization of adult tissue has been accomplished to slow aging, help the organism recover from injury, and finally protect cells from accumulating damage that would ultimately lead to cancer. Although the progenitor cells with limited lifespan escape the risk of accumulating mutations, adult stem cells and their immediate offsprings could potentially be transformed either by acquiring mutations inducing self- renewal, or by inheriting existing mutations from stem cells^9^. This model introduced the concept of tumorigenesis assuming stem cells as source of tumor formation in light of their longevity and innate self-renewal capacity, essential for the accumulation of genetic or epigenetic alterations^10,11^. To explain the different tumor histopathology and behavior, a genetic mutation model, in which a variety of genetic mutations occurs within the same “target cell”, was conceptualized^2^. For most cancers, the “target cell” of transforming mutations is unknown. Nevertheless, there are considerable evidence that certain types of leukemia arise from mutations which accumulate in hematopoietic progenitor cells^12–14^. This observation mostly reflects the need to better understand the oncogenic events which occur in tissue-resident stem cells in order to explain the transition from non-malignant hyper-proliferative lesions to well-established cancers endowed with different biological behaviors. Although we and other research groups have identified a subpopulation of cancer stem cells (CSCs)/progenitor cells that is crucially involved in the TC initiation, the transformation stage in the hierarchy lineage remains elusive^15^. Here, we show the ability of human embryonic stem cells (hESCs) to recapitulate the cellular hierarchy of thyroid gland and reproduce the oncogenic process upon CRISPR-based genetic editing.

## Results

### hESCs engineered with the most common TC genetic alterations recapitulate TC histotypes

**Fig. 1:**
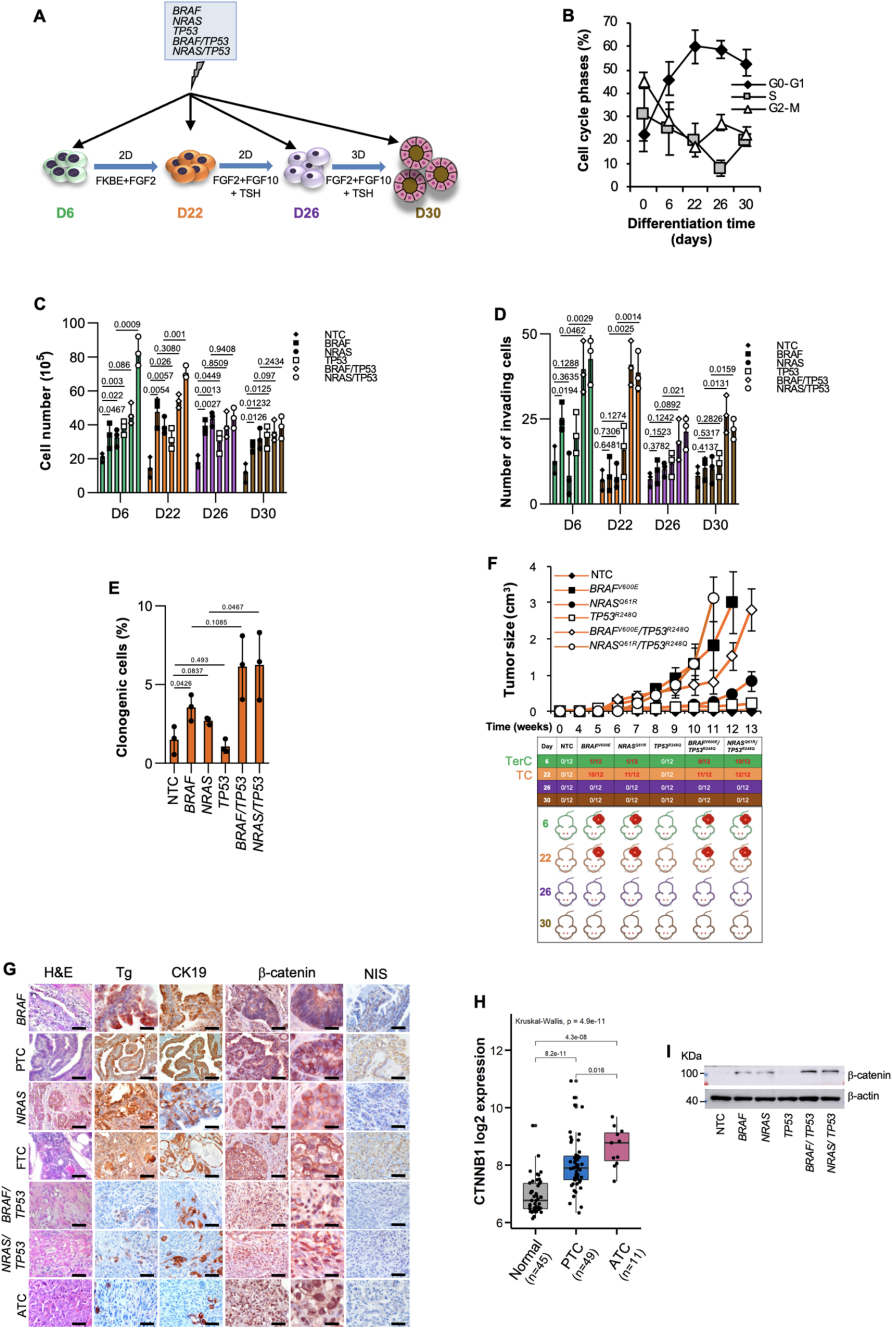
hESCs engineered with the most common TC genetic alterations recapitulate thyroid cancer histotypes. **A** Model of directed differentiation of human embryonic stem cell (hESC) into thyroid lineage. To promote thyroid lineage specification, hESCs were exposed to the indicated stimuli. Treatment with FGF10, KGF, BMP4 and EGF is reported as FKBE. **B** Cell cycle analysis in non-targeting control (NTC) hESC-derived cells at the indicated stage of thyroid differentiation lineage. The data show percentage of cell number in G0/G1, S, and G2/M cell cycle phases. Data are expressed as mean ± SD of three independent experiments. **C** Cell proliferation in hESC-derived cells, engineered with the indicated mutations, at the indicated stage of thyroid differentiation lineage, at 7 days. **D** Invasion analysis in cells engineered as in (**C**) at 72 h. **E** Clonogenic assay in D22 thyroid progenitor cells (TPCs) engineered as in (**C**) at 21 days. For (**C–E**) statistical significance was calculated using the two-tailed unpaired t test and data are mean ± standard error of three independent experiments. **F** (*upper panel*) Growth kinetics of xenograft tumors generated by subcutaneous injection of D22 TPCs. (*lower panel*) Frequency of teratocarcinoma (TerC) or TCs obtained by the injection of hESC-derived cells harboring different mutational background, at different stages of thyroid differentiation lineage. Data are shown as mean ± SD. *n* = 12 mice per group. **G** H&E staining and immunohistochemistry analysis of thyroglobulin (Tg), cytokeratin 19 (CK19), β-catenin and NIS on xenograft tumors obtained following injection of D22 TPCs engineered with different mutational background and compared with patient-derived PTC, FTC and ATC. Number of tissues analyzed *n* = 5. Mutational status of human tissues: PTC *BRAF* mutated ID#6, FTC *NRAS* mutated, and ATC *BRAF/TP53* mutated ID#96. Scale bars, 100 µm. **H** Correlation analysis in Gene Expression Omnibus (GEO) (GSE33630) of *CTNNB1* mRNA expression levels in normal thyroid tissue, PTC and ATC. Boxes represent the interquartile range (IQR) and midline represents the median. Statistical significance was calculated using Kruskal-Wallis test. **i** Immunoblot analysis of β-catenin in D22 TPCs engineered as in (**c**). β-actin was used as loading control. Source data are provided as a Source Data file.

To establish a model that mirrors the follicular cell-derived TC histotypes, hESCs-derived thyroid progenitor cells (TPCs) and their differentiation lineages were obtained following Longmire’s established protocol^16^ and genetically modified using a CRISPR/Cas9 technology by introducing the most common TC genetic alterations including *BRAF*, *NRAS* and *TP53* (Fig. 1A and Supplementary Fig. 1a). According to literature, cells at day 6, upon activation of BMP-FGF2 signaling, promoted thyroid lineage specification gradually increasing, up to day 30, the expression levels of thyroid markers PAX8, TTF1, TSH-R, TPO, Tg and NIS (Supplementary Figs. 1b–e)^16–18^. D22 cells triggered the expression of specific thyroid lineage markers, PAX8 and TTF1, which are retained and concomitantly complemented, at D26 and D30, by the activation of thyroid differentiation markers, TSH-R, TPO, Tg, and NIS (Supplementary Figs. 1c–e). Besides, D22 cells expressed high levels of other putative thyroid progenitor cell hallmarks such as CD133, ABCG2, Nestin and HNF-4α^19^, along with the thyroid transcription factors *HHEX* and *FOXE1*^20^ (Supplementary Figs. 1f–j). All together these data indicate that D22 thyroid lineage is enriched in thyroid progenitor cells (TPCs). Mature lung markers *SCGB1A1*, *AQP5*, *SFTPB* and *SFTPC* were barely detectable in the thyroid lineage from days 22 to 30 (Supplementary Fig. 1k). While throughout hESC differentiation, cells at days 0, 6 and 22 were progressively enriched in G0-G1 and depleted in G2-M/S phase fractions, most of the differentiated thyroid precursors at days 26 and 30 displayed a slight decrease of G0-G1 and increase of G2-M phases (Fig. 1B).

D22 TPCs showed a similar cell cycle profile upon *BRAF*, *NRAS* and *TP53* mutational background introduction. Sanger and Next-generation sequencing analyses exhibited a high-efficiency percentage of the introduced exogeneous genetic mutations that was consistent with a correlation coefficient of 0.85% (Supplementary Figs. 2a–c and Supplementary Table 1). hESC-derived anterior foregut endoderm at day 6 and D22 TPCs, harboring *TP53*^*R248Q*^ in combination with *BRAF*^*V600E*^ or *NRAS*^*Q61R*^, showed more pronounced proliferative, invasive capabilities and clonogenic activity, compared to NTC control (Fig. 1C–E). Upon the acquisition of specific genetic alterations, except the inactivating point *TP53*^*R248Q*^ mutation alone, exclusively cells at days 6 and 22 retained tumorigenic capacity (Fig. 1F). Subcutaneous injection of the genetically engineered D22 TPCs generated avatars that recapitulate the recognizable phenotypic and morphological features of follicular cell-derived TC histotypes, whereas D6 cells-derived xenograft masses resembled a teratocarcinoma phenotype expressing S-100, CDX2, Oct3/4 and p40 (Fig. 1G and Supplementary Fig. 2d).

Specifically, *BRAF*^*V600E*^ and *NRAS*^*Q61R*^-engineered D22 cells led to the formation of tumors that reflect the cellular heterogeneity of patient-derived PTC and FTC phenotype, respectively, mainly characterized by different degrees of nuclear atypia and variable mitotic activity (Fig. 1G).

**Fig. 2:**
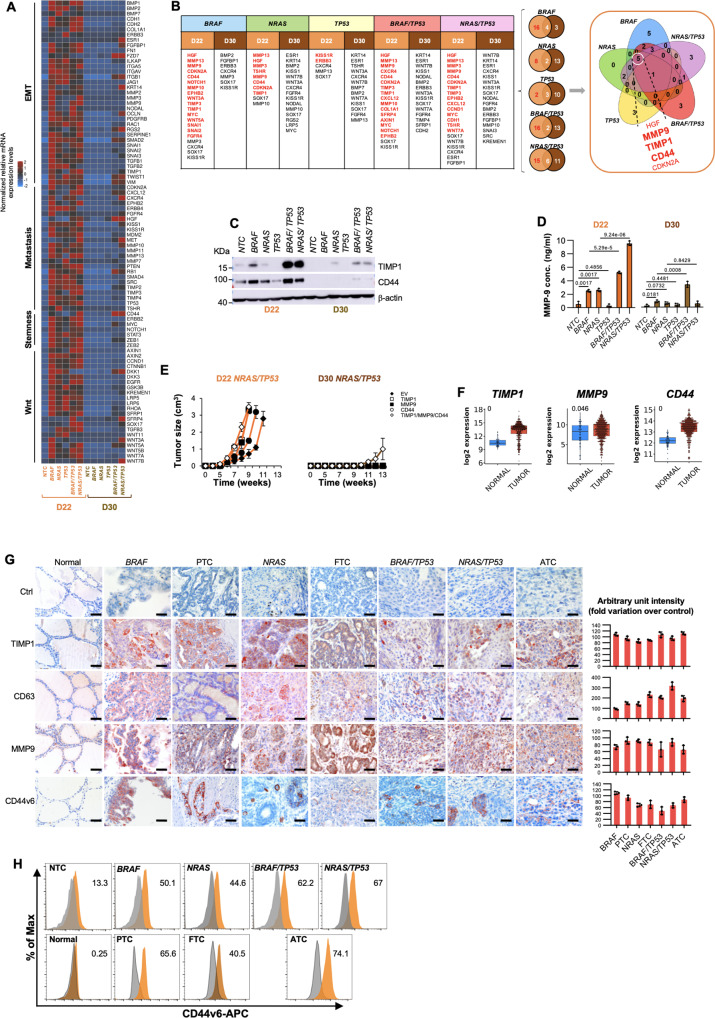
The ternary complex TIMP1-MMP9-CD44 sustains the tumor initiation capability of engineered D22 TPCs. **A** Heatmap of Wnt-, stemness-, metastasis- and EMT- related genes in D22 TPCs and in D30 mature thyrocytes engineered with the indicated mutations. Data are presented as 2^−ΔCt^ normalized values of three independent experiments. **B** Table and Venn diagrams of common (black color) and exclusive upregulated genes (red color) with logFC > 1.5 in D22 TPCs vs thyrocytes (D30). **C** Immunoblot analysis of TIMP1 and CD44 in hESC-derived cells harboring different mutational background, at D22 and D30. β-actin was used as loading control. One representative of three independent experiments is shown. Source data are provided as a Source Data file. **D** MMP9 production in cells as in (**C**) at 48 h. Statistical significance was calculated using the unpaired two-tailed t test. Data are expressed as mean ± SD of three independent experiments. **E** Growth kinetics of xenograft tumors generated by subcutaneous injection of the indicated engineered D22 TPCs and D30 cells overexpressing *TIMP1*, *MMP9* and *CD44* alone and in combination. Data are shown as mean ± SD. *n* = 10 mice per group. **F** Relative mRNA expression levels of *TIMP1, MMP9* and *CD44* in normal (*n* = 59) and tumor (*n* = 509) thyroid tissue from TGCA database, thyroid carcinoma (THCA) branch. Boxes represent the IQR and midline represents the median. Statistical significance was calculated using the Wilcoxon test. **G** (*left panel*) Immunohistochemical analysis of TIMP1, CD63, MMP9 and CD44v6 on xenografts obtained by injecting engineered D22 TPCs and compared with normal thyroid tissue, patient-derived PTC, FTC and ATC. Ctrl= negative control. The number of tissues analyzed *n* = 5. Mutational status of human tissues: PTC *BRAF* mutated ID#6, FTC *NRAS* mutated, and ATC *BRAF/TP53* mutated ID#96. Scale bars, 100 µm. (*right panel*) Histograms represent the percentage of TIMP1, CD63, MMP9 and CD44v6 positive cells. Data are expressed as mean ± SD of three independent experiments. **H** CD44v6 flow cytometry analysis (orange histograms) and corresponding isotype-matched control (grey histograms), in engineered D22 TPCs and compared with isolated normal thyroid cells and patient-derived PTC, FTC and ATC cells.

Although the inactivation of p53 alone was not required for the tumor initiation, D22 TPCs harboring *TP53*^*R248Q*^ in combination with *BRAF*^*V600E*^ or *NRAS*^*Q61R*^ generated undifferentiated tumors with a pleomorphic and undifferentiated morphology recapitulating the heterogeneity and the phenotypic characteristics of ATC, expressing low levels of Tg, CK19 and NIS as compared with the well-differentiated TC histotypes (Fig. 1F, G and Supplementary Fig. 2e). In line with current knowledge, these data suggest that *TP53* mutations are required for tumor evolution and a higher disease stage exclusively in cooperation with other oncogenic hits^21^. Accordingly, *TP53* mutation was more frequently positively associated with undifferentiated tumors showing aggressive features and distant metastases^22–24^ (Supplementary Fig. 2f). This phenomenon is in line with the analysis of *TP53* germline mutation distribution in a large cohort of patients affected by Li-Fraumeni syndrome, which highlighted a rare positive association with inherited risk of developing TCs (Supplementary Fig. 2g). Moreover, in accordance with the positive association between *CTNNB1* mRNA levels and aggressive histotypes of TC^25^, D22 TPCs engineered with *TP53*^*R248Q*^ in combination with *BRAF*^*V600E*^ or *NRAS*^*Q61R*^ mutations displayed β-catenin accumulation and nuclear localization (Fig. 1G–I and Supplementary Fig. 2h). In D22 cells, *BRAF*^*V600E*^ and *NRAS*^*Q61R*^ alone or in combination with *TP53*^*R248Q*^ mutation promoted the expression of CSC-, EMT-, and metastasis-related genes (Fig. 2A). These findings suggest that thyroid carcinogenesis follows a genetic mutation model in which the TPCs are the elective target of genetic alterations.

### TIMP1-MMP9-CD44 complex sustains tumor initiation capability of engineered D22 TPCs

To explore the signaling pathway implicated in the tumorigenesis of engineered TPCs, we sought to analyze the reprogramming of the transcriptomic profile sustained by specific oncogenic alterations.

From transcriptomic analysis of engineered D22 TPCs with *BRAF*^*V600E*^ or *NRAS*^*Q61R*^ alone and in combination with *TP53*^*R248Q*^ mutation emerged a common higher expression of *HGF*, *TIMP1*, *MMP9* and *CD44* compared with D30 mature thyroid cells (Fig. 2A, B and Supplementary Fig. 3a). As the previously reported activation of HGF/MET signaling pathway in ATCs^26^, HGF was found among the top differentially upregulated genes in D22 compared with D30 (Fig. 2A, B).

These findings are in line with TIMP1 and CD44 protein expression patterns and MMP9 activity in genetically edited cells at days 22 and 30 stage of thyroid differentiation lineage (Fig. 2C, D, and Supplementary Fig. 3b). mRNA and protein analysis of hESCs at different stages of differentiation revealed that expression levels of TIMP1, MMP9 and CD44, in a time-dependent manner, increase from D0 to D22, and then decrease to D30 (Supplementary Figs. 3C–E). In accordance with the lack of tumorigenic activity, *TP53*^*R248Q*^ mutation alone did not endorse the expression of these molecules (Fig. 2A–D and Supplementary Figs. 3a, b). Although TIMP1 is a natural inhibitor of MMPs involved in the matrix remodeling during carcinogenesis, it converges toward cancer cell survival by binding MMP9/CD44 complex and mediating PI3K/AKT pathway activation^27,28^. TIMP1, together with its binding protein CD63, has emerged as a predictive biomarker with regard to tumor therapeutic response and spreading^29^. Differentiated cells at days 26 and 30, characterized by negligible expression levels of TIMP1, MMP9 and CD44, were unable to induce tumor growth (Supplementary Figs. 3f, g). Interestingly, the overexpression of TIMP1 together with CD44 and MMP9, is able to significantly increase the proliferation rate and in lesser extent the invasive capacity of D30 cells harboring *NRAS*^*Q61R*^*/TP53*^*R248Q*^ mutations, whose cells have acquired a weak tumorigenic activity, tardily generating small tumors. This phenomenon could be attributed to the concurrent epigenetic alterations, induced by the ternary complex, along the differentiation process (Fig. 2E and Supplementary Figs. 4a–d).

In the light of these data, we investigated whether the expression of each member of the ternary complex TIMP1-MMP9-CD44 correlated with thyroid tumor. Analysis of a large cohort of primary TCs showed a significant positive association with the expression of *TIMP1*, *MMP9* and *CD44* (Fig. 2F and Supplementary Fig. 4e)^30^. Following the ternary complex formation, TIMP1 leads to the activation of MMP9, which in turn cleaves the CD44 extra-cellular domain hence releasing the intra-cellular fragment into the cytoplasm^31^. The CD44 fragment translocates into the nucleus, where it acts as a transcription factor, by regulating the expression of several target genes including the variant isoforms of CD44 (CD44v)^32^.

Some of CD44v have been considered as functional markers of cancer progression^33^. We have recently reported that constitutive and reprogrammed metastatic cancer cells are identifiable by the expression of CD44v6, which is positively associated with PI3K/AKT pathway activation^34^.

**Fig. 3:**
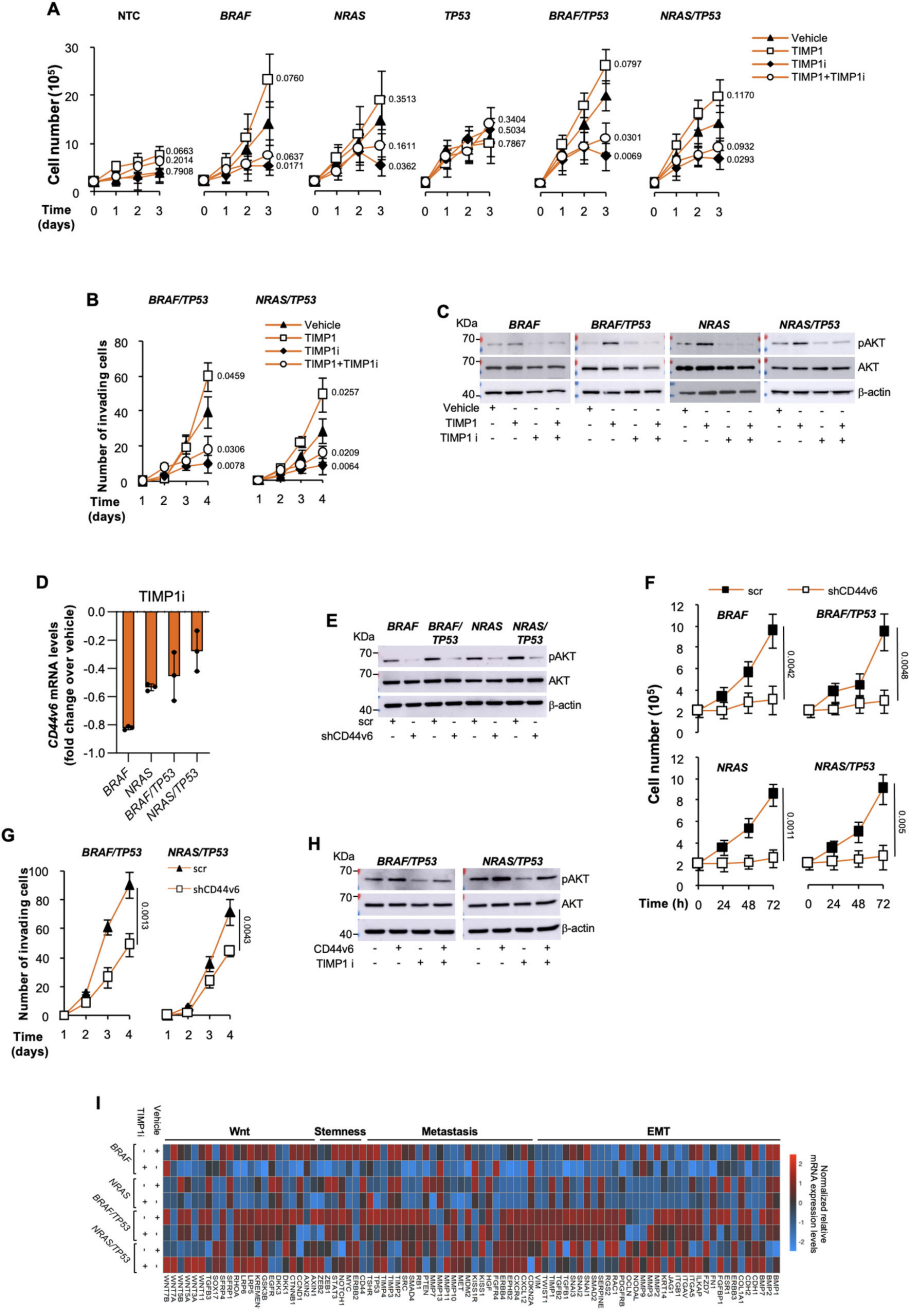
TIMP1 blockade and CD44v6 silencing significantly impair survival and invasive capacity of D22 TPCs. **A** Cell proliferation in D22 TPCs engineered with the indicated mutations treated with vehicle, TIMP1, TIMP1 inhibitor (TIMP1i) alone or in combination up to 3 days. **B** Invasion analysis in D22 TPCs harboring the indicated mutations treated as in (**A**) up to 4 days. For (**A** and **B**) statistical significance was calculated using the two-tailed unpaired t test and data are mean ± standard error of three independent experiments. **C** Immunoblot of pAKT and AKT in engineered D22 TPCs treated as in (**A**) for 48 h. β-actin was used as loading control. One representative of three independent experiments is shown. Source data are provided as a Source Data file. **D** Relative mRNA expression levels of *CD44v6* in D22 TPCs engineered with the indicated mutations and treated with TIMP1 inhibitor (TIMP1i) for 24 h. Data are presented as fold change over vehicle ± SD of three independent experiments. **E** Immunoblot analysis of pAKT and AKT levels in D22 TPCs engineered with the indicated mutations and transduced with control shRNA (scramble, scr) or CD44v6 shRNA (shCD44v6). β-actin was used as loading control. One representative of three independent experiments is shown. Source data are provided as a Source Data file. **f** Cell proliferation in D22 TPCs engineered with the indicated mutations transduced with control shRNA (scramble, scr) or CD44v6 shRNA (shCD44v6), up to 72 h. **G** Invasion analysis in D22 TPCs harboring the indicated mutations transduced with control scramble (scr) or shCD44v6, up to 4 days. For (**F** and **G**) statistical significance was calculated using the two-tailed unpaired t test and data are mean ± standard error of three independent experiments. **H** Immunoblot analysis of pAKT and AKT in the indicated engineered D22 TPCs overexpressing CD44v6, untreated and treated with TIMP1i for 48 h. β-actin was used as loading control. One representative of three independent experiments is shown. Source data are provided as a Source Data file. **I** Heatmap of Wnt-, EMT- stemness and metastasis-related genes in D22 TPCs engineered with the indicated mutations and treated with vehicle or TIMP1i for 24 h. Data are presented as normalized 2^−ΔCt^ values of three independent experiments.

We next evaluated whether TIMP1, interacting with CD63, is required for cell survival and migration, cooperating with CD44v6. D22 cell-derived xenograft tumors, harboring *BRAF*^*V600E*^ and *NRAS*^*Q61R*^ alone or in combination with *TP53*^*R248Q*^ mutation expressed comparable levels of TIMP1, CD63, MMP9 and CD44v6 with those found in the matching PTC, FTC and ATC histotypes (Fig. 2G, H, and Supplementary Figs. 4f, g). In accordance with the inability to promote tumor initiation, *TP53*^*R248Q*^ mutation alone lacked the capability to induce CD44v6 expression in D22 TPCs (Supplementary Fig. 4h). TIMP1 targeting significantly impaired the proliferative and invasive capacity of the engineered D22 TPCs, regardless the introduced genetic alteration, by impeding the activation of PI3K/AKT pathway, even in presence of TIMP1 exogenous cytokine, driven by a reduced expression level of CD44v6^34^ (Fig. 3A–D, and Supplementary Figs. 5a–d).

Transduction of engineered D22 TPCs with a lentiviral vector encoding for *CD44v6* short hairpin RNA (shRNA) sequences, significantly decreased AKT phosphorylation, proliferative and invasive potential (Fig. 3E–G and Supplementary Fig. 5e–g). Conversely, CD44v6 overexpression led to an upregulation of pAKT, whose protein expression levels were restored by the targeting of TIMP1, to lower levels expressed by untreated TPCs (Fig. 3H and Supplementary Figs. 5h–j). These findings suggest that TIMP1 through CD44v6 is required for the activation of PI3K/AKT pathway.

**Fig. 4:**
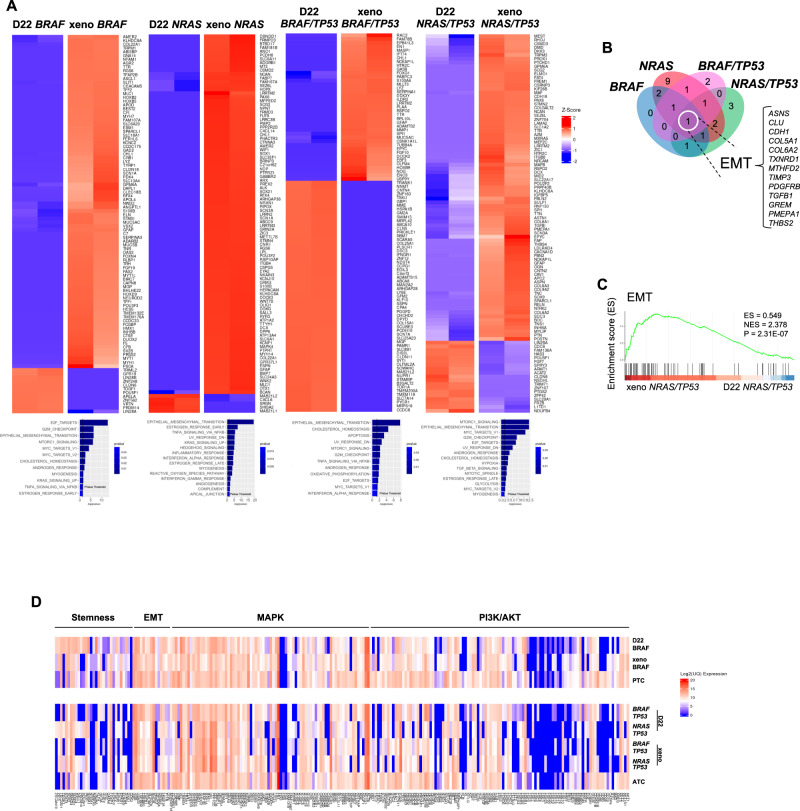
MAPK and PI3K/AKT pathways, along with EMT-associated signature, are enriched in D22 TPCs, mouse avatars and human PTC and ATC. **A** *(upper panels)* Heatmaps showing the top 100 differentially expressed genes (DEGs) with logFC > 2 obtained by total transcriptome analysis (mRNAseq), in engineered D22 TPCs harboring the pathogenetic TC mutations versus their derived-xenograft tumors. *(lower panels)* Barplots indicate the significantly enriched pathways (*p* value < 0.05) performed using a Gene set enrichment analysis (GSEA) on Hallmark category from MSigDB database of differentially expressed genes (DEGs) obtained by RNAseq. **B** Venn diagram of the common enriched pathways in xenograft tumors versus D22 TPCs. List of the common 13 differentially regulated genes, associated with EMT-pathway. **C** Gene set enrichment analysis (GSEA) of EMT pathway in xeno *NRAS/TP53* versus *D22 NRAS/TP53* (ES = 0.549, NES = 2.378, *p* = 2.31E-07). Statistical significance was calculated using Benjamini & Hochberg test. **D** Heatmap showing normalized mRNA expression values of genes involved in PI3K/AKT-, MAPK-, EMT- and stemness-related pathways in the indicated engineered D22 TPCs, D22-derived xenograft tumors and human PTC and ATC. Mutational status of human tissues: PTC *BRAF* mutated ID#6, and ATC *BRAF/TP53* mutated ID#96. Values are expressed as Log2 upper quartile (UQ).

Likewise, in the TPCs harboring the different mutation profiles, TIMP1 blockade downregulated the expression of Wnt, EMT- and stemness-related genes, as also confirmed by enrichment analysis computed with Reactome Database (Fig. 3I and Supplementary Figs. 6a–d). Specifically, targeting of TIMP1 hampered the mRNA levels of ß-catenin targets, such as *AXIN2*, *MYC* and *CCND1* and the cell growth rate, which were both restored in presence of Wnt pathway agonists, Wnt3A and R-spondin1 (Supplementary Figs. 6e–h). In line with these findings, engineered D22 TPCs and their derived mouse avatars, showed similar transcriptomic profiles and an enrichment of pathways related to EMT- and stemness-associated signature, as well as PI3K/AKT and MAPK signaling, comparable to that observed in primary PTC- and ATC tissues (Fig. 4A–D and Supplementary Figs. 7a, b). Altogether, these data indicate that specific mutations regulate the expression of TIMP1/CD44v6 complex, which sustain the tumor growth of the different TC histotypes through the activation of PI3K/AKT pathway.

### KISS1R is a prognostic factor and a potential therapeutic target in advanced TCs

Given that, *BRAF*^*V600E*^ or *NRAS*^*Q61R*^ in combination with *TP53*^*R248Q*^ mutations gave rise to tumors that resemble the ATC phenotype, we evaluated whether these genetic alterations are required for the metastatic potential of TPCs. Cells were orthotopically injected and their xenograft-derived lesions analyzed for the gene expression related to the metastatic signaling profile.

**Fig. 5:**
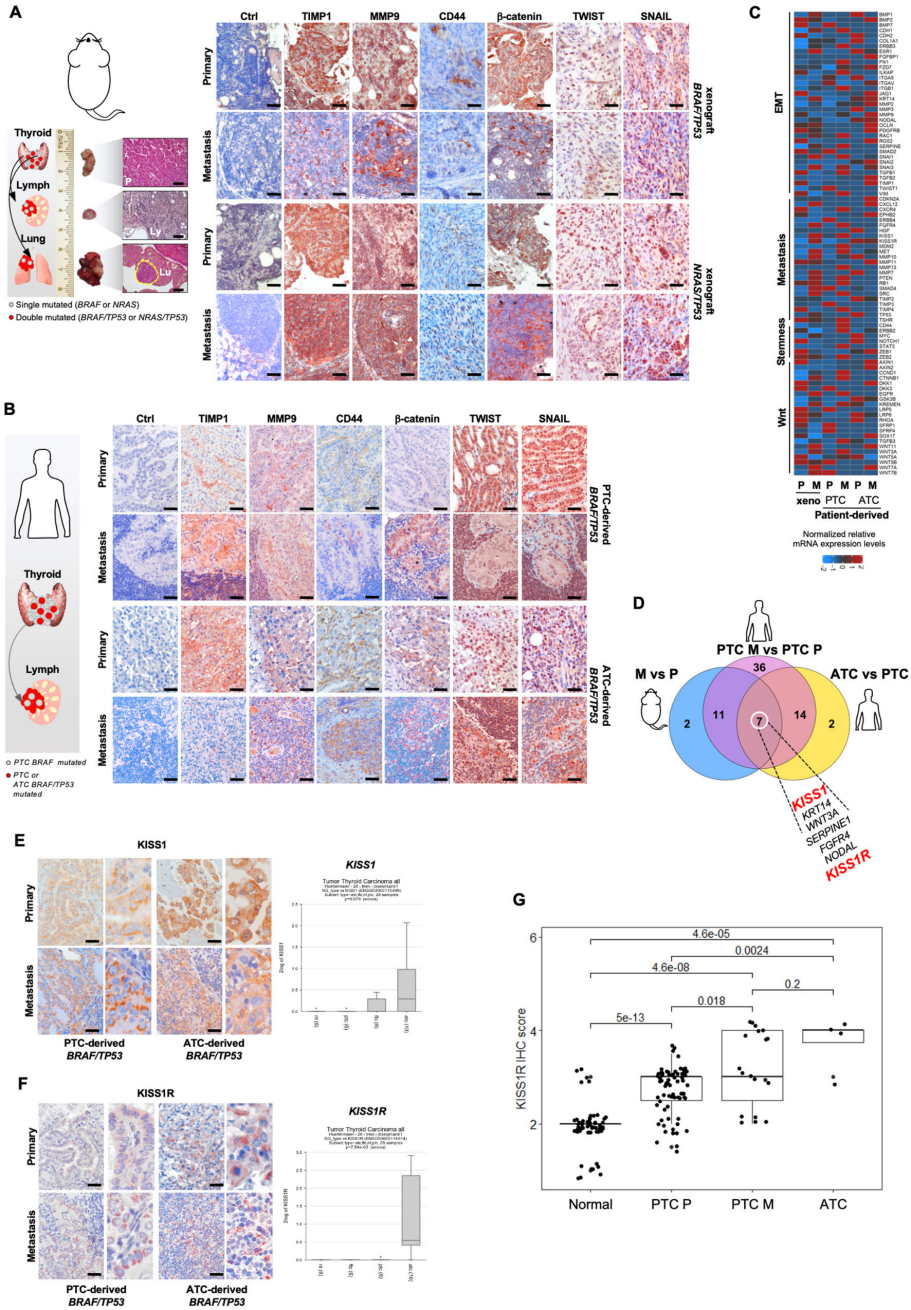
KISS1R is a prognostic factor and a potential therapeutic target in advanced TCs. **A** (*left panel*) H&E analysis of primary tumor (P), lymphnodes (Ly) and lung (Lu) metastatic lesions, generated by orthotopic injection of D22 TPCs engineered with the indicated mutations. Scale bars, 100 µm. (*right panel*) Immunohistochemical analysis of TIMP1, MMP9, CD44, β-catenin, TWIST and SNAIL on primary and metastasis mouse avatars. Ctrl= negative control. Scale bars, 100 µm. **B** Immunohistochemical analysis of TIMP1, MMP9, CD44, β-catenin, TWIST and SNAIL on primary and metastatic PTC (ID#61) and ATC (ID#96) patient-derived tumors harboring *BRAF/TP53* mutations. Ctrl= negative control. Scale bars, 100 µm. For (**A** and **B**) *n* = 5 tissues analyzed. **c** Heatmap of Wnt-, EMT- stemness and metastasis-related genes (2^−ΔCt^ expression values) in primary (P), metastatic (M) tumor xenografts and primary (P) and metastatic (M) PTC- and ATC-patient derived tumors, harboring *BRAF/TP53* mutations. Data are presented as normalized mRNA expression values of three independent experiments. **D** Venn diagram showing common upregulated genes with logFC > 3.5 in metastasis (M) *versus* primary (P) tumor xenografts, in PTC-derived metastasis (PTC M) *versus* primary tumors (PTC P) and in ATC primary *versus* PTC primary tumors. **E** and **F**, (*left panels*) Immunohistochemical analysis of KISS1 (**E**) and KISS1R (**F**) on primary and metastatic PTC (ID#61) and ATC (ID#96) patient-derived tumors harboring *BRAF/TP53* mutations. *n* = 5 tissues analyzed. Scale bars, 100 µm. (*right panels*) R2 database analysis of *KISS1* (**E**) and *KISS1R* (**F**) mRNA expression levels in normal thyroid (nt, *n* = 6), PTC (*n* = 6), FTC (*n* = 6) and ATC (*n* = 10) patient-derived tissues (R2 database Tumor Thyroid Carcinoma all - Huettelmaier − 28 - tmm - jbseqrnanb1). Boxes represent the IQR and midline represents the median. Statistical significance was calculated using ANOVA test. **G** Immunohistochemistry score analysis of KISS1R expression in normal thyroid tissue (*n* = 56), primary PTC tumors (PTC P, *n* = 73), PTC-derived loco-regional lymphnode metastasis (PTC M, *n* = 19) and primary ATC tumors (*n* = 5). Mutational status of 97 primary and metastatic human tissues is reported in Supplementary Data 2. Boxes represent the IQR and midline represents the median. Statistical significance was calculated using Kruskal-Wallis test.

TPCs engineered for *BRAF*^*V600E*^/*TP53*^*R248Q*^ and *NRAS*^*Q61R*^/*TP53*^*R248Q*^, robustly generated orthotopic and metastatic tumors (Fig. 5A). Immunohistochemical analysis showed that TIMP1/MMP9/CD44 ternary complex expression in primary tumor avatars paralleled an increase of the expression levels of TWIST, SNAIL in the xenograft metastasis, resembling the phenotypic landscape of PTC and ATC patient-derived primary and metastatic lesions (Fig. 5A, B).

From the transcriptomic analysis emerged a high expression of *KISS1* and *KISS1R* in TC metastatic lesions and in xenografts derived from TPCs harboring the mutation of *BRAF* or *NRAS* in combination with *TP53*, likely induced by the functional inactivation of p53, along with the activation of Wnt signaling and EMT-related pathways (Fig. 5C, D and Supplementary Figs. 8a–d). Although KISS1 is known as a metastasis suppressor gene, recent evidence highlighted its role in triggering the molecular events of the metastatic cascade^35–38^. Consistently, by binding its cognate receptor, KISS1 promotes invasion, intravasation and secondary lesion engraftments^37,39^.

Immunohistochemistry and transcriptomic analysis of a cohort of TC patients (Supplementary Table 2 and Supplementary Fig. 8e) revealed that KISS1 and KISS1R expression levels are positively associated with the aggressive phenotype including PTC-derived metastatic lesions and ATCs, in line with a RNAseq and DFS probability analysis of a large cohort of thyroid carcinoma, showing a significant correlation between high levels of KISS1R and the risk of developing TC metastatic disease (Fig. 5E–G and Supplementary Figs. 8f–i).

**Fig. 6:**
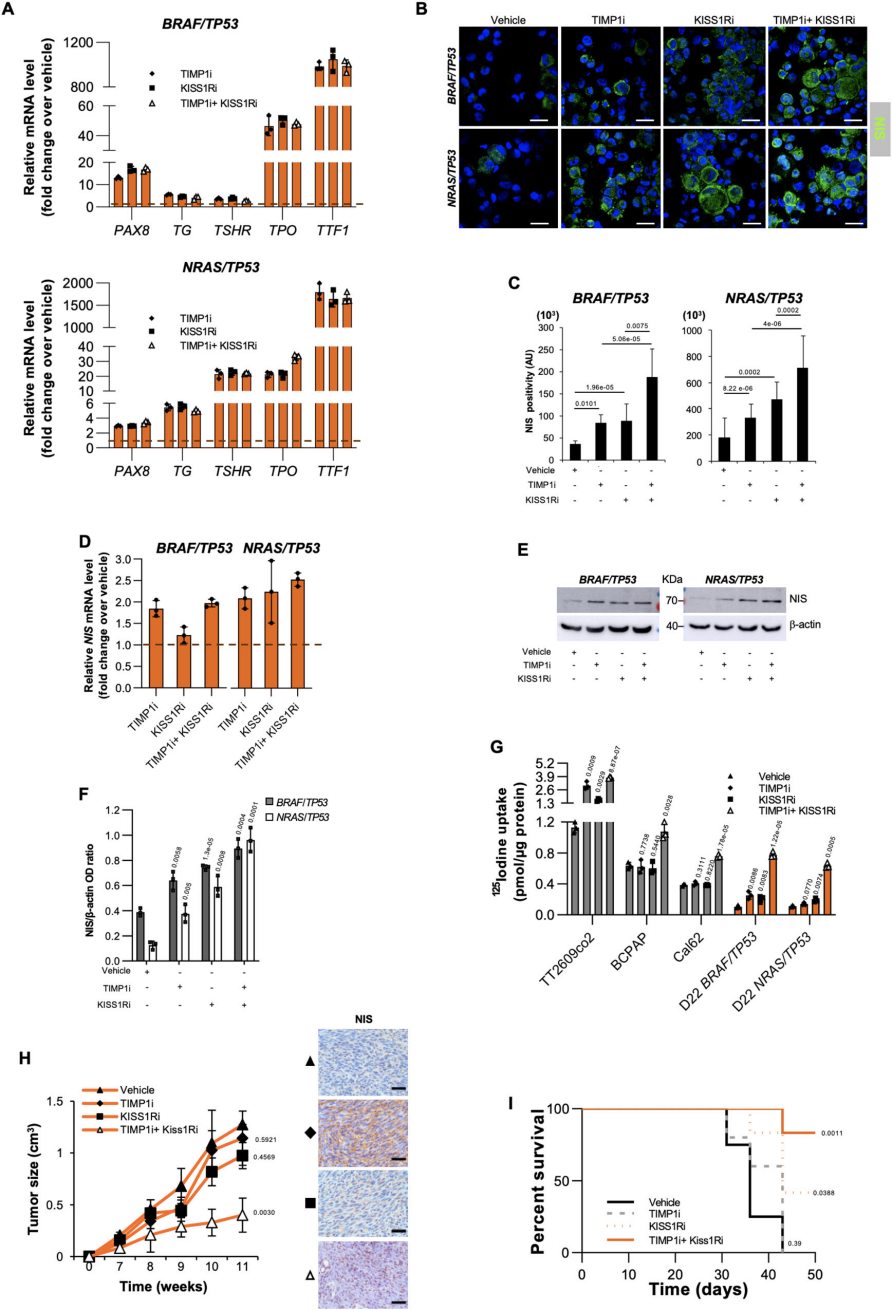
KISS1R and TIMP1 targeting restore the functional iodine uptake by increasing NIS expression. **A** Relative mRNA expression levels of *PAX8, TG, TSHR, TPO* and *TTF1* in the indicated engineered D22 treated with KISS1R inhibitor (KISS1Ri) alone or in combination with TIMP1 inhibitor (TIMP1i) for 24 h. Data are presented as fold change over vehicle ± SD of three independent experiments. **B** and **c** Immunofluorescence analysis of NIS and cell positivity in D22 TPCs engineered with the indicated mutations and treated as in (**A**) for 72 h. Nuclei were counterstained by Toto-3. Statistical significance was calculated using the unpaired two-tailed t test. Scale bars, 20 µm. Data are mean ± SD of 3 independent experiments. *n* = 12 wells analyzed. **D** Relative mRNA expression levels of *NIS* in cells engineered and treated as in (**A**) for 24 h. Data are presented as fold change over vehicle ± SD of three independent experiments. **E** and **F** Immunoblot of NIS (**E**) and relative optical density ratio (**F**) in cells as in (**A**) for 48 h. β-actin was used as loading control. One representative of three independent experiments is shown. Source data are provided as a Source Data file. **G** Radioiodine uptake in the indicated established thyroid cancer cell lines and engineered D22 TPCs treated as in (**A**) for 48 h. For (**F** and **G**) statistical significance was calculated using the two-tailed unpaired t test and data are mean ± standard error of three independent experiments. **H** (*left panel*) Growth kinetics of xenograft tumors generated by subcutaneous injection of D22 TPCs engineered for *NRAS*^*Q61R*^*/TP53*^*R248Q*^ treated with TIMP1 inhibitor (TIMP1i) and KISS1R inhibitor (KISS1Ri) alone and in combination. Statistical significance was calculated using the unpaired two-tailed t test. Data are shown as mean ± SD. *n* = 6 mice per group. (*right panels*) Immunohistochemical analysis of NIS on xenograft tumors obtained following injection of D22 TPCs engineered for *NRAS*^*Q61R*^*/TP53*^*R248Q*^ treated as indicated, *left panel*. Scale bars, 100 µm. **I** Kaplan-Meier graph showing the murine survival of D22 TPCs engineered with *NRAS*^*Q61R*^*/TP53*^*R248Q*^ treated as in (**H**). The statistical significance between groups was evaluated using a log rank Mantel-Cox test.

KISS1R targeting reduced both proliferative and invasive activity and also inhibited the activation of the PI3K/AKT and ERK pathways (Supplementary Figs. 9a–f). Indeed, several clinical trials have been designed to target concomitantly PI3K/AKT and MAPK pathway (NCT01830504). Although surgical resection in combination with radioactive iodine and l-thyroxine treatment is known to be effective in differentiated TCs^3^, patients who developed distant metastases or affected by ATCs are refractory to radioactive iodine^7,8^. Indeed, ATC and metastatic disease are still incurable due to their systemic nature and resistance to the currently accessible therapeutic approaches. In accordance with targeting of molecules regulating NIS trafficking, tyrosine kinases or HDAC and overexpression of *PAX8* gene^40–43^, we intriguingly observed that inhibition of KISS1R together with TIMP1 induced the expression of thyroid differentiation markers such as *PAX8*, *TG, TSHR*, *TPO*, *TTF1* and NIS, which sustained the increase of functional radioiodine uptake in *BRAF/TP53* and *NRAS/TP53* mutant D22 TPCs, whose ^125^Iodine retention levels were similar to those of untreated PTC established cell lines (Fig. 6A–G and Supplementary Fig. 9g). In xenograft thyroid tumors derived from the subcutaneous injection of mutated D22 TPCs, the intra-tumoral administration of TIMP1 and KISS1R inhibitors^44–46^ lead to a significant delay in tumor growth, characterized by a marked expression of NIS, and a prolonged percentage of mice survival (Fig. 6H, I).

Thus, these data indicate KISS1R and TIMP1 blockade as adjuvant therapy to sensitize undifferentiated TCs to radioiodine-based therapeutic regimen, reprogramming thyroid cancer cells behaving aggressively toward a more differentiated phenotype.

**Fig. 7:**
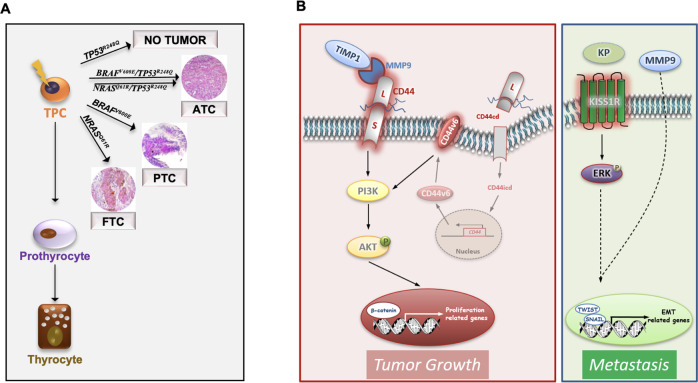
Model of TC tumorigenesis and progression. **A** Schematic model illustrating that the most common TC genetic alterations (*BRAF*^*V600E*^, *NRAS*^*Q61R*^, *BRAF*^*V600E*^*/TP53*^*R248Q*^ and *NRAS*^*Q61R*^*/TP53*^*R248Q*^) in thyroid progenitor cell (TPC) recapitulate the different TC histotypes (FTC, PTC and ATC). Of note *TP53*^*R248Q*^ alone is not required for TC initiation. **B** Model of ternary complex (TIMP1/MMP9/CD44) and KISS1R driven pathways in engineered D22 TPCs. TIMP1 and pro-MMP9 complex formation activates MMP9 and consequently leads to the cleavage of CD44. The CD44 intracytoplasmic domain (CD44icd) translocates into the nucleus where it induces *CD44v6* transcription. CD44v6 promotes TPC proliferation through PI3K/AKT pathway. The binding of kisspeptins (KP) to KISS1R activates ERK and cooperates with MMP9 to promote the transcription of EMT-related genes, including *TWIST* and *SNAIL*, driving metastatic engraftment.

Our findings suggest that TC histopathology and behavior are determined by distinct genetic alterations and that while the ternary complex is crucial for the promotion of TC, KISS1R sustains the metastatic growth (Fig. 7A). Triggering of KISS1R leads to the acquisition of an invasive phenotype through the activation of both PI3K and ERK-mediated MMP9 signaling pathway^47^, which positively regulated the expression of EMT-associated genes (Fig. 7B and Supplementary Fig. 9h).

## Discussion

In this study, we reproduced the oncogenic events that occur in tissue-resident stem cells/progenitor cells during TC transformation. We demonstrated that genetic mutations occurring in tissue-resident progenitor cells, characterized by a long-lasting cell lifespan, govern the evolutionary dynamics of thyroid tumorigenesis. Thus, this phenomenon could explain the TC incidence rates dictated by the rare (1/1000) presence of stem/progenitor cells in the thyroid gland^48^, which is renewed few times during the whole life^49^. We recapitulated all the different TC histotypes based on the induction of specific genomic alteration delivered by CRISPR-Cas9 in human ESC-derived TPCs at day 22. Thus, this is a genetic edited model of tumor transformation derived from human ESCs-derived cells. Scientists have either generated in vitro a normal functional thyroid gland starting from murine ESCs^16^ or they have introduced oncogenic driver mutations in breast, colon, or pancreas organoids by using CRISPR/Cas9 technology to induce tumor transformation^50–52^. Nowadays, the most common genetic and epigenetic alterations reported in TC, lead to the constitutive activation of two main signaling pathways: *i)* the MAPK and *ii)* PI3K/AKT/mTOR signaling pathways, which promote uncontrolled cell division, cell migration and longevity^53^. The spectrum of altered genes, including point somatic mutations, which differ on TC histotypes, endorse tumor initiation and progression. *BRAF* is mutated in 35-40% of PTCs and in 25-35% of ATCs, while it is not mutated in FTCs. *RAS* point mutations are reported in 18-52% of FTCs and in 6-55% of ATCs^54^.

Accordingly, we show that *BRAF*^*V600E*^ or *NRAS*^*Q61R*^ recapitulates PTC and FTC histotypes, respectively, at transcriptomic, morphological and phenotypic levels. The mutation of tumor suppressor gene *TP53* rarely occurs in differentiated PTC and FTC while it is frequently reported in ATC (70%)^54,55^, positing that ATC can also arise from a *BRAF*-mutated PTC following a further acquisition in *TP53* mutations.

In line with these notions, our data reveal that *TP53* mutation alone is not sufficient to drive TC tumorigenesis, whereas only in association with *BRAF*^*V600E*^ or *NRAS*^*Q61R*^ generate undifferentiated TCs endowed with an enrichment of metastatic-associated gene signatures, contributing to the aggressive behavior that characterizes the ATC. This evidence ascertains that TC carcinogenesis follows a genetic mutation model, in which *NRAS*, *BRAF* and *TP53* mutations occur in TPCs.

By integrating transcriptomic analyses in primary and metastatic TC lesions of human patients and xenografts derived from TPCs harboring *BRAF*^*V600E*^ or *NRAS*^*Q61R*^ in combination with *TP53*^*R248Q*^ mutations, emerged TIMP1/MMP9/CD44 complex as fundamental for TC promotion, while the activation of KISS1/KISS1R signaling crucial for the metastatic growth. In accordance, *TP53*^*R248Q*^ alone was not able to induce the expression of the ternary complex TIMP1/MMP9/CD44. We previously demonstrated that cells expressing a CD44 isoform, CD44v6, display activation of PI3K/AKT and Wnt pathways, which cooperate to sustain their self-renewal, survival and metastatic potential^34,56,57^. TIMP1 promotes the activation of AKT in TPCs engineered with the selected point mutations, whereas its inhibition is associated with significantly decreased levels of CD44v6 and genes related to signaling of β-catenin, whose expression levels are positively correlated with TC poor prognosis^25^.

Moreover, we show that poor TC prognosis is associated with high expression levels of KISS1/KISS1R, which could be a promising functional marker for defining specific therapeutic targets and responsive advanced TC patients. The majority of non-metastatic differentiated TCs can be cured by surgical resection, radioactive iodine, and TSH suppression^3^, whereas metastatic TC patients are still incurable. Of note, KISS1R and TIMP1 inhibition in TPCs harboring *BRAF*^*V600E*^ or *NRAS*^*Q61R*^ in combination with *TP53*^*R248Q*^ mutations results in increased expression of NIS, thus restoring the functional radioiodine uptake. We propose that KISS1R and TIMP1 targeting may be further explored as adjuvant therapy to sensitize ATCs to the standard radiometabolic therapy.

Future research studies can take advantage of our experimental model based on the engineering of hESC-derived cells, even though artificial, to generate tumors with specific mutations in progenitor cells and to further investigate genes and pathways responsible of therapy resistance. It is likely that this model could be applied to study the tumorigenesis mechanisms in different organs derived from the definitive endoderm during their lineage differentiation. The engineering of hESC-derived TPCs can be exploited for both the generation of tumors with specific mutations, and the study of molecular signatures responsible for tumor transformation and therapy resistance.

## Methods

### Ethics statement

Institutional Review Board (IRB) approval for the collection of human tissues derived from TC patients was obtained from the Ethical Committee of the Mediterranean Institute of Oncology, Catania, Italy (authorization n° 16/21 on February 9th, 2021). The study complied with all the ethical regulations for work with human participants. Written informed consents were obtained from all the patients, including consent to publish information about age and sex.

### Human embryonic stem cell (hESC) culture and directed differentiation

The human embryonic stem cell (hESC) line, WA09 (#WA09-RB-001), was purchased by Wi Cell and cultured in mTESR1 media (Stemcell Technologies). Definitive endoderm was induced by culturing cells for five days with the StemDiff definitive endoderm kit (Stemcell Technologies). In order to induce anterior foregut endoderm differentiation, definitive endoderm cells were cultured for one additional day in serum-free medium, composed by 75% IMDM (ThermoFisher Scientific) and 25% Ham’s Modified F12 medium (EuroClone), N2 (ThermoFisher Scientific), B27 (ThermoFisher Scientific), BSA (0.05%, US Biological), L-glutamine (200 mM, Euroclone) and ascorbic acid (0.05 mg/ml, Sigma-Aldrich) (Serra, M. et al. 2017), supplemented with Noggin (100 ng/ml, Novus Biologicals) and the TGF-βRI, ALK4 and ALK7 inhibitor SB431542 (10 µM, Sigma-Aldrich). Lineage differentiation into thyroid progenitor cells was assessed by exposing cells, up to day 22, to the serum-free media containing FGF10 (10 ng/ml, Novus Biologicals), KGF (10 ng/ml, Peprotech), BMP4 (10 ng/ml, R&D), EGF (20 ng/ml, Peprotech), FGF2 (500 ng/ml, Peprotech) and heparin sodium salt (100 ng/ml, Sigma-Aldrich). For differentiation in mature thyrocytes, cells were cultured in serum-free medium, supplemented with FGF2 (100 ng/ml, Peprotech), FGF10 (100 ng/ml, Novus Biologicals), heparin sodium salt (100 ng/ml, Sigma-Aldrich) and TSH (1 mU/ml, Novus Biologicals). The ROCK inhibitor Y-27632 (10 µM, Sigma-Aldrich) was added after every cell passage. Cells at any stage of differentiation were seeded on matrigel-coated dishes, specifically by using the Corning hESC-qualified matrix up to day 6 and BD matrigel from day 6 to day 30. For all the experiments, cells at each stage of differentiation were maintained in the specific cell culture differentiation media.

BCPAP (catalog number ACC 273), TT2609co2 (catalog number ACC 510) and Cal62 (catalog number ACC 448), human PTC, FTC and ATC cells, respectively, were obtained from DSMZ and cultured according to the manufacturer’s instructions.

Cells were routinely authenticated performing short tandem repeat (STR) analysis, using a 24 loci-based multiplex PCR assay (GlobalFiler™ STR kit, Applied Biosystem).

The conditioned medium (CM) of hESCs-derived TPCs (D22) and their differentiation lineage (D30) was collected after exposure of specific medium to subconfluent cells for 48 h. Quantification of pro- and active MMP9 was performed using the quantikine ELISA kit (R&D systems). D22 TPCs were treated with TIMP1 (50 ng/ml; BioLegend), TIMP1 inhibitor (1 µg/ml; R&D), KISS1R inhibitor (10 nM; Tocris). Cytokines or inhibitors were added every 48 h to cell culture. D22 TPCs were pre-treated with Wnt-3a (200 ng/ml, R&D systems) and R-spondin1 (500 ng/ml) for 16 h. Then, TIMP1 inhibitor (1 µg/ml) was added for 48 h.

### Animals and tumor models

All the in vivo procedures complied with the institutional (University of Palermo) animal care committee guidelines (authorization #1281/2015-PR, Italian Ministry of Health). 4-6 weeks old female NOD.Cg-Prkdcscid/J (NOD SCID) mice were purchased by Charles River Laboratories and maintained in a barrier facility for animals in a temperature-controlled system characterized by 22 Celsius degrees and 50% humidity with a 12 h dark/light cycle within cages (Tecniplast) with radiation-sterilized bedding (SAWI Research Bedding, JELU-WERK). Mice were given *ad libitum* access to 0.45 μm-filtered tap water in sterile drinking bottles and to pelleted chow (Special Diets Services-811900 VRF1 (P)).

To evaluate the tumorigenic capacity of hESC-derived thyroid progenitor cells and mature thyrocytes, 3×10^6^ *BRAF*, *NRAS* and *TP53* engineered hESCs at different day of differentiation (D6/22/26/30) were resuspended in a 150 µl solution of 1:1 SCM/Matrigel (BD Biosciences) and subcutaneously injected in 4-6 weeks old female NOD.Cg-Prkdcscid/J (NOD SCID) mice (12 mice/group).

To assess the individual contribution of each member of the ternary complex in TC tumorigenesis process, 3×10^6^ hESC-derived cells at D22 and at D30 engineered for *NRAS*/*TP53* mutations and overexpressing *TIMP1, MMP9* and *CD44* alone and in combination were resuspended in a 150 µl solution of 1:1 SCM/Matrigel (BD Biosciences) and subcutaneously injected in 4-6 weeks old female NOD.Cg-Prkdcscid/J (NOD SCID) mice (10 mice/group).

To evaluate the effect of treatment with TIMP1 and KISS1R inhibitors, alone or in combination, 3,5×10^6^ *NRAS*/*TP53* engineered hESCs at D22 were resuspended in 150 µl of a solution composed of 1:1 SCM/Matrigel (BD Biosciences) subcutaneously injected in 4-6 weeks old female NOD.Cg-Prkdcscid/J (NOD SCID) mice (6 mice/group). Mice were immediately randomized in four groups and treated via intratumoral administration with KISS1R inhibitor (10 µM; Tocris) every day for 14 days and with a neutralizing antibody against TIMP-1 (1 µg/ml; R&D) once a week for 4 weeks alone or in combination.

Tumor size was monitored using a digital caliper twice per week and the volume determined by the formula: largest diameter x (smallest diameter)^2^ x π/6.

To evaluate the migration ability of D22 TPCs, bearing *BRAF* and *NRAS* mutations alone and in combination with *TP53*, 3×10^5^ cells were orthotopically injected into the thyroid gland of 4-6 weeks old female NOD.Cg-Prkdcscid/J (NOD SCID) (12 mice/group).

Mice sacrifice was performed in accordance with the Directive 2010/63/EU guidelines (D.lgs 26/2016) with the maximum diameter of the tumor =2 cm, or when any sign of suffering was observed. The maximal tumor size was not exceeded. Animals involved in the study were sacrificed at the pre-established end point or in presence of any morbidity sign. Mice at the experimental endpoint were subjected to rapid loss of consciousness through isoflurane inhalation and then sacrificed by cervical dislocation.

### CRISPR/Cas9 gene editing

In order to perform gene editing, cells of each stage of thyroid differentiation were transfected with CRISPR/cas9 all-in-one OFP vector (1 µg) and pMA-T customized donor DNA plasmid specific for *BRAF*^*V600E*^, *NRAS*^*Q61R*^ and *TP53*^*R248Q*^ mutations (1 µg) (ThermoFisher Scientific) by using the DNA transfection reagent (X-tremeGENE HP, Roche) according to the manufacturer’s instructions. The CRISPR Nuclease Vector with OFP Reporter (ThermoFisher Scientific) was used as nontargeting control (NTC). Following DNA incubation, transfection medium was replaced with the specific cell culture differentiation media. After 48 h, enrichment of OFP-positive cells was performed by FACSMelody cell sorter. All the in vitro and in vivo experiments, in cells of any stage of differentiation, have been performed at the same time upon transfection.

To detect *BRAF*^*V600E*^, *NRAS*^*Q61R*^ and *TP53*^*R248Q*^ mutations, DNA from engineered cells was extracted using the DNeasy Blood & Tissue Kit (Qiagen) and amplified by using the HotStarTaq Plus Master Mix Kit (Qiagen). Purification of amplified products was achieved with the MinElute PCR Purification Kit (Qiagen). BigDye Terminator v3.1 Cycle Sequencing Kit and BigDye X-Terminator Purification Kit (Applied Biosystems) were used for the purification and base pair sequence, respectively. Capillary electrophoresis was performed by ABI PRISM 3130 Genetic Analyzer or by BMR Genomics service (Padua, Italy). The obtained electropherograms were visually analyzed using 4Peaks Software (Griekspoor and Tom Groothuis, nucleobytes.com). The Synthego online tool (https://design.synthego.com/#/validate) was used to predict potential off-target sites for gRNAs and the top three predicted off-targets were analyzed by DNA sequencing.

All the primers used for sequencing analysis were listed in Supplementary Table 3.

### DNA extraction and next generation sequencing (NGS)

DNA was extracted from engineered cells using DNeasy Blood & Tissue Kit (Qiagen) according to manufacturer’s instructions. Yields of extracted DNA were assessed by dsDNA HS Assay kit on Qubit 3.0 Fluorometer (both from ThermoFisher Scientific). Libraries were prepared using a custom primer panel designed with the Ion AmpliSeq Designer tool (https://www.ampliseq.com/login/login.action). The panel encompasses 1525 amplicons covering 25 genes, including *BRAF*, *TP53* and *NRAS* genes. NGS libraries were generated with Ion AmpliSeq library kit plus (ThermoFisher Scientific) and the customized panel starting from 10 nanograms of genomic DNA for each primer pool. The libraries were barcoded employing the Ion Xpress barcode adapter kit (ThermoFisher Scientific), quantified by qPCR with the Ion Library TaqMan Quantitation kit (ThermoFisher Scientific), and diluted to equimolar amounts before pooling. The Ion Chef System was used for automated template preparation and chip loading. Sequencing was carried out on Ion GeneStudio S5 Plus System using the Ion 510 & Ion 520 & Ion 530 Kit (all from ThermoFisher Scientific). Raw data were then aligned to the hg19 reference genome and Torrent Suite v.5.10.1 (ThermoFisher Scientific) was used to perform initial quality control, including chip loading density, median read length and number of mapped reads. Ion Reporter v5.18.2.0 (ThermoFisher Scientific) was employed to single nucleotide variant (SNV) annotations. Cut offs of 3% for variant allele frequency (VAF), read depth > 100, Phred quality score >40 and *p* value <0.0001 were used for filtering in order to exclude false positive variants.

*BRAF*^*V600E*^, *NRAS*^*Q61R*^ and *TP53*^*R248Q*^ mutation percentage in h-ESCs-derived cells at different stage of thyroid differentiation lineage detected by Sanger and NGS technologies are reported in Supplementary Table 1.

### Patients

Fresh frozen or formalin-fixed paraffin-embedded (FFPE) tissues from 97 TC patients, including 73 PTC primary tumors (PTC P; ID#1-73), 19 loco-regional lymphnode metastases (PTC M; ID#74-92), and 5 ATCs who underwent thyroidectomy were collected and analyzed in accordance with the ethical policy of the Mediterranean Institute of Oncology on Human Experimentation. The clinicopathological characteristics of TC patients are shown in Supplementary Table 2 and their mutational background evaluated by Next Generation Sequencing (NGS) analysis is provided in Supplementary Data 2.

### Flow cytometry analysis

Cycle analysis of hESCs-derived cells at different stage of thyroid differentiation was analyzed by incubating cells with 1 ml of Nicoletti Buffer (0.1% of Sodium citrate, 0.1% of Triton x-100, 50 μg/ml of Propidium Iodide, 10 μg/ml of Rnase solution) in the dark at 4 °C overnight. DNA content was evaluated by BD FACS Lyric flow cytometer (BD Clinical system, BD Biosciences).

For FACS analysis, hESCs-derived TPCs were washed in PBS and exposed for 1 h at 4 °C to CD44v6 (2F10 APC, mouse IgG1, R&D systems, 5 μl/sample), Oct3/4 (40/Oct-3 Alexa-fluor647, mouse IgG1K, BD Biosciences, 20 μl/sample), Sox2 (245610 PE, mouse IgG2a BD Biosciences, 20 μl/sample), Nanog (N31-355 PE, mouse IgG1, BD Biosciences, 20 μl/sample), CXCR4 (FAB170P PE, mouse IgG2a, R&D system, 1 μl/sample), c-kit (YB5.B8 PE, mouse IgG1 BD Biosciences, 20 μl/sample), Sox17 (P7-969 PE, mouse IgG1k, BD Biosciences, 5 μl/sample), Fox A2 (N17-280 PE, mouse IgG1, BD Biosciences 5 μl/sample), PAX8 (PAX8/1492 APC, mouse IGg2a, Novus 5 μl/sample), TTF1 (REA1090 FITC, mouse IgG1, MACS Miltenyi Biotec, 16 μl/sample), TSH-R (4C1 FITC, IgG2a Santa Cruz, 2 μl/sample), TPO (203340, Antirabbit IgG H + L Alexa-488, Abcam, 2 μl/sample), Thyroglobulin (SPM221 PE, mouse IgG1, Novus, 2 μl/sample), NIS (SPM186, goat antimouse IgG (H + L) Alexa-488, Abcam, 5 μl/sample), CD133 (W6B3C1 FITC, mouse IgG1, BD Bioscience, 20 μl/sample), ABCG2 (5D3/CD338 APC, mouse IgG2b, BD Bioscience, 10 μl/sample), Nestin (25/Nestin PerCP 5.5, mouse IgG1, BD Bioscience, 5 μl/sample), HNF-4α (H-1, goat anti-mouse IgG (H + L) Alexa-488, Santa Cruz, 4 μl/sample), or corresponding isotype matched controls (IMC) and analyzed using the FACS Lyric cytometer (BD Biosciences). All the listed antibodies have been validated following manufacturer’s information, using appropriate positive and negative controls. To perform antibody titration 4 different dilutions were tested starting from the manufacturer’s recommended concentration, including the 0.5 and 2x concentrations. Titration of the antibodies has been routinely performed at antibody arrival, and every 3 months after storage.

Tissues from normal, PTC, FTC and ATC thyroids were digested mechanically and enzymatically with collagenase (Life Technologies) and hyaluronidase (Sigma Chemical Co.) and thyrocytes were isolated, purified and cultured in DMEM 10% fetal bovine serum (FBS) (Life Technologies)^58^. Following enzymatic dissociation with Accutase (A1110501, ThermoFisher), 1×10^5^ cells were stained in 200 µl of staining buffer (PBS with 0.5% BSA) with specific fluorescence-conjugated antibody, or the corresponding, according to the manufacturer’s recommendations. For the detection of intracellular markers, cells were fixed in 2% paraformaldehyde and permeabilized using PBS 0.1% Triton X-100. The dead cells were excluded using the 7-AAD (BD Biosciences). Data were analyzed by FlowJO software.

### Cell proliferation, clonogenic and invasion assay

The proliferative potential of hESC at different day of thyroid differentiation was assessed by using Tripan blue exclusion test.

Clonogenicity of TPCs bearing different mutations was determined by plating 1, 2, 4, 8, 16, 32, 64, 128 cells per well and evaluated with the Extreme Limiting Dilution Analysis (ELDA) ‘limdil’ function (http://bioinf.wehi.edu.au/software/elda/index.html). The invasive potential of hESCs (D6, D22, D26, D30) and of *BRAF/TP53* and *NRAS/TP53* mutated TPCs, pretreated with vehicle, TIMP1, TIMP1 inhibitor (TIMP1i) alone or in combination, was estimated by seeding 2×10^3^ into 8 µm pore size transwell coated with growth factor reduced matrigel (BD Biosciences). Advanced DMEM/F12 medium (Thermo fisher) supplemented with 10% FBS (Lonza) was used as chemoattractant in the lower chamber of transwell. Invading cells were calculated microscopically up to 72 h.

### Cell transfection and lentiviral transduction

Production of lentiviral particles was achieved through the transfection of HEK-293T packaging cells (catalog number CRL-3216, obtained from ATCC), with lentiviral vector expressing *TIMP1*, *MMP9*, *CD44* (Horizon Discovery) and *CD44v6*^34^ or with a lentiviral plasmid carrying a specific shCD44v6^34^, together with psPAX2 (Addgene) and pMD2.G (Addgene) in OPTIMEM (Gibco) medium supplemented with DNA transfection reagent (XtremeGENE HP, Roche). pLOC (Horizon Discovery) and pTWEEN-GFP plasmids or scrambled (PLK0.1, Addgene) vector was used as control, respectively. Lentiviral transduction was performed by exposing hESCs (D22, D30) to lentiviral particles in presence of polybrene (8 μg/ml) (Sigma-Aldrich). Selection of resistant clones, where suitable, was performed by treating cells with puromycin (0.5 μg/ml) (Sigma-Aldrich).

### Immunohistochemistry and immunofluorescence

Immunohistochemical analysis was performed on 4-µm-thick paraffin-embedded tumor sections using specific antibody against Tg (EPR9730, rabbit monoclonal, Abcam, 1:100 dilution), CK19 (B170, mouse IgG1, Leica, 1:50 dilution), β-catenin (E-5, mouse IgG1,k, Santa Cruz, 1:25 dilution), NIS (SPM186, mouse IgG1,k, Abcam, 1:50 dilution), TIMP1 (M7293, mouse IgG1,k, Dako, 1:50 dilution), CD63 (MX-49.129.5, mouse IgG1,k, Santa Cruz, 1:50 dilution), MMP9 (4A3, mouse IgG1, ThermoFisher, 1:100 dilution), CD44v6 (2F10, mouse IgG1, R&D systems, 1:100 dilution), CD44 (156-3c11, mouse monoclonal, Cell Signaling, 1:50), Twist (Twist2C1a, mouse IgG1, Abcam, 1:25 dilution), Snail (63371, rabbit polyclonal, Abcam, 1:50 dilution), KISS1 (34010, rabbit polyclonal, Novus Bio, 1:200 dilution), KISS1R (TA351332, rabbit polyclonal, Origene, 1:50 dilution), TSHR (4C1, mouse IgG2a κ, Santa Cruz, 1:50 dilution), S100 (GA504, rabbit polyclonal, Dako, 1:1000 dilution), CDX2 (AMT28, mouse monoclonal IgG1, Novocastra, 1:50 dilution), Oct3/4 (C-10, mouse IgG1, Santa Cruz, 1:200), p40 (PA0163, Leica), P53 (DO-7, mouse monoclonal IgG2b, Novocastra, 1:100 dilution). Single stainings were revealed by the byotine-streptavidine system (Dako). Antibody detection was performed using the 3-amino-9-ethylcarbanzole (Dako). Romlin (bio-optica) has been used only for KISS1 staining. Aqueous hematoxylin (Sigma) was used to counterstain nuclei.

Immunostaining intensity has been calculated by Image J software (Analyze Histogram), by analyzing 5 serial sections from 3 tumor xenografts or patient-derived tumors. Arbitrary unit intensity has been normalized on cell count and expressed as fold variation over control. Immunohistochemistry score of KISS1R expression has been measured as the percentage of positive cells (1 = 1–12%; 1.5 = 13–25%; 2 = 26–38%; 2.5 = 39-50%; 3 = 51–62%; 3.5 = 63–75%; 4 = >75%). H&E staining was performed using standard protocols.

For immunofluorescence, ESC-derived thyroid follicles grown on matrigel droplets or D22 TPCs grown on coverslips, were fixed, and exposed overnight at 4 °C to the following antibodies: NIS (SPM186, mouse IgG1,k, Abcam), CK19 (B170, mouse IgG1, Leica), TSHR (4C1, mouse IgG2a κ, Santa Cruz) and KISS1R (NBP2-57942, rabbit polyclonal, Novus). Primary antibody staining was revealed by using antirabbit or antimouse secondary antibody conjugated with Alexa Fluor-488 (Life Technologies). Nuclei were counterstained using Toto-3 iodide (Life Technologies). NIS positivity was determined by using Image J software (Analyze Histogram), normalized on cell count (Analyze Particles) and expressed as arbitrary units (AU). At least 3 fields for each sample were counted. All the reported antibodies for both immunohistochemistry and immunofluorescence analyses have been validated following manufacturer’s information, using appropriate positive and negative controls.

### Western blot

hESCs-derived TPCs and their differentiation lineage (D30) were collected and washed twice with ice-cold PBS, before being lysed using a buffer containing 10 mM Tris-HCL (Sigma-Aldrich), 50 mM NaCl (Sigma-Aldrich), 30 mM sodium pyruvate (Sigma-Aldrich), 50 nM NaF (Sigma-Aldrich), 5 µM ZnCl_2_ (Sigma-Aldrich), 1% triton (Bio-Rad) supplemented with protease and phosphatase inhibitor cocktails (Sigma-Aldrich), 0.1 nM sodium orthovanadate (Sigma-Aldrich), 10 mM sodium butyrate (Sigma-Aldrich) and 1 mM PMSF (Sigma-Aldrich). Total protein extracts were loaded onto SDS-PAGE gel and blotted on nitrocellulose membranes. Following the incubation with blocking solution (5% Blotto, not fat dry milk, Santa Cruz Biotechnology, PBS 0.1% Tween 20), membranes were exposed overnight at 4 °C to β-catenin (Ser33/37/Thr41, D13A1, rabbit IgG, CST, 1:500 dilution), TIMP1 (M7293, mouse IgG1,k, Dako, 1:400 dilution), CD44 (156-3C11, mouse IgG2a, CST, 1:500 dilution), phospho-AKT XP (Ser473; D9E, rabbit, IgG, CST, 1:1000 dilution), AKT (rabbit polyclonal, CST, 1:500 dilution), NIS (SPM186, mouse IgG1,k, Abcam, 1:1000 dilution), phospho-ERK 1/2 (Thr202/Tyr204; rabbit polyclonal, CST, 1:500 dilution), ERK 1/2 (137F5, rabbit IgG, CST, 1:1000 dilution). For protein expression normalization, β-actin (8H10D10, mouse, CST, 1:1000 dilution) was used as endogenous control.

All the listed antibodies have been validated following manufacturer’s information, using appropriate positive controls. Anti-mouse or anti-rabbit HRP-conjugated antibodies (goat H + L, ThermoFisher Scientific) were used to reveal primary antibodies, which were finally detected using the Amersham imager 600 (GE Healthcare).

### RNA extraction, real-time/digital PCR and RNA sequencing

Total RNA of hESCs-derived TPCs (D22) and their differentiation lineage (D26 and D30), engineered with *BRAF*^*V600E*^, *NRAS*^*Q61R*^, *TP53*^*R248Q*^, *BRAF*^*V600E*^*/TP53*^*R248Q*^ and *NRAS*^*Q61R*^*/TP53*^*R248Q*^ CRISPR vectors, or frozen primary and metastasis tissue xenografts obtained from the injection of *BRAF*^*V600E*^*/TP53*^*R248Q*^ D22 cells was obtained using TRIZOL reagent (Thermo Fisher Scientific). Total RNA from FFPE PTC and ATC primary and metastasis tissues was purified by using RNeasy FFPE Kit (Qiagen) following manufacturer’s instructions. For gene expression array, 1 µg of total RNA was retrotranscribed and analyzed with a PrimePCR EMT and stemness-related gene custom panel (Bio-Rad). For Real-time PCR, RNA was retrotranscribed using the High-Capacity cDNA Reverse Transcription Kit (Applied Biosystems) and qRT-PCR was performed using specific primers. Quantitative Real-time PCR analysis was performed in a Taqman master mix (Qiagen 1054588) containing the primer sets Hs01075870_m1 (*CD44v6*), Hs00765553_m1 (*CCND1*) and Hu-*GAPDH* (Applied Biosystems), or in a SYBR Green Master Mix (Qiagen, 1054586) using primers shown in Supplementary Table 4.

Relative mRNA expression levels were obtained using the comparative CT method (ΔΔCt method). The cycle threshold (Ct) of the housekeeping genes *GAPDH* or *HPRT1* or their average was used to normalize the data. Heatmaps were generated using ComplexHeatmap (v2.6.2) and show 2^-ΔCt^ values, row scaled per array. Venn diagrams were plotted through the VennDiagram (v1.6.20) R package. Gene expression data of *KISS1* and *KISS1R* in thyroid cancer patients (“Tumor Thyroid Carcinoma All” database) were obtained from R2: Genomics Analysis and Visualization Platform (https://hgserver1.amc.nl/cgi-bin/r2/main.cgi). Analysis of enriched pathways from the Reactome database was performed through EnrichR R library v3.0.

Total RNA from FFPE thyroid tumor tissue specimens was purified with RNeasy FFPE Kit (Qiagen). 200 ng of total RNA were retrotranscribed with the high-capacity c-DNA reverse Transcription kit (Applied Biosystem).

Droplet digital PCR (ddPCR) was performed using ddPCR supermix for probes no-dUTP (Bio-Rad), and *KISS1R* (FAM) and *GAPDH* (HEX) ddPCR GEX assays (Bio-Rad). Following droplets generation and RT-PCR amplification, samples were quantified using QX200 Digital Droplet Reader (Bio-Rad). The obtained data were analysed with QuantaSoft Software (Bio-Rad) and *KISS1R* expression levels were calculated using the *KISS1R* (copies/μl)/*GAPDH* (copies/μl) ratio. Total RNA-seq analysis was carried out on 28 samples, including engineered D22 TPCs, D22-derived xenograft tumor tissues and human tissues from primary tumors and metastatic lesions of PTC and ATC patients. Read alignment and gene expression quantification were performed using STAR and Stringtie, respectively. Differentially expressed genes (DEGs) analysis was conducted by R with edgeR library based on the following criteria: (1) adjusted *P* < 0.05 and (2) Log 2 FC ≥ 2. The top 100 DEGs were selected. Gene set enrichment analysis (GSEA) was performed by using R library msigdbr and querying the hallmark (H) category, which includes 50 pathways. Pathways statistically significant with *p* value <0.05 were selected. Genes involved in PI3K/AKT-, MAPK-, EMT-related pathways were extracted from REACTOME signatures from MSigDB. Heatmaps were plotted using R library ggplot2 and ComplexHeatmap.

### Nonradioactive assay

Iodine uptake of D22 TPCs, D30 and BCPAP cells has been measured by using Nonradioactive Iodine Assay kit (#D05076.96 wells; Bertin Pharma, Cayman Chemical), according to the manufacturer’s specifications. D22 TPCs, D30 and B-CPAP cells were plated (500000 cells in 24-well plates) and treated with KISS1R inhibitor (10 nM; Tocris) and TIMP1 inhibitor (1 µg/ml;R&D) alone or in combination for 48 h. Then, cell supernatant was analysed using Nonradioactive Iodine Assay kit (#D05076.96 wells; Bertin Pharma, Cayman Chemical).

### Radioactive assay

D22 TPCs, B-CPAP, TT2609co2 and Cal62 cells were plated (50,000 cells in 24-well plates) and treated with KISS1R inhibitor (10 nM; Tocris) and TIMP1 inhibitor (1 µg/ml;R&D) alone or in combination for 72 h. Then, cells were incubated with iodine-125 (125I) (0.05 mCi; PerkinElmer). After 1 h, cells were washed twice in HBSS (Sigma-Aldrich) and lysed in 100 ml 2% SDS. Radioiodine uptake was determined using PerkinElmer Wallac 1470 Wizard instrument. Iodine 125 uptake was calculated normalized the counts per minute on the relative protein concentration revealed using Protein assay dye reagent concentrate (BIO-RAD).

### Statistical analysis

Kruskal-Wallis test and pairwise Wilcoxon tests were used to compare the expression levels of *CTNNB1* in normal thyrocytes, papillary and anaplastic thyroid carcinomas from the GSE33630 series in Gene Expression Omnibus (GEO). The Wilcoxon test was used to compare the expression levels of *TIMP1*, *MMP9* and *CD44* in normal and tumorigenic samples from the The Cancer Genome Atlas (TCGA) thyroid carcinoma (THCA) branch. TNMplot database was used for the analysis of thyroid cancer RNAseq-based data, for TIMP1/MMP9/CD44 and KISS1R gene expression analysis, respectively, using Kruskal-Wallis test.

Survival analyses were performed on data obtained from Gene Expression Profiling Interactive Analysis (GEPIA http://gepia.cancer-pku.cn/), showing the hazards ratio based on Cox PH Model, and expressed as disease-free survival (DFS) curves.

Following Kolmogorov-Smirnov test to assess the samples distribution, statistical significance was estimated by unpaired two-tailed t test, or by two-tailed Mann–Whitney test.

### Reporting summary

Further information on research design is available in the Nature Portfolio Reporting Summary linked to this article.

## Supplementary information

Supplementary Information

Reporting Summary

Description of Additional Supplementary Files

Supplementary Data 1

Supplementary Data 2

## Data Availability

All data relevant to the study are included in the article, Supplementary Information, and Source Data. NGS sequencing data from hESC-derived cells at different stage of thyroid differentiation lineage and from 93 TC patients, as well as total RNA-seq trancriptomic data of engineered D22 TPCs, D22-derived xenograft tumor tissues and human tissues from primary tumors and metastatic lesions of PTC and ATC patients, generated in this study, have been deposited in a public open-access GEO repository under accession code BioProject ID PRJNA887246. *CTNNB1* expression values were obtained from the GSE33630 series in Gene Expression Omnibus (GEO). *TIMP1, MMP9* and *CD44* expression levels were obtained from The Cancer Genome Atlas (TCGA) thyroid carcinoma (THCA) branch. *KISS1* and *KISS1R* expression values were obtained from R2: Genomics Analysis and Visualization Platform (https://hgserver1.amc.nl/cgi-bin/r2/main.cgi). TNMplot database was used for the analysis of thyroid cancer RNAseq-based data, for TIMP1/MMP9/CD44 and KISS1R gene expression analysis, respectively. Survival analyses were performed on data obtained from Gene Expression Profiling Interactive Analysis (GEPIA http://gepia.cancer-pku.cn/). Data about the histotypes and the mutational background of TCs derived from patients along the whole manuscript, are provided in Supplementary Data 1 and 2. Source data are provided with this paper.

## Source data

Source Data
