# Supplementary material for: Recapitulating thyroid cancer histotypes through engineering embryonic stem cells
Source: Nat Commun. 2023 Mar 11;14:1351. doi: 10.1038/s41467-023-36922-1 (PMC10008571; doi:10.1038/s41467-023-36922-1)
Supplement: Supplementary file 1 — Supplementary Information [file 41467_2023_36922_MOESM1_ESM.pdf]

Supplementary Information

Recapitulating thyroid cancer histotypes through engineering embryonic stem cells

Veronica Veschi, Alice Turdo, Chiara Modica et al.

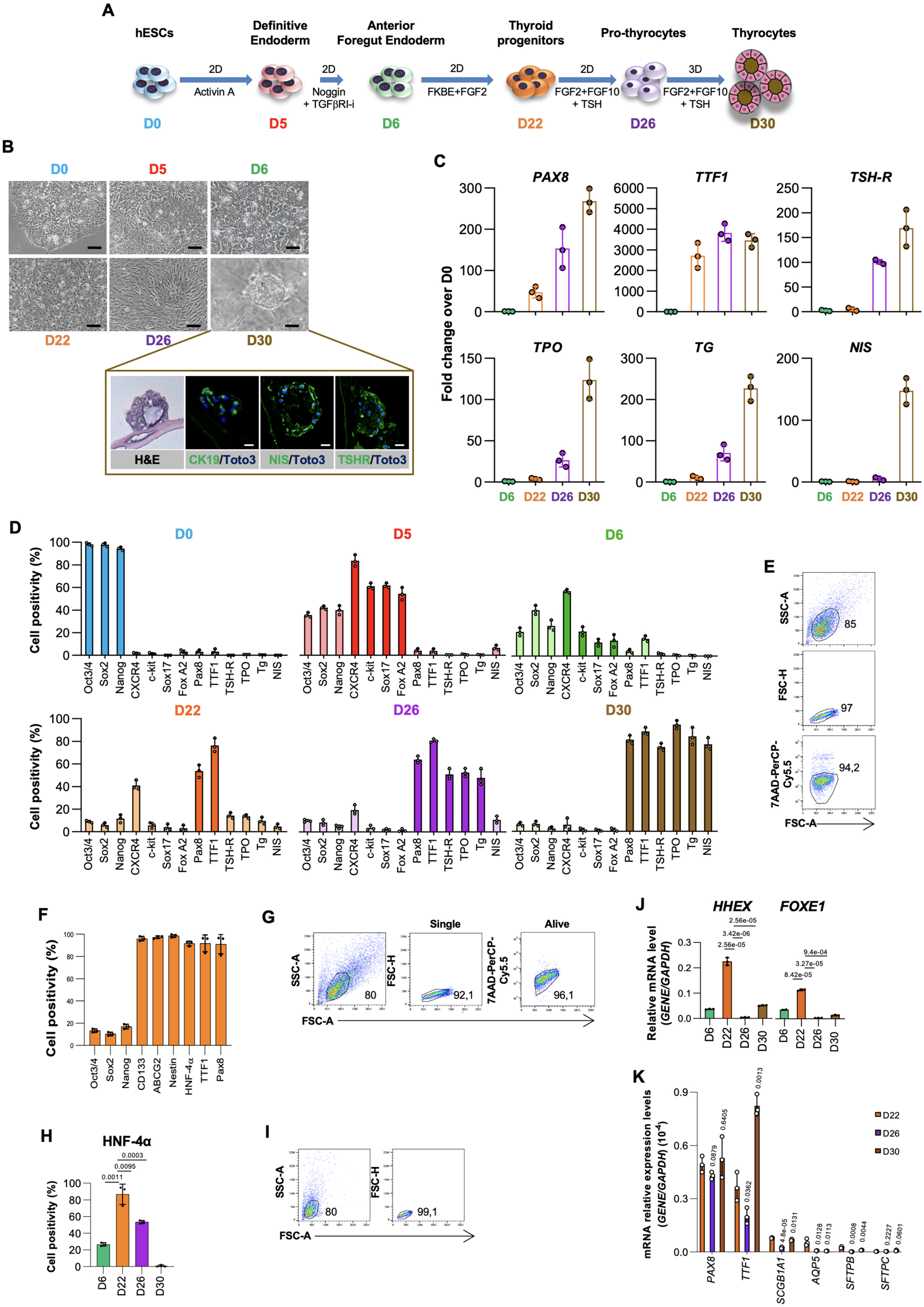

Veschi, Turdo, Modica et al. Supplementary Figure 1

Supplementary Figure 1. Thyroid progenitor cells harboring the most common TC mutations resemble the phenotypic characteristics of thyroid tumors.

a, Scheme illustrating the different stages of thyroid differentiation lineage. To promote thyroid lineage specification, human embryonic stem cells (hESCs) were exposed to the indicated stimuli at day 5 (definitive endoderm, D5), day 6 (anterior foregut endoderm, D6), day 22 (thyroid progenitors, D22), day 26 (pro-thyrocytes, D26) and day 30 (thyrocytes, D30). Treatment with FGF10, KGF, BMP4 and EGF is reported as FKBE. **b,** (*upper panel*) Phase contrast microscopy analysis of hESCs at the indicated stage of thyroid differentiation lineage. Scale bars, 20 µm. (*inset*) H&E and immunofluorescence analysis of cytokeratin 19 (CK19), sodium iodide symporter (NIS) and thyroid stimulating hormone receptor (TSHR) in thyrocytes. Nuclei were counterstained by Toto-3. Scale bars, 20 µm. One representative of three independent experiments is shown. **c,** Relative mRNA expression levels of *PAX8, TTF1, TSH-R, TPO, TG* and *NIS* in hESCs at the indicated stage of thyroid differentiation lineage. Data are mean ± SD of three independent experiments. **d,** Flow cytometry analysis of the indicated markers in hESCs at D0, D5, D6, D22, D26 and D30 stage of thyroid differentiation lineage. Data are mean ± SD of three independent experiments. **e,** Representative gating strategy for flow cytometry analysis showed in (d). **f,** Flow cytometry analysis of the indicated markers in D22 TPCs. Data are mean ± SD of three independent experiments. **g,** Representative gating strategy for flow cytometry analysis showed in (f). **h,** Flow cytometry analysis of HNF-4α in hESCs at the indicated stage of thyroid differentiation lineage. **i,** Representative gating strategy for flow cytometry analysis showed in (h). **j,** mRNA levels of *HHEX* and *FOXE1* in hESCs at the indicated stage of thyroid differentiation lineage. For (**h** and **j**) data are mean ± standard error of three independent experiments. **k,** Relative mRNA expression levels of *PAX8, TTF1, SCGB1A1, AQP5, SFTPB* and *SFTPC* at the indicated stage of thyroid differentiation lineage. Data are mean ± SD of three independent experiments. For (**h, j** and **k**) statistical significance was calculated using the unpaired two-tailed t test.

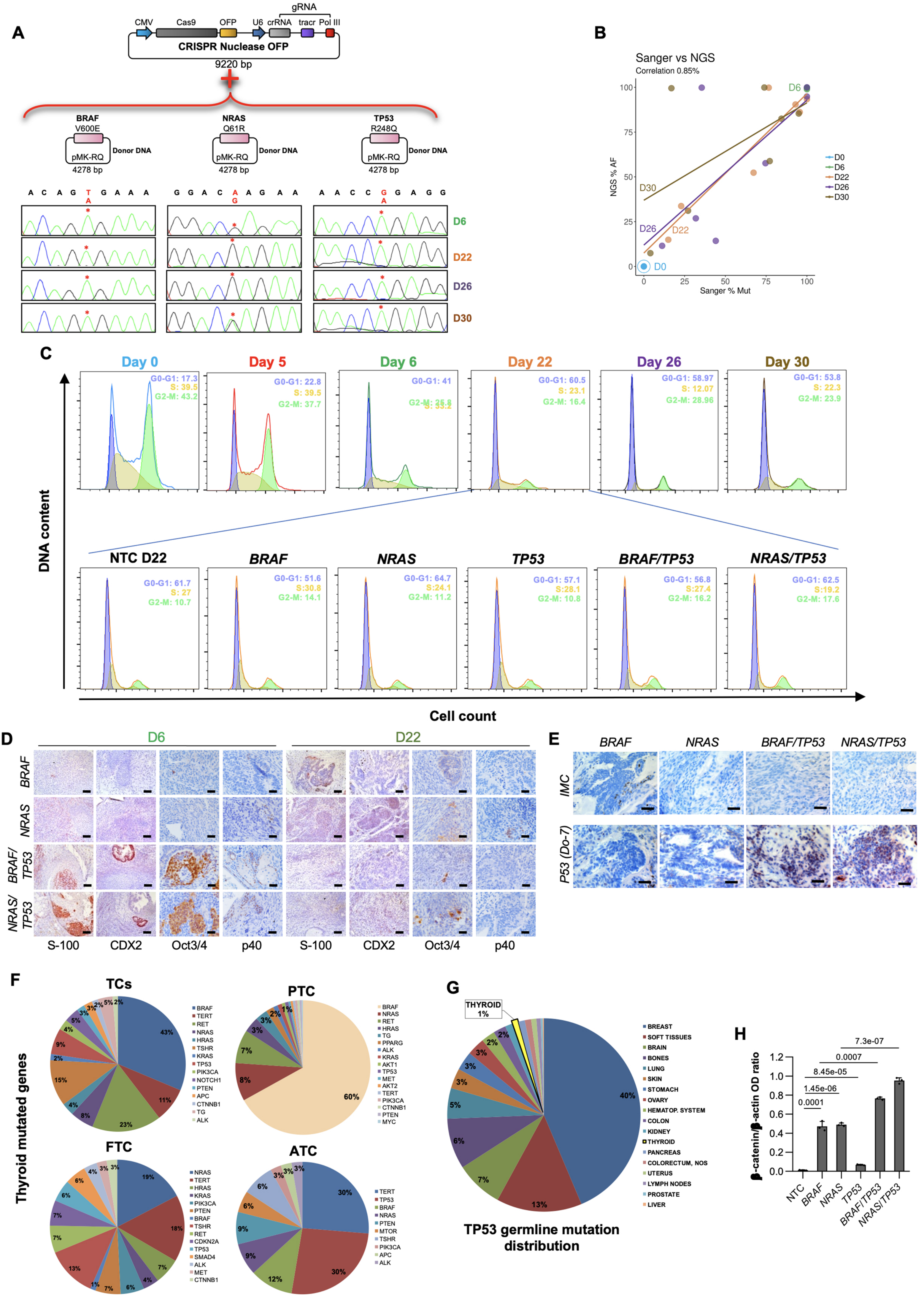

Veschi, Turdo, Modica et al. Supplementary Figure 2

Supplementary Figure 2. Characterization of CRISPR-cas9 engineered TPCs.
a, (*upper panel*) Gene editing strategy for the knock-in of $BRAF^{V600E}$, $NRAS^{Q61R}$ and $TP53^{R248Q}$ mutations based on the use of CRISPR/Cas9 Nuclease vector with OFP reporter and the specific donor DNA. (*lower panel*) Electropherograms showing the nucleotide sequence of interest in the DNA of cells carrying the indicated mutations at the indicated stage of thyroid differentiation lineage. **b,** Correlation plot of Next Generation and Sanger sequencing data in hESCs at D0 and engineered thyroid differentiation lineage at D6, D22, D26 and D30. One representative of three independent experiments is shown. **c,** Flow cytometry analysis of cell cycle in hESCs at the indicated stage of thyroid differentiation lineage, and in D22 TPCs harboring the indicated mutations. Plots show the percentage of cells in G0-G1 (blue box), S (yellow box) and G2-M (green box) cell cycle phases. **d,** Immunohistochemical analysis of S-100, CDX2, Oct3/4 and p40 on xenograft tumors obtained following the injection of hESCs at D6 or D22 of thyroid differentiation lineage engineered with different mutation background. Scale bars, 200 μm. **e,** Immunohistochemical analysis of P53 (Do-7) on D22 TPCs-derived tumor xenografts generated as in (c). IMC= Isotype matched control. Scale bars, 100 μm. One representative of three independent experiments is shown. **f,** Pie charts illustrating the frequency of thyroid mutated genes in all TCs, PTC, FTC and ATC from TGCA database. **g,** Pie chart showing *TP53* germline mutation distribution by tumor type in a cohort of patients affected by Li-Fraumeni syndrome. Data have been analyzed from TGCA database. **h,** Optical density ratio of β-catenin expression levels in D22 TPCs engineered with the indicated mutations. Statistical significance was calculated using the unpaired two-tailed t test. Data are mean ± SD of three independent experiments.

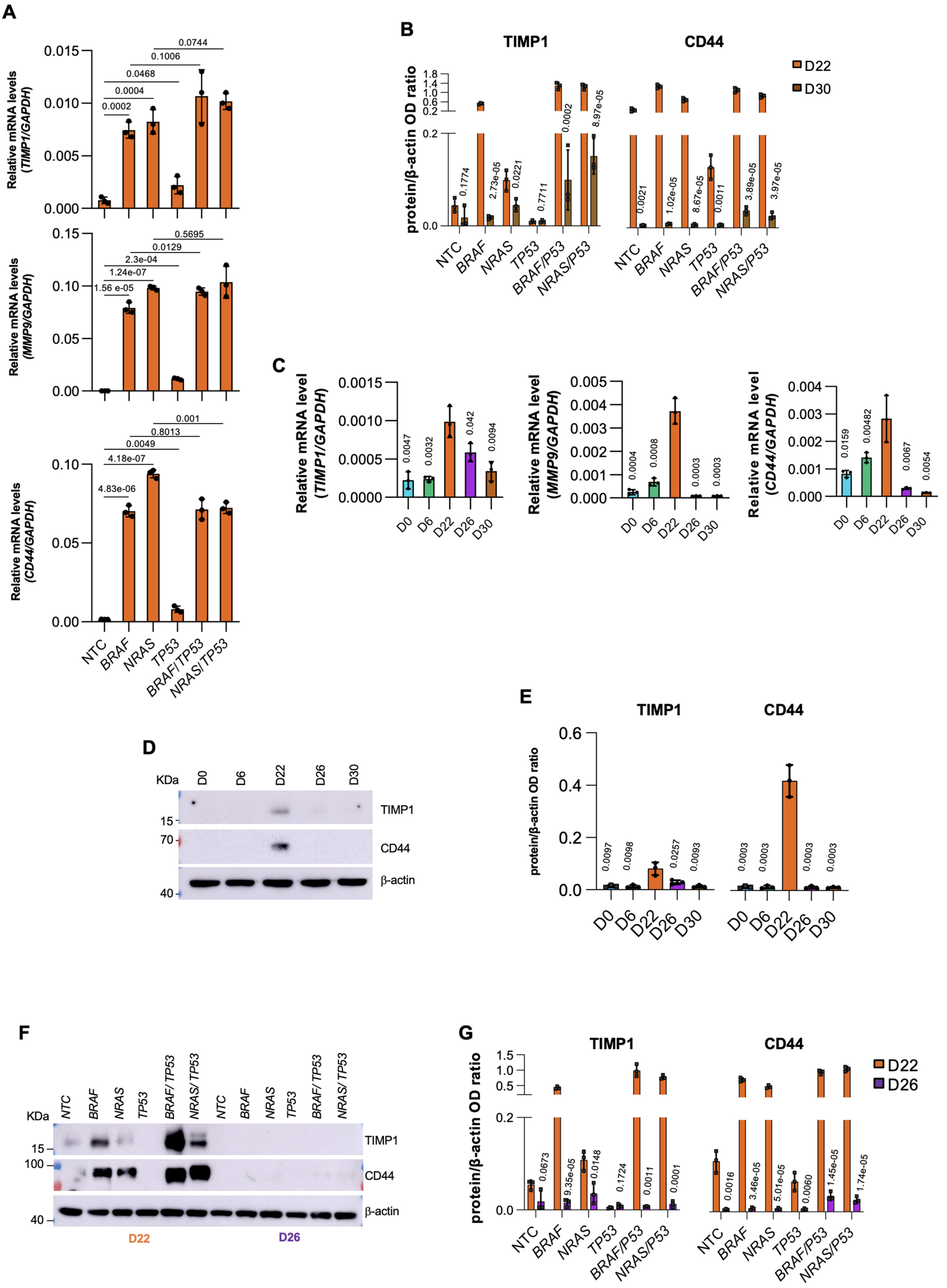

Veschi, Turdo, Modica et al. Supplementary Figure 3

Supplementary Figure 3. The ternary complex TIMP1-MMP9-CD44 is expressed at elevated levels in D22 TPCs.

a, Relative mRNA expression levels of *TIMP1, MMP9* and *CD44* in D22 TPCs engineered with the indicated mutations. **b,** Optical density ratio of TIMP1 and CD44 in hESC-derived cells harboring different mutational background, at D22 and D30 of thyroid differentiation lineage. **c,** Relative mRNA expression levels of *TIMP1, MMP9* and *CD44* in hESCs at D0 and thyroid differentiation lineage at D6, D22, D26 and D30. For (**a**–**c**) statistical significance was calculated using the two-tailed unpaired t test and data are mean ± standard error of three independent experiments. **d,** Immunoblot analysis of TIMP1 and CD44 in hESCs at D0 and thyroid differentiation lineage at D6, D22, D26 and D30. β-actin was used as loading control. Source data are provided as a Source Data file. **e,** Optical density ratio of TIMP1 and CD44 in hESCs at D0 and thyroid differentiation lineage at D6, D22, D26 and D30. Statistical significance was calculated using the unpaired two-tailed t test. Data are mean ± SD of three independent experiments. **f,** Immunoblot analysis of TIMP1 and CD44 in hESC-derived cells harboring different mutational background, at D22 and D26. β-actin was used as loading control. Source data are provided as a Source Data file. **g,** Optical density ratio of TIMP1 and CD44 in cells as in (f). Statistical significance was calculated using the unpaired two-tailed t test. Data are mean ± SD of three independent experiments.

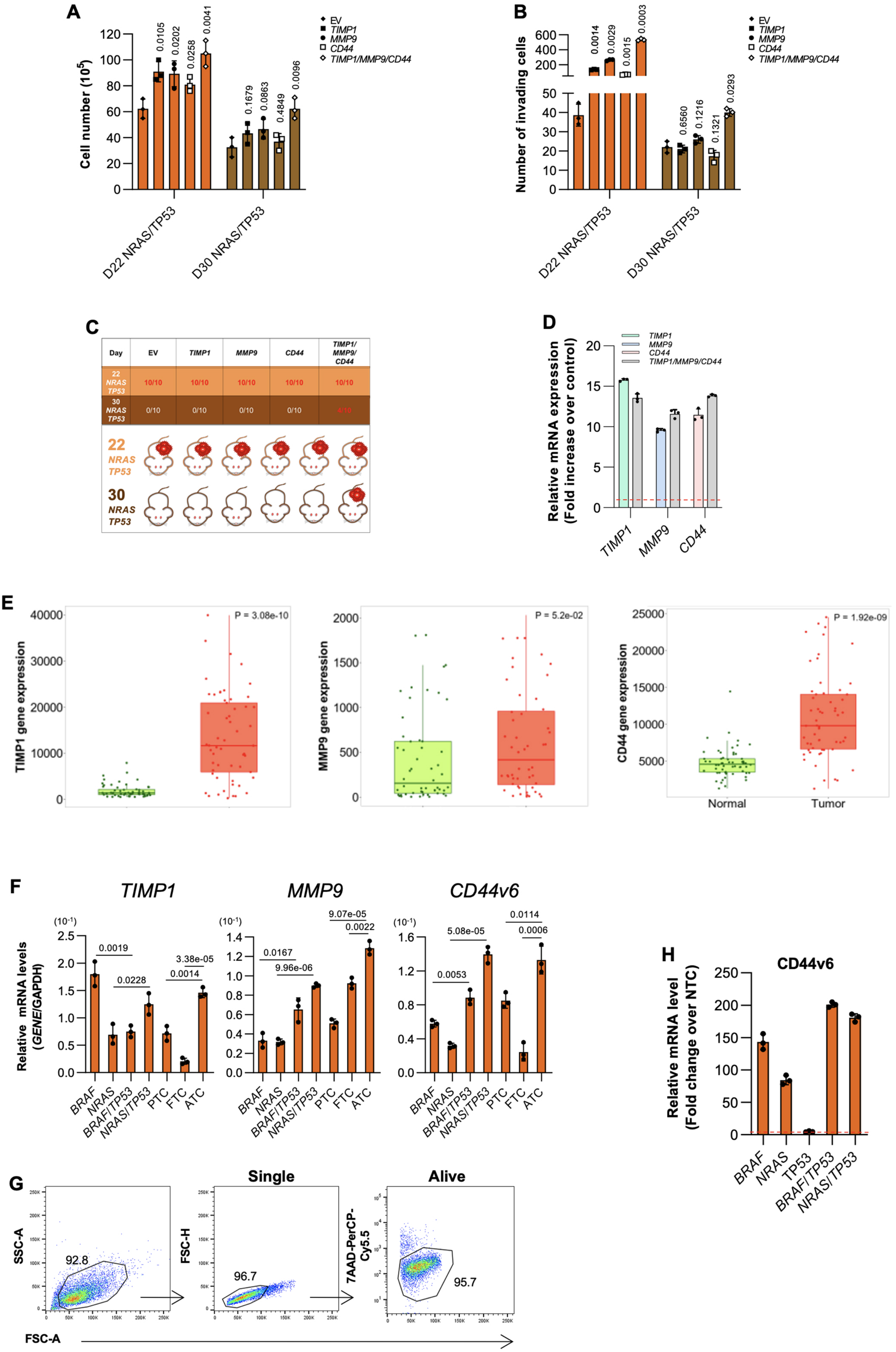

Veschi, Turdo, Modica et al. Supplementary Figure 4

Supplementary Figure 4. TIMP1, MMP9 and CD44v6 cooperate in promoting TC tumorigenesis.

a, Cell proliferation of the indicated engineered D22 TPCs and D30 cells overexpressing TIMP1, MMP9 and CD44 alone or in combination, at 7 days. **b.** Invasion analysis of cells as in (a) at 72 hours. For (**a** and **b**) statistical significance was calculated using the two-tailed unpaired t test and data are mean ± standard error of three independent experiments. **c,** Frequency of TCs obtained by the injection of the indicated engineered D22 TPCs and D30 cells overexpressing *TIMP1*, *MMP9* and *CD44* alone and in combination. **d,** Relative mRNA expression levels of *TIMP1, MMP9* and *CD44* in D22 TPCs engineered with $NRAS^{Q61R}/TP53^{R248Q}$ overexpressing *TIMP1, MMP9* and *CD44* alone and in combination. Data are expressed as fold change over control ± SD of three independent experiments. **e,** RNAseq-based transcriptomic analysis of *TIMP1, MMP9* and *CD44* in normal (n=58) and tumor (n=58) thyroid tissue from TNMplot database. Boxes represent the interquartile range (IQR) and midline represents the median. Statistical significance was calculated using Kruskall Wallis test. **f,** Relative mRNA expression levels of *TIMP1, MMP9* and *CD44v6* in xenografts obtained by the injection of D22 TPCs engineered as indicated and compared with patient-derived PTC, FTC, and ATC. Statistical significance was calculated using the unpaired two-tailed t test. Data are expressed as mean ± SD of three independent experiments. **g,** Representative gating strategy for flow cytometry analysis of CD44v6 showed in Figure 2h. **h,** Relative mRNA expression levels of *CD44v6* in D22 TPCs engineered with the indicated mutations. Data are expressed as fold change over NTC ± SD of three independent experiments.

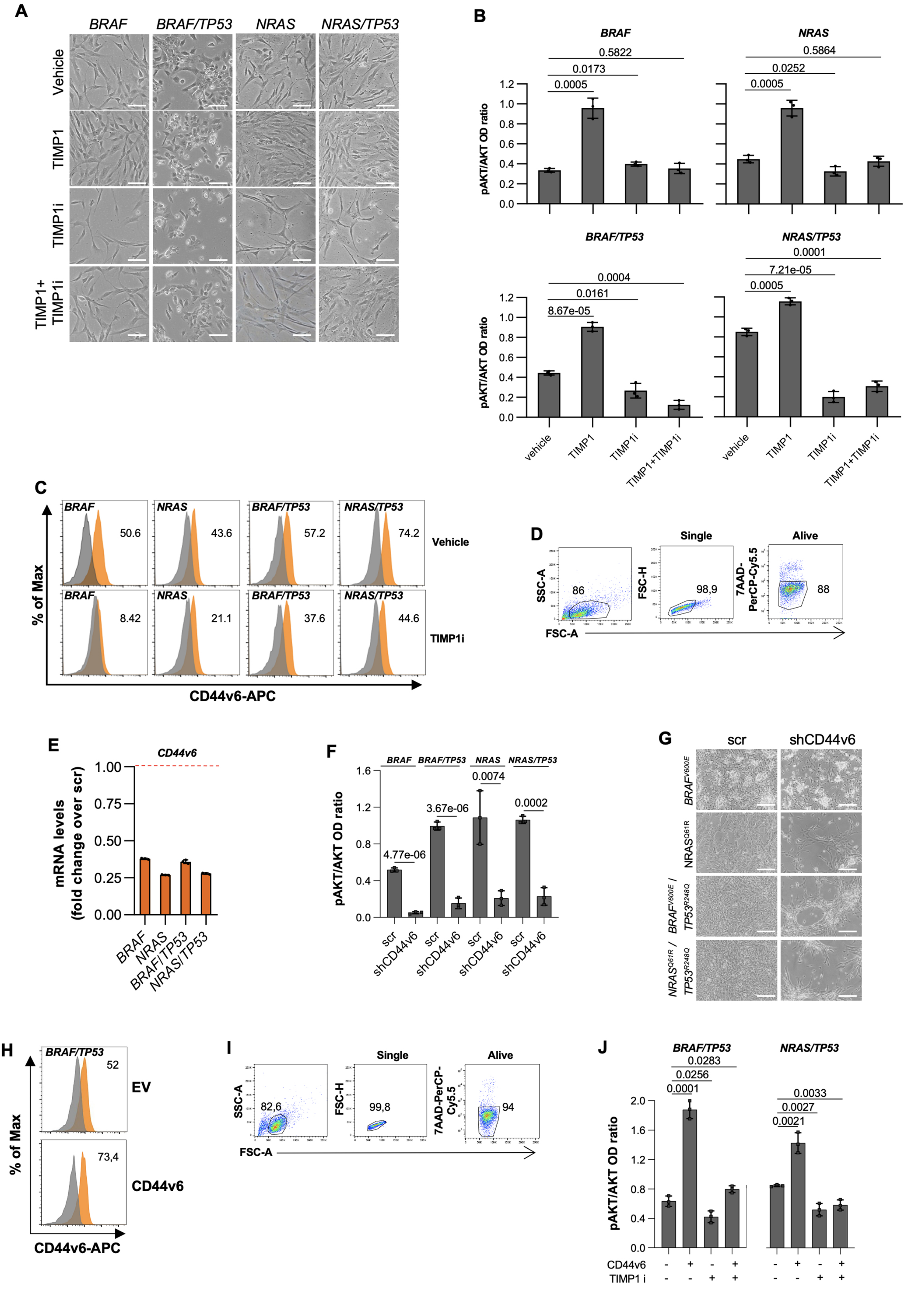

Veschi, Turdo, Modica et al. Supplementary Figure 5

Supplementary Figure 5. TIMP1 inhibition attenuates PI3K/AKT activation and is associated with CD44v6 reduced expression levels.

a, Phase contrast microscopy analysis of D22 TPCs engineered with the indicated mutations treated with vehicle, TIMP1, TIMP1 inhibitor (TIMP1i) alone or in combination for 72 hours. Scale bars, 20 µm. **b,** Optical density ratio of pAKT/AKT expression levels in D22 TPCs engineered with the indicated mutations and treated as in (a). Statistical significance was calculated using the unpaired two-tailed t test. Data are mean ± SD of three independent experiments. **c,** CD44v6 flow cytometry analysis (orange histograms) and corresponding isotype-matched control (grey histograms), in engineered D22 TPCs exposed to TIMP1i for 48 hours. **d,** Representative gating strategy for flow cytometry analysis showed in (c). **e,** mRNA levels of *CD44v6* in D22 TPCs engineered with the indicated mutations and transduced with control shRNA (scramble, scr) or CD44v6 shRNA (shCD44v6) for 72 hours. Data are expressed as fold change over scr ± SD of three independent experiments. **f,** Optical density ratio of pAKT/AKT expression levels in D22 TPCs engineered with the indicated mutations and transduced with control shRNA (scramble, scr) or CD44v6 shRNA (shCD44v6). Statistical significance was calculated using the unpaired two-tailed t test. Data are mean ± SD of three independent experiments. **g,** Phase contrast microscopy analysis of D22 TPCs engineered with the indicated mutations and transduced as in (f). Scale bars, 20 µm. **h,** CD44v6 flow cytometry analysis (orange histograms) and corresponding isotype-matched control (grey histograms), in the indicated engineered D22 TPCs transduced with empty vector (EV) or lentiviral vector expressing CD44v6 (CD44v6). **i,** Representative gating strategy for flow cytometry analysis showed in (h). **j,** Optical density ratio of pAKT/AKT expression levels in in the indicated engineered D22 TPCs overexpressing CD44v6 untreated and treated with TIMP1i for 48 hours. Statistical significance was calculated using the unpaired two-tailed t test. Data are mean ± SD of three independent experiments.

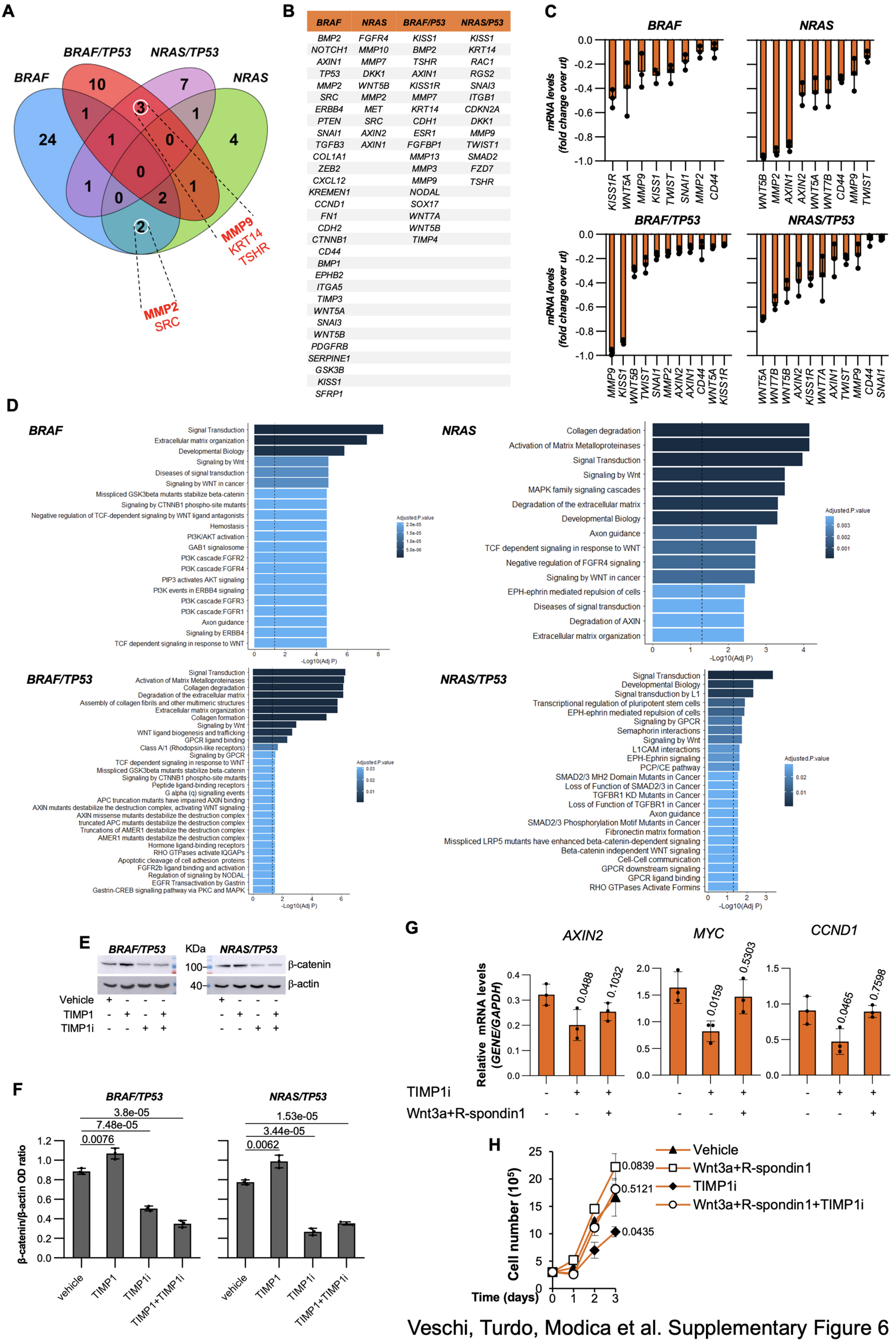

Veschi, Turdo, Modica et al. Supplementary Figure 6

Supplementary Figure 6. Targeting TIMP1 reduces TPC survival by impeding PI3K/AKT and β-catenin pathway activation.

a, Venn diagram showing common down-regulated genes (logFC < -1) in D22 TPCs engineered with the indicated mutations and treated with TIMP1 inhibitor (TIMP1i) for 24 hours. **b,** Table of down-regulated genes (logFC < -1) in D22 TPCs engineered with the indicated mutations upon TIMP1 inhibitor (TIMP1i) treatment for 24 hours. **c,** Relative mRNA expression levels of the reported genes in D22 TPCs engineered with the indicated mutations and treated with TIMP1 inhibitor (TIMP1i) for 24 hours. Data are presented as fold change over untreated (ut) ± SD of three independent experiments. **d,** Enrichment of pathway analysis of down-regulated genes (logFC < -1) in D22 TPCs engineered with the indicated mutations upon treatment with TIMP1i for 24 hours. Data are derived from Reactome database. The dashed lines indicate adjusted p-value=0.05. **e,** Immunoblot analysis of β-catenin in D22 TPCs bearing the indicated mutations and treated with vehicle, TIMP1, TIMP1 inhibitor (TIMP1i) alone or in combination for 48 hours. β-actin was used as loading control. Source data are provided as a Source Data file. **f,** Optical density ratio of β-catenin expression levels in cells engineered and treated as in (e). **g,** Relative mRNA expression levels of *AXIN, MYC* and *CCND1* in D22 TPCs engineered for $BRAF^{V600E}/TP53^{R248Q}$ mutations, untreated and treated with TIMP1 inhibitor (TIMP1i) alone or in combination with Wnt3A and R-spondin1 for 48 hours. **h,** Cell proliferation in D22 TPCs engineered as in (g) and treated with Wnt3A and R-spondin1, TIMP1 inhibitor (TIMP1i) alone or in combination with Wnt3A and R-spondin1 up to 72 hours. For (**f**–**h**) statistical significance was calculated using the two-tailed unpaired t test and data are mean ± standard error of three independent experiments.

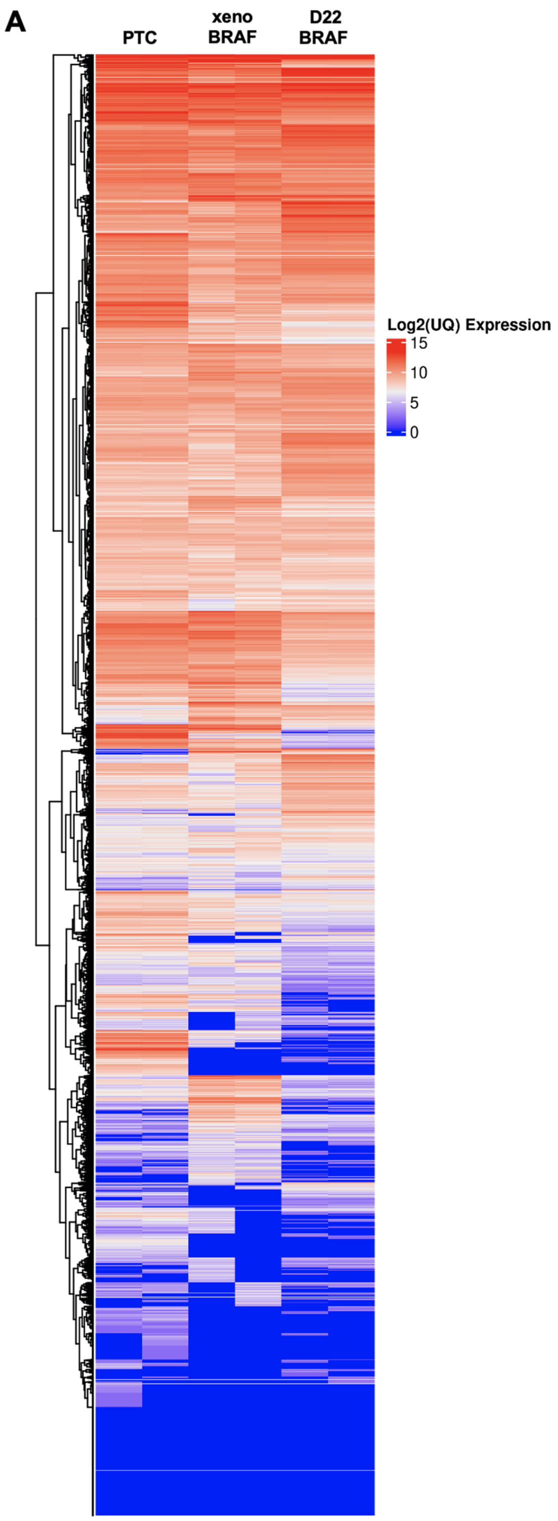

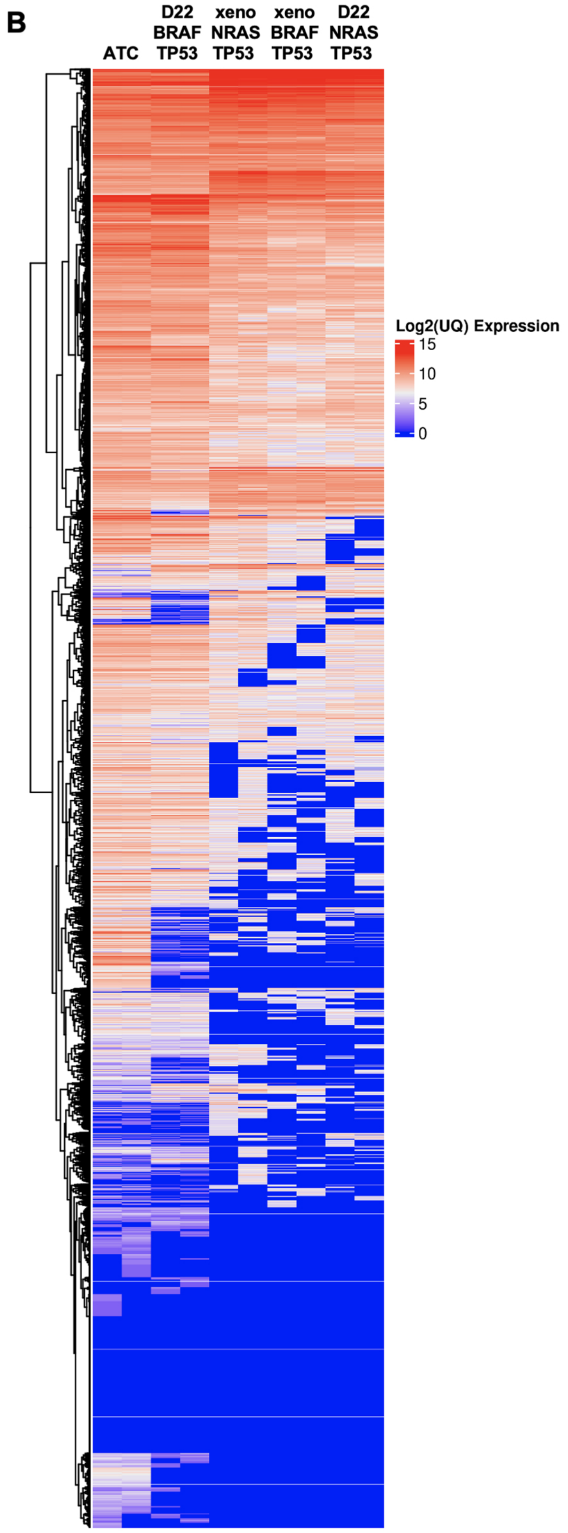

Veschi, Turdo, Modica et al. Supplementary Figure 7

Supplementary Figure 7. Total transcriptome profiles of engineered D22 TPCs, D22-derived mouse avatars and human TCs.

a and b, Clustered heatmaps showing normalized mRNA expression values of genes obtained by total transcriptome analysis (mRNAseq) in the indicated engineered D22 TPCs, engineered D22-derived xenograft tumors (xeno) and human PTC (a) and ATC (b). Mutational status of human tissues: PTC *BRAF* mutated ID#6, and ATC *BRAF/TP53* mutated ID#96. Values are expressed as Log2 upper quartile (UQ).

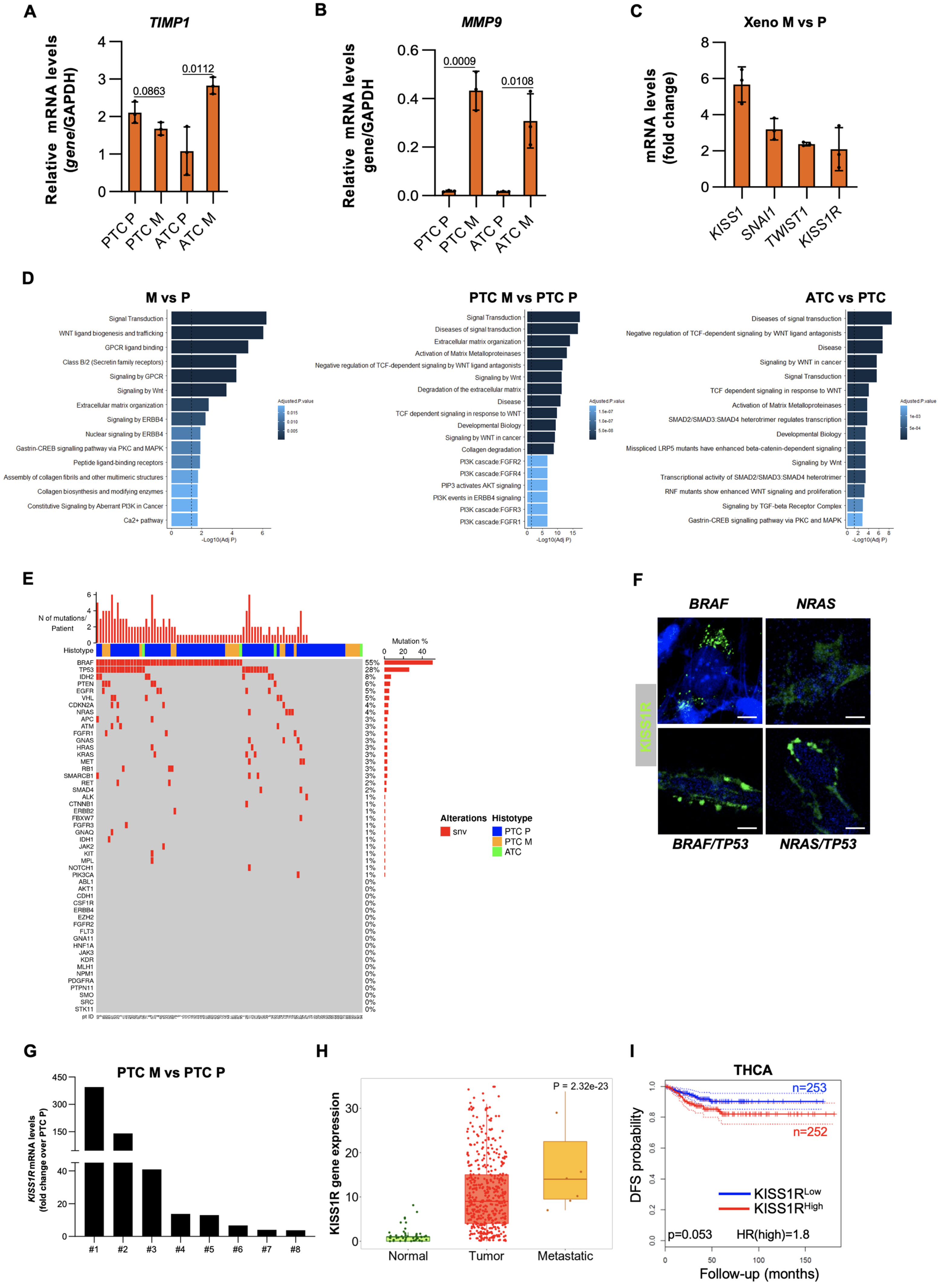

Veschi, Turdo, Modica et al. Supplementary Figure 8

Supplementary Figure 8. KISS1R expression correlates with TC aggressive phenotypes. **a and b,** Relative mRNA expression levels of *TIMP1* (a) and *MMP9* (b) in primary (P) and metastatic (M) PTC and ATC patient-derived tumors. Mutational status of primary and metastatic human tissues: PTC *BRAF/TP53* mutated ID#61, and ATC *BRAF/TP53* mutated ID#96. Statistical significance was calculated using the unpaired two-tailed t test. Data are expressed as mean ± SD of three independent experiments. **c,** Relative mRNA expression levels of the indicated genes in metastasis (M) *versus* primary (P) tumor xenografts. Data are presented as fold change of M vs P ± SD of three independent experiments. **d,** Enrichment pathway analysis of up-regulated genes (logFC > 3.5) in metastasis (M) *versus* primary (P) tumor xenografts, in PTC-derived metastasis (M) *versus* primary tumors (PTC P) and in ATC primary *versus* PTC primary tumors. Mutational status of primary and metastatic human tissues: PTC *BRAF/TP53* mutated ID#61, and ATC *BRAF/TP53* mutated ID#96. Data are derived from Reactome database. The dashed lines indicate adjusted p-value=0.05. **e,** Tile plot showing the single nucleotide variants (SNV) and the different histotypes (PTC P, PTC M and ATC) of 93 TC patients. Number of mutations for patient and mutation rate (number of patients mutated for each gene) are indicated in the top and right barplots, respectively. **f,** Immunofluorescence analysis of KISS1R in D22 TPCs engineered with the indicated mutations. Nuclei were counterstained by Toto-3. Scale bars, 5 µm. One representative of three independent experiments is shown. **g,** Relative mRNA expression levels (copies/µl) of KISS1R in PTC-derived metastasis (PTC M) vs primary tumors (PTC P). Mutational status of primary and metastatic human tissues: PTC *BRAF/TP53* mutated (#1-4) or *BRAF* mutated (#5-8). **h,** RNAseq-based transcriptomic analysis of *KISS1R* in normal (n=58), tumor (n=502) and metastatic (n=8) thyroid tissue from TNMplot. Boxes represent the IQR and midline represents the median. Statistical significance was calculated using Kruskall Wallis test. **i,** Disease free survival (DFS) analysis of KISS1R expression in thyroid cancer patients from Gepia database (thyroid cancer, THCA).

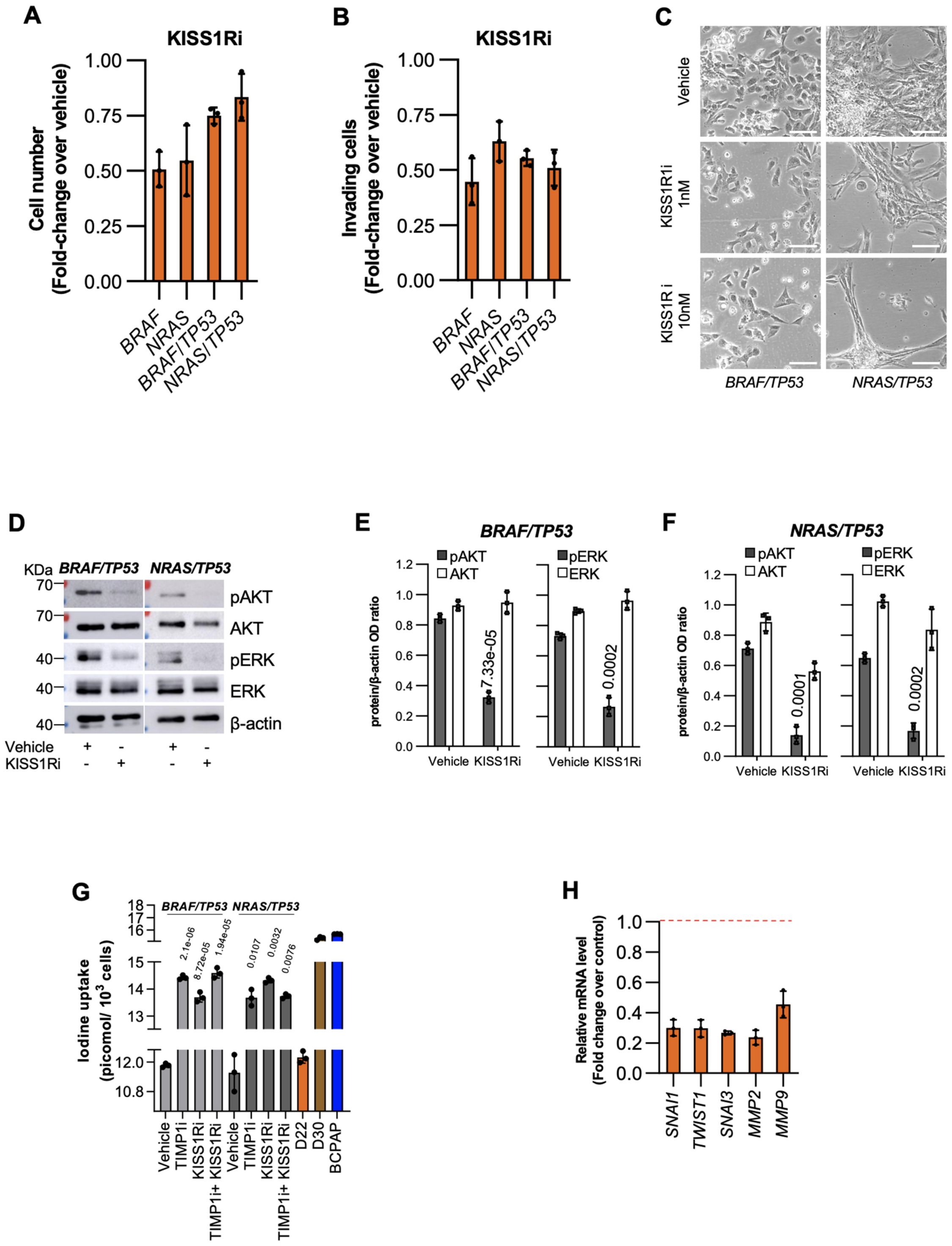
A
KISS1Ri
Cell number (Fold-change over vehicle)
BRAF
NRAS
BRAF/TP53
NRAS/TP53
B
KISS1Ri
Invading cells (Fold-change over vehicle)
C
Vehicle
KISS1R1i 1nM
KISS1R i 10nM
BRAF/TP53
NRAS/TP53
D
KDa
BRAF/TP53
NRAS/TP53
pAKT
AKT
pERK
ERK
β-actin
Vehicle
KISS1Ri
E
BRAF/TP53
pAKT
AKT
pERK
ERK
protein/β-actin OD ratio
7.33e-05
0.0002
Vehicle
KISS1Ri
F
NRAS/TP53
0.0001
0.0002
G
Iodine uptake (picomol/ 10^3 cells)
2.1e-06
8.72e-05
1.94e-05
0.0107
0.0032
0.0076
Vehicle
TIMP1i
KISS1Ri
TIMP1+ KISS1Ri
D22
D30
BCPAP
H
Relative mRNA level (Fold change over control)
SNAI1
TWIST1
SNAI3
MMP2
MMP9

Supplementary Figure 9. KISS1R targeting and TIMP1 blockade as an innovative therapeutic strategy in advanced TCs.

a and b, Cell proliferation (a) and invasion (b) analysis in D22 TPCs engineered with the indicated mutations and treated with KISS1R inhibitor (KISS1Ri), at 72 hours. Data are expressed as fold change over vehicle ± SD of three independent experiments. **c,** Phase contrast microscopy analysis of D22 TPCs engineered with the indicated mutations treated with vehicle and KISS1R inhibitor (KISS1Ri) at the indicated doses for 48 hours. Scale bars, 20 µm. One representative of three independent experiments is shown. **d,** Immunoblot analysis of pAKT, AKT, pERK and ERK in D22 TPCs bearing the indicated mutations and treated with KISS1R inhibitor (KISS1Ri). β-actin was used as loading control. One representative of three independent experiments is shown. Source data are provided as a Source Data file. **e and f,** Optical density ratio of pAKT, AKT, pERK and ERK expression levels cells engineered and treated as in (d). **g,** Iodide uptake activity in the indicated engineered D22 TPCs and treated with KISS1R inhibitor (KISS1Ri) alone or in combination with TIMP1 inhibitor (TIMP1i) for 72 hours, and compared with hESC-derived cells at D22 and D30. BCPAP cells were used a positive control. For (**e**–**g**) statistical significance was calculated using the two-tailed unpaired t test and data are mean ± standard error of three independent experiments. **h,** Relative mRNA levels of *TWIST, SNAIL, SNAI3, MMP2* and *MMP9* in D22 TPCs engineered with $NRAS^{Q61R}/TP53^{R248Q}$ mutations and transduced with MMP9 shRNA (shMMP9) for 48 hours. Data are mean ± SD of three independent experiments.

Supplementary Table 1. Sanger and NGS analyses in hESCs at D0 and engineered thyroid differentiation lineage at D6, D22, D26 and D30.

Sample	Checked Gene	Ref Seq Allele	Alt Allele Sanger	% Sanger	Alt Allele NGS	% NGS	Coding	AAChange
**hESCs at D0 (WA09)**								
D0	BRAF	T	-	0%	-	0%	c.1799T>A	p.Val600Glu
D0	TP53	G	-	0%	-	0%	c.743G>A	p.Arg248Gln
D0	NRAS	T	-	0%	-	0%	c.182A>G	p.Gln61Arg
**hESC-derived cells D6**								
D6_NTC	BRAF	T	-	-	-	-	c.1799T>A	p.Val600Glu
D6_NTC	TP53	C	-	-	-	-	c.743G>A	p.Arg248Gln
D6_NTC	NRAS	A	-	-	-	-	c.182A>G	p.Gln61Arg
D6_BRAF	BRAF	T	A	100.00%	T	99.85%	c.1799T>A	p.Val600Glu
D6_TP53	TP53	C	T	100.00%	T	99.45%	c.743G>A	p.Arg248Gln
D6_BRAFTP53	BRAF	T	A	100.00%	T	99.80%	c.1799T>A	p.Val600Glu
D6_BRAFTP53	TP53	G	A	100.00%	T	99.30%	c.743G>A	p.Arg248Gln
D6_NRAS	NRAS	A	G	100.00%	C	99.83%	c.182A>G	p.Gln61Arg
D6_NRASTP53	NRAS	A	G	100.00%	C	99.25%	c.182A>G	p.Gln61Arg
D6_NRASTP53	TP53	G	A	100.00%	T	98.90%	c.743G>A	p.Arg248Gln
**hESC-derived cells D22**								
D22_NTC	BRAF	T	-	-	-	-	c.1799T>A	p.Val600Glu
D22_NTC	TP53	G	-	-	-	-	c.743G>A	p.Arg248Gln
D22_NTC	NRAS	T	-	-	-	-	c.182A>G	p.Gln61Arg
D22_BRAF	BRAF	T	A	67.30%	T	52.33%	c.1799T>A	p.Val600Glu
D22_TP53	TP53	G	A	15.00%	T	14.90%	c.743G>A	p.Arg248Gln
D22_BRAFTP53	BRAF	T	A	95.60%	T	86.16%	c.1799T>A	p.Val600Glu
D22_BRAFTP53	TP53	G	A	93.00%	T	90.44%	c.743G>A	p.Arg248Gln
D22_NRAS	NRAS	T	C	76.60%	C	99.80%	c.182A>G	p.Gln61Arg
D22_NRASTP53	NRAS	A	G	100.00%	C	93.37%	c.182A>G	p.Gln61Arg
D22_NRASTP53	TP53	G	A	22.90%	T	33.71%	c.743G>A	p.Arg248Gln
**hESC-derived cells D26**								
D26_NTC	BRAF	T	-	-	-	-	c.1799T>A	p.Val600Glu
D26_NTC	TP53	G	-	-	-	-	c.743G>A	p.Arg248Gln
D26_NTC	NRAS	T	-	-	-	-	c.182A>G	p.Gln61Arg
D26_BRAF	BRAF	T	A	74.60%	T	57.63%	c.1799T>A	p.Val600Glu
D26_TP53	TP53	G	A	100.00%	T	94.85%	c.743G>A	p.Arg248Gln
D26_BRAFTP53	BRAF	T	A	44.10%	T	14.27%	c.1799T>A	p.Val600Glu
D26_BRAFTP53	TP53	G	A	31.87%	T	26.85%	c.743G>A	p.Arg248Gln
D26_NRAS	NRAS	A	C	100.00%	C	100.00%	c.182A>G	p.Gln61Arg
D26_NRASTP53	NRAS	T	C	35.50%	C	99.82%	c.182A>G	p.Gln61Arg
D26_NRASTP53	TP53	G	A	11.20%	T	11.50%	c.743G>A	p.Arg248Gln
**hESC-derived cells D30**								
D30_NTC	BRAF	T	-	-	-	-	c.1799T>A	p.Val600Glu
D30_NTC	TP53	G	-	-	-	-	c.743G>A	p.Arg248Gln
D30_NTC	NRAS	T	-	-	-	-	c.182A>G	p.Gln61Arg
D30_BRAF	BRAF	T	A	95.00%	T	85.37%	c.1799T>A	p.Val600Glu
D30_TP53	TP53	G	A	84.40%	T	82.49%	c.743G>A	p.Arg248Gln
D30_BRAFTP53	BRAF	T	A	77.20%	T	58.80%	c.1799T>A	p.Val600Glu
D30_BRAFTP53	TP53	G	A	4.00%	T	7.41%	c.743G>A	p.Arg248Gln
D30_NRAS	NRAS	T	C	73.90%	C	99.90%	c.182A>G	p.Gln61Arg
D30_NRASTP53	NRAS	T	C	16.90%	C	99.51%	c.182A>G	p.Gln61Arg
D30_NRASTP53	TP53	G	A	27.10%	T	31.10%	c.743G>A	p.Arg248Gln

Supplementary Table 2. Clinical data and KISS1R IHC score of the indicated TC patients.

ID	Age	Sex	Ethnicity	IHC score	Diagnosis	T	N	M
1	48	M	Caucasian	3	PTC P	3	0	0
2	54	F	Caucasian	2.5	PTC P	1	0	0
3	41	M	Caucasian	2.5	PTC P	1	0	0
4	55	F	Caucasian	3	PTC P	3	0	0
5	49	F	Caucasian	3	PTC P	1b	0	0
6	39	F	Caucasian	3	PTC P	1a	0	0
7	25	F	Caucasian	3	PTC P	1a	0	0
8	34	F	Caucasian	3	PTC P	1b	0	0
9	59	F	Caucasian	2.5	PTC P	1	0	0
10	35	F	Caucasian	2	PTC P	1	0	0
11	57	F	Caucasian	2	PTC P	2	0	0
12	33	M	Caucasian	3	PTC P	1a	0	0
13	62	M	Caucasian	3	PTC P	2	0	0
14	52	F	Caucasian	2	PTC P	1a	0	0
15	62	NA	Caucasian	3	PTC P	1	0	0
16	42	F	Caucasian	3	PTC P	1a	0	0
17	50	M	Caucasian	3	PTC P	1	0	0
18	59	F	Caucasian	3	PTC P	2	0	0
19	54	M	Caucasian	3	PTC P	1	0	0
20	44	M	Caucasian	3	PTC P	1	0	0
21	57	F	Caucasian	3	PTC P	1b	0	0
22	56	F	Caucasian	2	PTC P	1	0	0
23	58	F	Caucasian	3	PTC P	1	0	0
24	56	F	Caucasian	3	PTC P	1	0	0
25	NA	NA	Caucasian	2.5	PTC P	NA	NA	0
26	34	F	Caucasian	3	PTC P	1	0	0
27	57	F	Caucasian	3.5	PTC P	1	0	0
28	30	F	Caucasian	3.5	PTC P	1	0	0
29	42	F	Caucasian	3	PTC P	1a	0	0
30	53	F	Caucasian	3.5	PTC P	1	0	0
31	56	M	Caucasian	2	PTC P	1	0	0
32	45	F	Caucasian	3.5	PTC P	1	0	0
33	49	F	Caucasian	3	PTC P	1	0	0
34	50	M	Caucasian	3	PTC P	1	0	0
35	35	F	Caucasian	3	PTC P	1	0	0
36	41	F	Caucasian	3	PTC P	1	0	0
37	47	F	Caucasian	2.5	PTC P	1	0	0
38	47	F	Caucasian	3	PTC P	1	0	0
39	39	F	Caucasian	3	PTC P	1	0	0
40	37	F	Caucasian	3	PTC P	1	0	0
41	38	F	Caucasian	3	PTC P	1b	0	0
42	29	F	Caucasian	3	PTC P	1b	0	0
43	25	F	Caucasian	3	PTC P	1a	0	0
44	24	F	Caucasian	3	PTC P	1b	0	0
45	50	M	Caucasian	3	PTC P	2	0	0
46	61	F	Caucasian	2	PTC P	1a	0	0
47	40	F	Caucasian	2.5	PTC P	1	0	0
48	20	F	Caucasian	2.5	PTC P	1a	0	0

49	61	F	Caucasian	3	PTC P	1b	0	0
50	59	F	Caucasian	3	PTC P	1a	0	0
51	60	F	Caucasian	3	PTC P	1a	0	0
52	26	F	Caucasian	2.5	PTC P	1b	0	0
53	38	F	Caucasian	3	PTC P	2	0	0
54	31	F	Caucasian	3	PTC P	1a	0	0
55	16	F	Caucasian	2.5	PTC P	1b	0	0
56	28	M	Caucasian	2	PTC P	3a	0	0
57	34	F	Caucasian	2.5	PTC P	3	1	0
58	29	F	Caucasian	2.5	PTC P	3	1	0
59	40	M	Caucasian	2	PTC P	1	1	0
60	42	M	Caucasian	2.5	PTC P	1	1	0
61	34	F	Caucasian	2.5	PTC P	1b	1a	0
62	43	F	Caucasian	2	PTC P	1b	1	0
63	37	F	Caucasian	2	PTC P	1	1	0
64	40	F	Caucasian	2	PTC P	1a	1	0
65	27	F	Caucasian	1.5	PTC P	1	1a	0
66	30	M	Caucasian	2.5	PTC P	3	1	0
67	25	F	Caucasian	3	PTC P	1b	1	0
68	50	M	Caucasian	2.5	PTC P	1	1	0
69	35	F	Caucasian	3	PTC P	2	1a	0
70	24	F	Caucasian	2.5	PTC P	1b	1	0
71	34	F	Caucasian	1.5	PTC P	1b	1	0
72	50	F	Caucasian	1.5	PTC P	1b	1	0
73	31	F	Caucasian	3	PTC P	1a	1b	0
74	34	F	Caucasian	2	PTC M	3	1	0
75	29	F	Caucasian	2	PTC M	3	1	0
76	40	M	Caucasian	4	PTC M	1	1	0
77	42	M	Caucasian	4	PTC M	1	1	0
78	34	F	Caucasian	4	PTC M	1b	1a	0
79	43	F	Caucasian	4	PTC M	1b	1	0
80	37	F	Caucasian	3	PTC M	1	1	0
81	40	F	Caucasian	4	PTC M	1a	1	0
82	27	F	Caucasian	2	PTC M	1	1a	0
83	30	M	Caucasian	3	PTC M	3	1	0
84	25	F	Caucasian	4	PTC M	1b	1	0
85	67	M	Caucasian	2	PTC M	2	1	0
86	50	M	Caucasian	4	PTC M	1	1	0
87	35	F	Caucasian	4	PTC M	2	1a	0
88	24	F	Caucasian	3	PTC M	1b	1	0
89	34	F	Caucasian	3	PTC M	1b	1	0
90	50	F	Caucasian	3	PTC M	1b	1	0
91	50	F	Caucasian	2	PTC M	1b	1	0
92	31	F	Caucasian	3	PTC M	1a	1b	0
93	88	F	Caucasian	4	ATC	3a	0	0
94	56	F	Caucasian	3	ATC	3b	1	0
95	79	F	Caucasian	4	ATC	3b	0	0
96	90	F	Caucasian	4	ATC	4a	1b	0
97	NA	NA	Caucasian	3	ATC	NA	NA	0

*** PTC M =** locoregional lymph node metastases

Supplementary Table 3. Primer sequences for off-target analysis.

		PRIMER SEQUENCES	
	**OFF TARGETS**	Forward	Reverse
BRAF	*BRAFP1*	ATG GAT TAC TGA CAC GCC AA	ACA GAA CAA TCC CAA ATG CA
	THAP4	TGA CAA GAA GCT AGG GAA CG	TTT CAG TGC ACA CAG ATT GG
	CTNNBL1	ATG CTG CCA TGA ATT GTG AC	CTC TCA ACA CAC TAC CTC CC
NRAS	*NINL*	GTG GAG AAG GGA GTT TGT CT	AAA GGG AGG AGG GCA TAA TG
	SEMA5B	CCT CTC CCA CCT CTC AAA TC	TTT TAC AGA TGG GAA GGC CT
	SPATA5	TCA GTT AAG GCA GGA ACA GG	TGC AGT GTT TGG TTT TCT GT
TP53	*DCDC2C*	GCT TTC AAT GAC TGT GCC AT	TCA ACT CAG CAA ATC ACA TCC
	FRAS1	GAA ATG TAT TAA GCA GCA CAG AA	TCA GTG ATT TGC TTG GTT TTC
	SEMA5B	AAA TGC TTG GCC ATG TTG AG	ATG ATG TGA ACA GGA GGC AG

Supplementary Table 4. Primer sequences for qRT-PCR.

Gene	Primer Sequence
AXIN1_For	GTATGTGCAGGAGGTTATGCGG
AXIN1_Rev	CACCTTCCTCTGCGATCTTGTC
AXIN2_For	CTCCTTATCGTGTGGGCAGT
AXIN2_Rev	CTTCATCCTCTCGGATCTGC
AQP5_For	TACGGTGTGGCACCGCTCAATG
AQP5_Rev	AGTCAGTGGAGGCGAAGATGCA
CD44_For	ATGGGTTCATAGAAGGGC
CD44_Rev	AGGTGTTGGATGTGAGGA
GAPDH_For	GCTTCGCTCTCTGCTCCTCCTGT
GAPDH_Rev	TACGACCAAATCCGTTGACTCCG
KISS1_For	GGGAGCCATTAGAAAAGGTG
KISS1_Rev	TTCCAGTTGTAGTTCGGCAG
KISS1R_For	TTCATGTGCAAGTTCGTCAA
KISS1R_Rev	CAGGTTGTACAGTGCGAA
MMP2_For	TGGTGGGAACTCAGAAGGTG
MMP2_Rev	CCACATCTTTCCGTCACTGC
MMP9_For	ACTACTGTGCCTTTGAGTCC
MMP9_Rev	CCAGTACTTCCCATCCTTGA
MYC_For	GACCCCTTTAACTCAAGACT
MYC_Rev	AGTCCTGGATGATGATGTTT
NIS_For	GGCATCGTCATGTTTGTGTT
NIS_Rev	GAGGCATGTACTGGTCTGG
Pax8_For	CAGATCCTCACTCACCCTTC
Pax8_Rev	GAGCTAGAACTGGACACCTC
SCGB1A1_For	GCTGAAGAAGCTGGTGGACACC
SCGB1A1_Rev	GCGTGGACTCAAAGCATGGCAG
SFTPC_For	GTCCTCATCGTCGTGGTGATTG
SFTPC_Rev	AGAAGGTGGCAGTGGTAACCAG
SFTPB_For	TGCCTGGACCACCTCATCCTTG
SFTPB_Rev	GTCCTCACACTCTTGGCATAGG
SNAI1_For	TCGGAAGCCTAACTACAGCGA
SNAI1_Rev	AGATGAGCATTGGCAGCGAG
SNAI3_For	TGCACCTGCAAGATCTGTGGCA
SNAI3_Rev	AAGGTTGGAGCGGTCGGCAAAG
TG_For	TTTGCCCTTTGGTTGTTCTG
TG_Rev	AACAACCACTGAAGAGAGGG
TIMP1_For	AGGCTCTGATGGGAATGGTC
TIMP1_Rev	CTTGTGATTGGCTGAGCTGC
TPO_For	CAATTAAGGCGCCCATTTCA
TPO_Rev	TTCCTCTTCTCAGCCAACTG
TSHR_For	TCACATAGAAATTCGGAATACCAGGAA
TSHR_Rev	GAATAAACTTTGGTCAGGTCAGGG
TTF1_For	TTGTCCCTTTTTCTCAGTTTGA
TTF1_Rev	AAGAGTAGAACTCTGGCCATT
TWIST_For	GCCAGGTACATCGACTTCCTCT
TWIST_Rev	TCCATCCTCCAGACCGAGAAGG
WNT5A	TACGAGAGTGCTCGCATCCTCA
WNT5A	TGTCTTCAGGCTACATGAGCCG
WNT5B	CAAGGAATGCCAGCACCAGTTC
WNT5B	CGGCTGATGGCGTTGACCACG
WNT7A	AGGAGAAGGCTCACAAATGGGC
WNT7A	CGGCAATGATGGCGTAGGTGAA
WNT7B	AGAAGACCGTCTTCGGGCAAGA
WNT7B	AGTTGCTCAGGTTCCCTTGGCT
