# Supplementary material for: Recapitulating thyroid cancer histotypes through engineering embryonic stem cells
Source: Nat Commun. 2023 Mar 11;14:1351. doi: 10.1038/s41467-023-36922-1 (PMC10008571; doi:10.1038/s41467-023-36922-1)
Supplement: Supplementary file 2 — Reporting Summary [file 41467_2023_36922_MOESM2_ESM.pdf]

nature portfolio

Corresponding author(s): Giorgio Stassi, M.D.

Last updated by author(s): 14/12/22

Reporting Summary

Nature Portfolio wishes to improve the reproducibility of the work that we publish. This form provides structure for consistency and transparency in reporting. For further information on Nature Portfolio policies, see our Editorial Policies and the Editorial Policy Checklist.

Statistics

For all statistical analyses, confirm that the following items are present in the figure legend, table legend, main text, or Methods section.

n/a	Confirmed	
☐	☒	The exact sample size (*n*) for each experimental group/condition, given as a discrete number and unit of measurement
☐	☒	A statement on whether measurements were taken from distinct samples or whether the same sample was measured repeatedly
☐	☒	The statistical test(s) used AND whether they are one- or two-sided<br>*Only common tests should be described solely by name; describe more complex techniques in the Methods section.*
☐	☒	A description of all covariates tested
☐	☒	A description of any assumptions or corrections, such as tests of normality and adjustment for multiple comparisons
☐	☒	A full description of the statistical parameters including central tendency (e.g. means) or other basic estimates (e.g. regression coefficient) AND variation (e.g. standard deviation) or associated estimates of uncertainty (e.g. confidence intervals)
☐	☒	For null hypothesis testing, the test statistic (e.g. *F*, *t*, *r*) with confidence intervals, effect sizes, degrees of freedom and *P* value noted<br>*Give P values as exact values whenever suitable.*
☒	☐	For Bayesian analysis, information on the choice of priors and Markov chain Monte Carlo settings
☒	☐	For hierarchical and complex designs, identification of the appropriate level for tests and full reporting of outcomes
☒	☐	Estimates of effect sizes (e.g. Cohen's *d*, Pearson's *r*), indicating how they were calculated

Our web collection on statistics for biologists contains articles on many of the points above.

Software and code

Policy information about availability of computer code

Data collection	R2:Genomics Analysis and Visualization Platform (https://hgserver1.amc.nl/cgi-bin/r2/main.cgi) was used to collect gene expression data of KISS1 and KISS1R in thyroid cancer patients. This open source does not provide a version number. Data have been checked and collected on 10th February 2022, before the submission of the first version of the manuscript.
Data analysis	4Peaks Software v 1.8 (Griekspoor and Tom Groothuis, nucleobytes.com) was used to analyze the DNA electropherograms. Torrent Suite v.5.10.1 (ThermoFisher Scientific) was used to perform initial quality control. Ion Reporter v5.18.2.0 (ThermoFischer Scientific) was employed to single nucleotide variant (SNV) annotations. FlowJO v 10.0 version software was used for Flow Citometry analysis. Extreme Limiting Dilution Analysis (ELDA) 'limdil' function (http://bioinf.wehi.edu.au/software/elda/index.html) was used to evaluate the clonogenicity of thyroid progenitor cells. ELDA software represents an online tool endowed with an algorithm to compare multiple groups or treatment conditions, not characterized by a version number (last modified 24th October 2014). Image J software (Analyze Histogram) v 1.8.0_172 was used to calculate immunostaining intensity and NIS positivity. QuantaSoft Software (Bio-Rad) v 1.7.4.0917 was used to analyze data obtained from Droplet digital PCR experiments. STAR v 2.7.10a and Stringtie v 2.2.1, and R software v R-4.2.0 were used for the RNA-seq data analysis.

For manuscripts utilizing custom algorithms or software that are central to the research but not yet described in published literature, software must be made available to editors and reviewers. We strongly encourage code deposition in a community repository (e.g. GitHub). See the Nature Portfolio guidelines for submitting code & software for further information.

Data

Policy information about availability of data

All manuscripts must include a data availability statement. This statement should provide the following information, where applicable:

- Accession codes, unique identifiers, or web links for publicly available datasets
- A description of any restrictions on data availability
- For clinical datasets or third party data, please ensure that the statement adheres to our policy

All data relevant to the study are included in the article or uploaded as supplementary information. Uncropped western blots are included in the Source Data file. The data that support the findings of this study are available from the corresponding author (GS) upon reasonable request. NGS sequencing data from hESC-derived cells at different stage of thyroid differentiation lineage and from 93 TC patients, as well as total RNA-seq trancriptomic data of engineered D22 TPCs, D22-derived xenograft tumor tissues and human tissues from primary tumors and metastatic lesions of PTC and ATC patients, have been deposited in a public open-access GEO repository, BioProject ID PRJNA887246. The project information will be accessible with the following link http://www.ncbi.nlm.nih.gov/bioproject/887246. CTNNB1 expression values were obtained from the GSE33630 series in Gene Expression Omnibus (GEO). TIMP1, MMP9 and CD44 expression levels were obtained from The Cancer Genome Atlas (TCGA) thyroid carcinoma (THCA) branch. KISS1 and KISS1R expression values were obtained from R2: Genomics Analysis and Visualization Platform (https://hgserver1.amc.nl/cgi-bin/r2/main.cgi). TNMplot database was used for the analysis of thyroid cancer RNAseq-based data, for TIMP1/MMP9/CD44 and KISS1R gene expression analysis, respectively. Survival analyses were performed on data obtained from Gene Expression Profiling Interactive Analysis (GEPIA http://gepia.cancer-pku.cn/). Data about the histotypes and the mutational background of TCs derived from patients along the whole manuscript, are provided in Supplementary Data 1 and 2. Source data are provided with this paper.

Human research participants

Policy information about studies involving human research participants and Sex and Gender in Research.

Reporting on sex and gender	97 patients affected by thyroid cancer (TC) were involved in the study: 20 patients were men, 74 patients were women and for 3 patients this information was not available. Sex was determined based on self-reporting and consent has been obtained for sharing this individual-level information.
Population characteristics	All the covariate-relevant population characteristics of the human samples, including age, sex, ethnicity, diagnosis and TNM, are reported in Supplementary Table 2.
Recruitment	Fresh frozen or formalin-fixed paraffin-embedded (FFPE) tissues from 97 TC patients, including 73 PTC primary tumors (PTC P; ID#1-73), 19 loco-regional lymphnode metastases (PTC M; ID#74-92), and 5 ATCs who underwent thyroidectomy at the Mediterranean Institute of Oncology, were collected and analyzed in accordance with the ethical policy of the Mediterranean Institute of Oncology on Human Experimentation. Our recruitment was blind. All TC patients with available samples and associated clinical information who underwent thyroidectomy in the last five years at the Mediterranean Institute of Oncology were included. There are no bias in the recruitment of patients that may have impact the results.
Ethics oversight	Institutional Review Board (IRB) approval for the collection of human tissues derived from TC patients was obtained from the Ethical Committee of the Mediterranean Institute of Oncology, Catania, Italy (authorization n° 16/21 on February 9th, 2021). The study complied with all the ethical regulations for work with human participants. Written informed consents were obtained from all the patients, including consent to publish information about age and sex.

Note that full information on the approval of the study protocol must also be provided in the manuscript.

Field-specific reporting

Please select the one below that is the best fit for your research. If you are not sure, read the appropriate sections before making your selection.

☒ Life sciences ☐ Behavioural & social sciences ☐ Ecological, evolutionary & environmental sciences

For a reference copy of the document with all sections, see nature.com/documents/nr-reporting-summary-flat.pdf

Life sciences study design

All studies must disclose on these points even when the disclosure is negative.

Sample size	Sample sizes for both in vitro and in vivo studies was predetermined by using ANOVA t-test.
Data exclusions	No data were excluded from the analyses.
Replication	All the in vitro and in vivo experiments, in cells of any stage of differentiation, have been performed at the same time upon transfection. In vitro experiments were performed and replicated using cells maintained in independent cell culture conditions, following differentiation protocol or transfection, for at least one passage before performing the experiment. All the in vitro experiments were replicated three times and all the attempts at replication were successful.<br>In vivo replicates have been performed by housing the different groups of mice (same age for all the groups) in different cages at least 2

weeks before the beginning of the experiment. All the in vivo experiments were replicated three times and all the attempts at replication were successful.

Randomization

All the samples have been randomly allocated into the experimental groups.

Blinding

For all the other experiments blinding was not possible, since the investigator in charge of the experiment was responsible of culture, treatment and analysis, thus following the entire experimental setting. However, given the experiments and analyses here performed (instrument/software assisted) we strongly believe that the blinding is not relevant in this scenario.

Reporting for specific materials, systems and methods

We require information from authors about some types of materials, experimental systems and methods used in many studies. Here, indicate whether each material, system or method listed is relevant to your study. If you are not sure if a list item applies to your research, read the appropriate section before selecting a response.

Materials & experimental systems

n/a	Involved in the study
	☒ Antibodies
	☒ Eukaryotic cell lines
☒	☐ Palaeontology and archaeology
	☒ Animals and other organisms
☒	☐ Clinical data
☒	☐ Dual use research of concern

Methods

n/a	Involved in the study
☒	☐ ChIP-seq
	☒ Flow cytometry
☒	☐ MRI-based neuroimaging

Antibodies

Antibodies used

Flow cytometry analysis:
CD44v6 (2F10 APC, mouse IgG1, R&D systems, 5µl/sample), Oct3/4 (40/Oct-3 Alexa-fluor647, mouse IgG1K, BD Biosciences, 20µl/sample), Sox2 (245610 PE, mouse IgG2a BD Biosciences, 20µl/sample), Nanog (N31-355, mouse IgG1, BD Biosciences, 20µl/sample), CXCR4 (FAB170P PE, mouse IgG2a, R&D system, 1µl/sample), c-kit (YB5.B8 PE, mouse IgG1 BD Biosciences, 20µl/sample), Sox17 (P7-969 PE, mouse IgG1k, BD Biosciences, 5µl/sample), Fox A2 (N17-280 PE, mouse IgG1, BD Biosciences 5µl/sample), PAX8 (PAX8/1492 APC, mouse IGg2a, Novus 5µl/sample), TTF1 (REA1090 FITC, mouse IgG1, MACS Miltenyi Biotec, 16µl/sample), TSH-R (4C1 FITC, IgG2a Santa Cruz, 2µl/sample), TPO (203340, Anti-rabbit IgG H+L Alexa-488, Abcam, 2µl/sample), Thyroglobulin (SPM221 PE, mouse IgG1, Novus, 2µl/sample), NIS (SPM186, goat anti-mouse IgG (H+L) Alexa-488, Abcam, 5µl/sample), CD133 (W6B3C1 FITC, mouse IgG1, BD Bioscience, 20µl/sample), ABCG2 (5D3/CD338 APC, mouse IgG2b, BD Bioscience, 10µl/sample), Nestin (25/Nestin PerCP 5.5, mouse IgG1, BD Bioscience, 5µl/sample), HNF-4α (H-1, goat anti-mouse IgG (H+L) Alexa-488, Santa Cruz, 4µl/sample).

Immunohistochemistry:
Tg (EPR9730, rabbit monoclonal, Abcam, 1:100 dilution), CK19 (B170, mouse IgG1, Leica, 1:50 dilution), β-catenin (E-5, mouse IgG1,k, Santa Cruz, 1:25 dilution), NIS (SPM186, mouse IgG1,k, Abcam, 1:50 dilution), TIMP1 (M7293, mouse IgG1,k, Dako, 1:50 dilution), CD63 (MX-49.129.5, mouse IgG1,k, Santa Cruz, 1:50 dilution), MMP9 (4A3, mouse IgG1, ThermoFisher, 1:100 dilution), CD44v6 (2F10, mouse IgG1, R&D systems, 1:100 dilution), CD44 (156-3c11, mouse monoclonal, Cell Signaling, 1:50), Twist (Twist2C1a, mouse IgG1, Abcam, 1:25 dilution), Snail (63371, rabbit polyclonal, Abcam, 1:50 dilution), KISS1 (34010, rabbit polyclonal, Novus Bio, 1:200 dilution), KISS1R (TA351332, rabbit polyclonal, Origene, 1:50 dilution), TSHR (4C1, mouse IgG2a κ, Santa Cruz, 1:50 dilution), S100 (GA504, rabbit polyclonal, Dako, 1:1000 dilution), CDX2 (AMT28, mouse monoclonal IgG1, Novocastra, 1:50 dilution), Oct3/4 (C-10, mouse IgG1, Santa Cruz, 1:200), p40 (PA0163, Leica), P53 (DO-7, mouse monoclonal IgG2b, Novocastra, 1:100 dilution).

Immunofluorescence:
NIS (SPM186, mouse IgG1,k, Abcam), CK19 (B170, mouse IgG1, Leica), TSHR (4C1, mouse IgG2a κ, Santa Cruz) and KISS1R (NBP2-57942, rabbit polyclonal, Novus).

Western blot:
β-catenin (Ser33/37/Thr41, D13A1, rabbit IgG, CST, 1:500 dilution), TIMP1 (M7293, mouse IgG1,k, Dako, 1:400 dilution), CD44 (156-3C11, mouse IgG2a, CST, 1:500 dilution), phospho-AKT XP (Ser473; D9E, rabbit, IgG, CST, 1:1000 dilution), AKT (rabbit polyclonal, CST, 1:500 dilution), NIS (SPM186, mouse IgG1,k, Abcam, 1:1000 dilution), phospho-ERK 1/2 (Thr202/Tyr204; rabbit polyclonal, CST, 1:500 dilution), ERK 1/2 (137F5, rabbit IgG, CST, 1:1000 dilution).

Validation

All the antibodies have been validated following information provided by the manufacturer, and titrated using appropriate positive and negative controls, using different antibody dilutions. Titration of the antibodies has been routinely performed, at antibody arrival, and every 3 months max after storage. Specifically the validation of all the antibodies used for species and application as described by manufacturer's website is reported below.

Flow cytometry analysis:
CD44v6-APC (2F10, mouse IgG1, R&D systems, #FAB3660A, 5µl/sample, negative control: 11711, mouse IgG1 APC, #IC002A; positive control: human peripheral blood monocytes), Oct3/4-Alexa Fluor 647 (40/Oct-3, mouse IgG1k, BD Biosciences, #560307, 20µl/

sample, negative control: MOPC-31C, mouse IgG1k-Alexa Fluor 647, #566011; positive control: human embryonic stem cells), Sox2-PE (24561, mouse IgG2a, BD Biosciences, # 560291, 20µl/sample, negative control: MOPC-173, mouse IgG2a-PE, #565363; positive control: human embryonic stem cells), Nanog-PE (N31-355, mouse IgG1k, BD Biosciences, # 560483, 20µl/sample, negative control: MOPC-21, mouse IgG1k-PE, #555749; positive control: human embryonic stem cells), CXCR4-PE (12G5, mouse IgG2a, R&D system, #FAB170P, 1µl/sample, negative control: 20102, mouse IgG2a-PE, # IC003P;positive control: human blood lymphocytes), c-kit-PE (YB5.B8, mouse IgG1k, BD Biosciences, # 561682, 20µl/sample, negative control: MOPC-21, mouse IgG1k-PE, #555749; positive control: TF-1 cell line), Sox17-PE (P7-969, mouse IgG1k, BD Biosciences, # 561591, 5µl/sample, negative control: MOPC-21, mouse IgG1k-PE, #555749; positive control: human embryonic stem cells), Fox A2-PE (N17-280, mouse IgG1k, BD Biosciences, #561589, 5µl/sample, negative control: MOPC-21, mouse IgG1k-PE, #554680; positive control: human embryonic stem cells), PAX8-APC (PAX8/1492, mouse IGg2a, Novus, # NBP2-54539APC, 5µl/sample, negative control: 20102, mouse IgG2a-APC, # IC003A;positive control: human thyroid cell line), TTF1-FITC (REA1090, human IgG1, MACS Miltenyi Biotec, # 130-118-307, 16µl/sample, negative control: REA293, human IgG1-FITC, #130-118-354; positive control: lung cell line), TSH-R-FITC (4C1, mouse IgG2a, Santa Cruz, #sc-32262 FITC, 2µl/sample, negative control: normal mouse IgG2a-FITC, #sc-2856; positive control: HeLa cell line), TPO (rabbit polyclonal, Abcam, #203340, 2µl/sample, positive control: thyroid cell line), Thyroglobulin-PE (SPM221, mouse IgG1k, Novus Bio, # NBP2-34748PE 2µl/sample, negative control: P3.6.2.8.1, mouse IgG1k #NBP1-43319PE; positive control: thyroid cell line), NIS (SPM186, mouse IgG1k, Abcam, #ab17795, 5µl/sample, positive control: thyroid cell line), CD133-FITC (W6B3C1, mouse IgG1k, BD Bioscience, # 567033, 20µl/sample, negative control: MOPC-21, mouse IgG1k-FITC, #554679; positive control: retinoblastoma cell line), ABCG2-APC (5D3/CD338, mouse IgG2bk, BD Bioscience, #561451, 10µl/sample, negative control: 27-35, mouse IgG2b-APC, #565381; positive control: human placenta cells), Nestin-PerCP-Cyanine 5.5 (25/Nestin, mouse IgG1k, BD Bioscience, # 561231, 5µl/sample, negative control: MOPC-21, mouse IgG1k-PerCP-Cyanine 5.5, #550795; positive control: glioma cell line), HNF-4α (H-1, mouse IgG1k, Santa Cruz, # sc-3742294, 2µl/sample, positive control: colorectal cancer cell line). After incubation with unconjugated primary antibodies, cells were incubated with anti-mouse (IgG H+L Alexa-488, Thermofisher, #A-10680, 1:200) or anti-rabbit secondary antibodies (IgG H+L Alexa-488, Thermofisher, #A-11034, 1:200). All the staining were performed in staining buffer (PBS with 0.5% BSA) and for the detection of intracellular markers, cells were fixed in 2% paraformaldehyde and permeabilized using PBS 1% Triton X-100. Dead cells were excluded using 7-AAD staining (BD Biosciences).

Immunohistochemistry:
Thyroglobulin (Tg, EPR9730, rabbit monoclonal, Abcam, # ab156008, 1:100, retrieval solution: Tris/EDTA pH 9, positive control: human thyroid gland papillary carcinoma tissue), CK19 (B170, mouse IgG1, Leica, #NCL-L-CK19, 1:50, retrieval solution: Tris/EDTA pH9, positive control: human skin), β-catenin (E-5, mouse IgG1k, Santa Cruz, # sc-7963, 1:25, retrieval solution: 10 mM sodium citrate pH6; positive control: human rectum), NIS (SPM186, mouse IgG1k, Abcam, #ab17795, 1:50, retrieval solution: 10 mM sodium citrate pH6; positive control: human thyroid), TIMP1 (VT7, mouse IgG1k, Dako, #M7293, 1:50, retrieval solution: 10 mM sodium citrate pH6, positive control: colorectal cancer tissue), CD63 (MX-49.129.5, mouse IgG1k, Santa Cruz, # sc-5275, 1:50, retrieval solution: 10 mM sodium citrate pH6, positive control: human stomach), MMP9 (4A3, mouse IgG1, ThermoFisher, #X2057M, 1:100, retrieval solution: 10 mM sodium citrate pH6, positive control: esophageal adenocarcinoma), CD44v6 (2F10, mouse IgG1, R&D systems, #BBA13, 1:100, retrieval solution: 10 mM sodium citrate pH6, positive control: human colorectal adenocarcinoma), CD44 (156-3c11, mouse IgG2a, Cell Signaling, #3570, 1:50, retrieval solution: 10 mM sodium citrate pH6, positive control: human tonsil), Twist (Twist2C1a, mouse IgG1, Abcam, # ab50887, 1:25, retrieval solution: 10 mM sodium citrate pH6, positive control: epithelial ovarian carcinoma), Snail (rabbit polyclonal, Abcam,#ab63371, 1:50, retrieval solution: 10 mM sodium citrate pH6, positive control: human testis), KISS1 (rabbit polyclonal, Novus Bio, #NBP2-34010, 1:200, retrieval solution: 10 mM sodium citrate pH6, positive control: human placenta), KISS1R (rabbit polyclonal, Origene, #TA351332, 1:50, retrieval solution: 10 mM sodium citrate pH6, positive control: human ovarian cancer), TSHR (4C1, mouse IgG2ak, Santa Cruz, #sc-32262, 1:50, retrieval solution: Tris/EDTA pH9, positive control: human thyroid), S100 (rabbit polyclonal, Dako, #GA504, 1:100, retrieval solution: 10 mM sodium citrate pH6, positive control: breast carcinoma), CDX2 (AMT28, mouse IgG1k, Novus Bio, #NB100-699, 1:50, retrieval solution: 1 mM EDTA buffer pH 8.0, positive control: human colon), Oct3/4 (C-10, mouse IgG1, Santa Cruz, # sc-5279, 1:200, retrieval solution: 10 mM sodium citrate pH6, positive control: human adrenal gland), p40 (BC28, mouse IgG1, Leica, #PA0163, retrieval solution: Tris/EDTA pH9, positive control: bladder carcinoma), P53 (DO-7, mouse IgG2b, Novocastra, #NCL-L-p53-DO7, 1:100, retrieval solution: 10 mM sodium citrate pH6, positive control: colorectal carcinoma).

Immunofluorescence:
NIS (SPM186, mouse IgG1k, Abcam, #ab17795, 1:50, positive control: MCF7 cell line), CK19 (B170, mouse IgG1, Leica, #NCL-L-CK19, 1:50, positive control: TPC-1 cell line), TSHR (4C1, mouse IgG2ak, Santa Cruz, #sc-32262, 1:100, positive control: A431 cell line) and KISS1R (rabbit polyclonal, Novus Bio, #NBP2-57942, 1:100, positive control: HepG2 cell line). Primary antibodies were revealed by using anti-mouse (IgG H+L Alexa-488, Thermofisher, #A-10680, 1:200) or anti-rabbit secondary antibodies (IgG H+L Alexa-488, Thermofisher, #A-11034, 1:200), and nuclei were counterstained using Toto-3 iodide (Thermofisher).

Western blot:
Non-phospho (active) β-catenin (Ser33/37/Thr41, D13A1, rabbit IgG, CST, #8814, 1:500 in 5% w/v BSA, 1X TBS, 0.1% Tween 20, positive control: HeLa cell line), TIMP1 (VT7, mouse IgG1k, Dako, #M7293, 1:400 dilution in 5% w/v BSA, 1X TBS, 0.1% Tween 20, positive control: MCF-7 cell line), CD44 (156-3C11, mouse IgG2a, CST, #3570, 1:500 dilution in 5% w/v nonfat dry milk, 1X TBS, 0.1% Tween 20, positive control: HeLa cell line), phospho-AKT XP (Ser473; D9E, rabbit, IgG, CST, #4060, 1:1000 in 5% w/v BSA, 1X TBS, 0.1% Tween 20, positive control: NIH/3T3 cell line), AKT (rabbit polyclonal, CST, #9272, 1:500 dilution in 5% w/v BSA, 1X TBS, 0.1% Tween 20, positive control: NIH/3T3 cell line), NIS (SPM186, mouse IgG1k, Abcam, #ab17795, 1:1000 in 5% w/v BSA, 1X TBS, 0.1% Tween 20, positive control: breast normal tissue lysate), phospho-ERK 1/2 (Thr202/Tyr204; rabbit polyclonal, CST,#9101, 1:500 dilution in 5% w/v BSA, 1X TBS, 0.1% Tween 20, positive control: MEFs treated with bFGF 100ng/mL for 30 minutes), ERK 1/2 (137F5, rabbit IgG, CST,#4695, 1:1000 in 5% w/v BSA, 1X TBS, 0.1% Tween 20, positive control: HeLa cell line), β-actin (8H10D10, mouse IgG2b, CST, #3700, 1:1000 in 5% w/v nonfat dry milk, 1X TBS, 0.1% Tween 20, positive control: HeLa cell line). Anti-mouse (goat H+L, ThermoFisher Scientific, #31430, 1:2000) or anti-rabbit (goat H+L, ThermoFisher Scientific, #31460, 1:2000) HRP-conjugated antibodies were used to reveal primary antibodies.

Eukaryotic cell lines

Policy information about cell lines and Sex and Gender in Research

Cell line source(s)	The human embryonic stem cell (hESC) line, WA09, was purchased by Wi Cell, while BCPAP (catalog number ACC 273), TT2609co2 (catalog number ACC 510) and Cal62 (catalog number ACC 448), human papillary, follicular and anaplastic thyroid carcinoma cells, respectively, were obtained from DSMZ and cultured according to the manufacturer's instructions, as reported in Methods section. HEK-293T packaging cells (catalog number CRL-3216) were obtained from ATCC and cultured following the manufacturer's instructions.
Authentication	The cell lines were not authenticated.
Mycoplasma contamination	All cell lines used in the study have been monthly tested for mycoplasma contamination using the MycoAlertTM Mycoplasma Detection Kit (Lonza, LT07-318), and resulted negative before being used for all the here reported experiments. Cell lines resulted positive for mycoplasma contamination have been promptly discarded.
Commonly misidentified lines (See ICLAC register)	No misidentified cell lines were used in this study.

Animals and other research organisms

Policy information about studies involving animals; ARRIVE guidelines recommended for reporting animal research, and Sex and Gender in Research

Laboratory animals	4-6 weeks old female NOD SCID mice were purchased by Charles River Laboratories and maintained in a barrier facility for animals in a temperature-controlled system characterized by 22 Celsius degrees and 50% humidity, with a 12 hours dark/light cycle within cages (Tecniplast) with radiation-sterilized bedding (SAWI Research Bedding, JELU-WERK). Mice were given ad libitum access to 0.45 µm-filtered tap water in sterile drinking bottles and to pelleted chow (Special Diets Services-811900 VRF1 (P)).
Wild animals	No wild animals were used in the study.
Reporting on sex	Our findings apply to only female sex. All the mice utilized for the in vivo experiments described in the manuscript were female. Sex was not considered in our study design.
Field-collected samples	No field collected samples were used in the study.
Ethics oversight	As reported in the manuscript, all the in vivo procedures complied with the institutional (University of Palermo) animal care committee guidelines (authorization #1281/2015-PR, Italian Ministry of Health).

Note that full information on the approval of the study protocol must also be provided in the manuscript.

Flow Cytometry

Plots

Confirm that:

- [x] The axis labels state the marker and fluorochrome used (e.g. CD4-FITC).
- [x] The axis scales are clearly visible. Include numbers along axes only for bottom left plot of group (a 'group' is an analysis of identical markers).
- [x] All plots are contour plots with outliers or pseudocolor plots.
- [x] A numerical value for number of cells or percentage (with statistics) is provided.

Methodology

Sample preparation	As reported in the Methods section of the submitted manuscript: cycle analysis of hESCs-derived cells at different stage of thyroid differentiation was analyzed by incubating cells with 1 ml of Nicoletti Buffer (0.1% of Sodium citrate, 0.1% of Triton x-100, 50 µg/mlof Propidium Iodide, 10 µg/ml of Rnase solution) in the dark at 4 °C overnight. DNA content was evaluated by BD FACS Lyric flow cytometer (BD Clinical system, BD Biosciences). For FACS analysis, hESCs-derived thyroid progenitor cells were washed in PBS and exposed for 1 hour at 4 °C to specific antibodies. Tissues from normal, PTC, FTC and ATC thyroids were digested and thyrocytes were isolated, purified and cultured as previously reported (PMID: 14583474). Following enzymatic dissociation with Accutase (A1110501, ThermoFisher), 1×10,000 cells were stained in 200 µl of staining buffer (PBS with 0.5% BSA) with specific fluorescence-conjugated antibody, or the corresponding, according to the manufacturer's recommendations. For the detection of intracellular markers, cells were fixed in 2% paraformaldehyde and permeabilized using PBS 1% Triton X-100. The dead cells were excluded using the 7-AAD (BD Biosciences).
Instrument	DNA content and marker expression was evaluated by BD FACS Lyric flow cytometer (BD Clinical system, BD Biosciences).

Software	Data were analyzed by FlowJO v10.0 software.
Cell population abundance	No cell sorting procedures have been performed in the manuscript.
Gating strategy	Before flow cytometry analysis, a gating strategy has been performed in order to exclude cell debris (SSC-A vs FSC-A), doublets (FSC-H vs FSC-A), and dead cells (7-AAD vs FSC-A).

☒ Tick this box to confirm that a figure exemplifying the gating strategy is provided in the Supplementary Information.
