# Supplementary material for: Recapitulating thyroid cancer histotypes through engineering embryonic stem cells
Source: Nat Commun. 2023 Mar 11;14:1351. doi: 10.1038/s41467-023-36922-1 (PMC10008571; doi:10.1038/s41467-023-36922-1)
Supplement: Supplementary file 3 — Description of Additional Supplementary Files [file 41467_2023_36922_MOESM3_ESM.pdf]

Description of Additional Supplementary Files

Supplementary Data 1: Clinical information and mutational background of patients included in datasets reported in Fig 1h, 2f, 5e, 5f and 5g.

Supplementary Data 2: Next Generation Sequencing data of 93 thyroid cancer patients included in the study.
