# Supplementary material for: Recapitulating thyroid cancer histotypes through engineering embryonic stem cells
Source: Nat Commun. 2023 Mar 11;14:1351. doi: 10.1038/s41467-023-36922-1 (PMC10008571; doi:10.1038/s41467-023-36922-1)
Supplement: Supplementary file 6 — Source Data [file 41467_2023_36922_MOESM6_ESM.zip › 356656_2_supp_7180278_rmxlm8.pptx]

## Slide 1
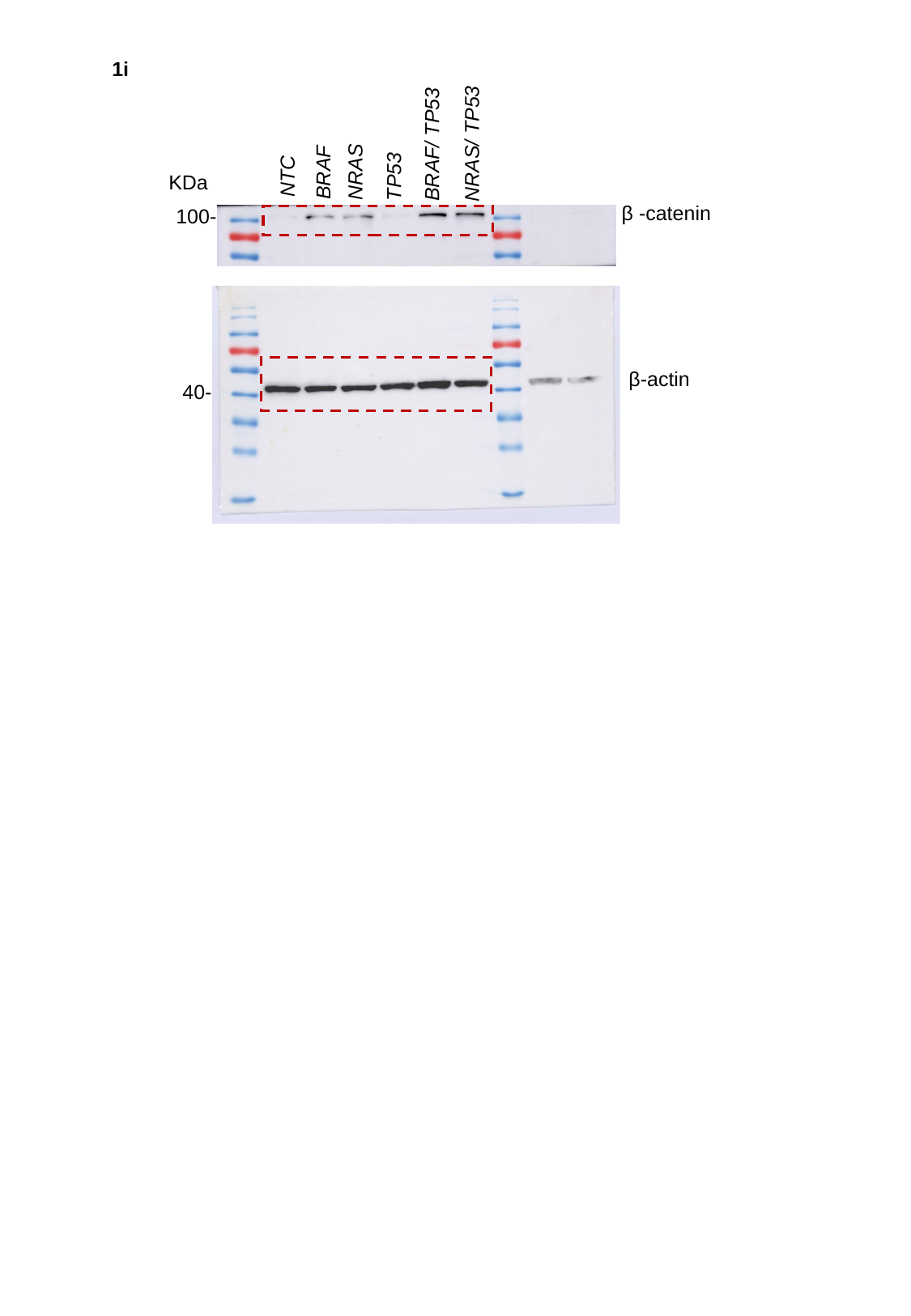

1i
NRAS/ TP53
BRAF/ TP53
NRAS
BRAF
NTC
TP53
KDa
β -catenin
100-
β-actin
40-

## Slide 2
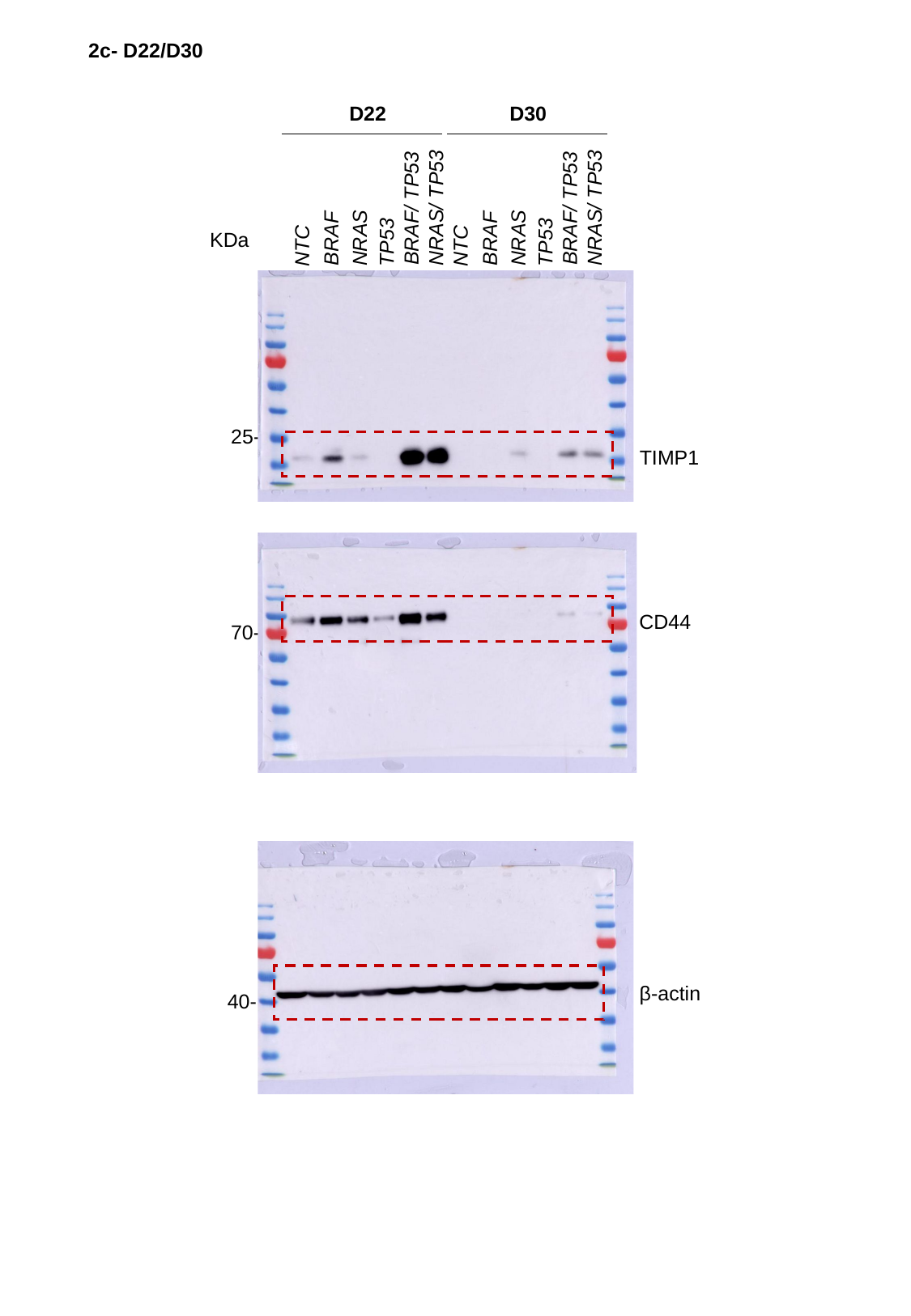

2c- D22/D30
D22
D30
NRAS/ TP53
NRAS/ TP53
BRAF/ TP53
BRAF/ TP53
NRAS
NRAS
BRAF
BRAF
KDa
TP53
TP53
NTC
NTC
25-
TIMP1
CD44
70-
β-actin
40-

## Slide 3
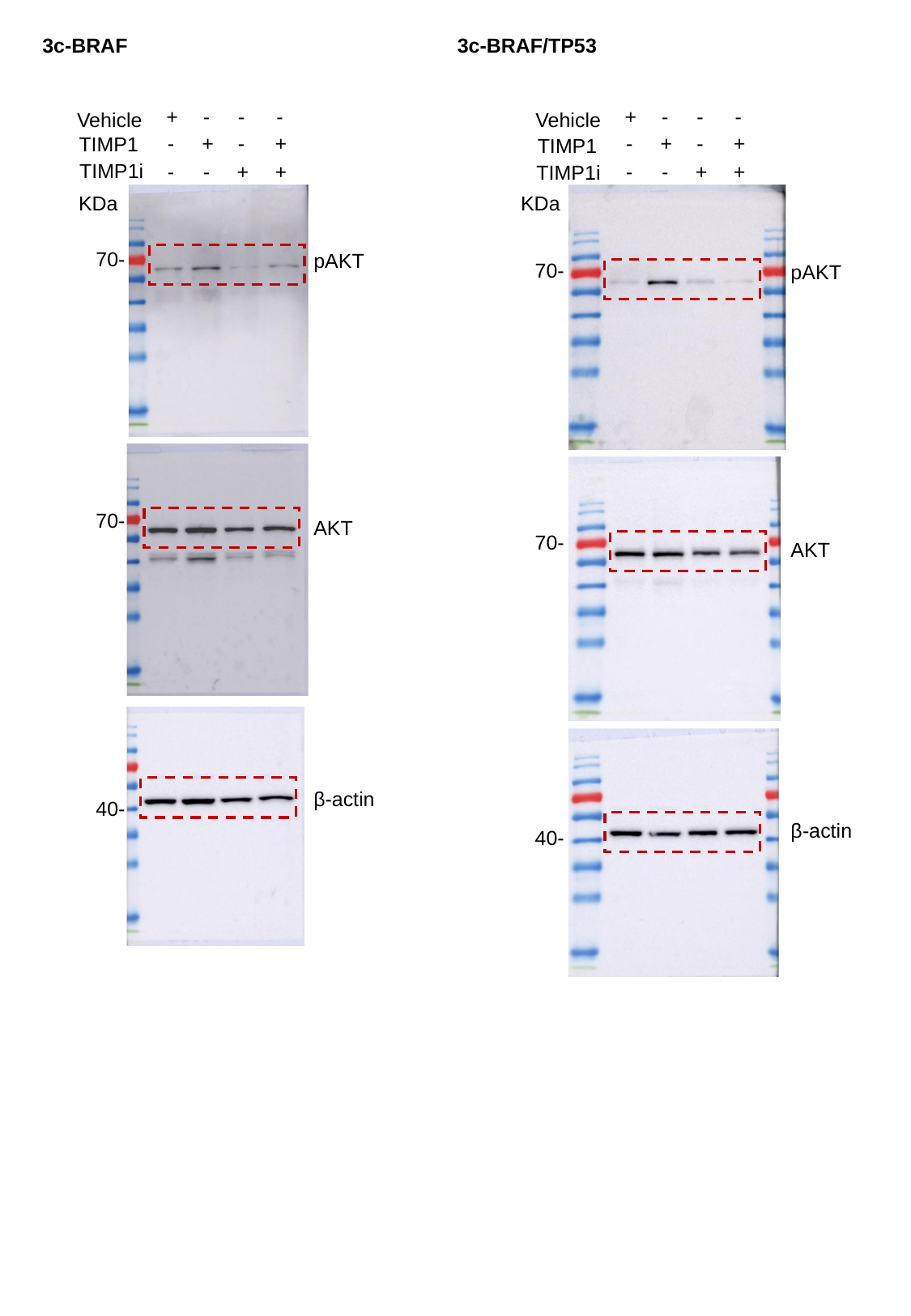

3c-BRAF
3c-BRAF/TP53
+
-
-
-
Vehicle
-
+
-
+
TIMP1
TIMP1i
-
-
+
+
+
-
-
-
Vehicle
-
+
-
+
TIMP1
-
-
+
+
TIMP1i
KDa
70-
pAKT
70-
AKT
β-actin
40-
KDa
70-
pAKT
70-
AKT
β-actin
40-

## Slide 4
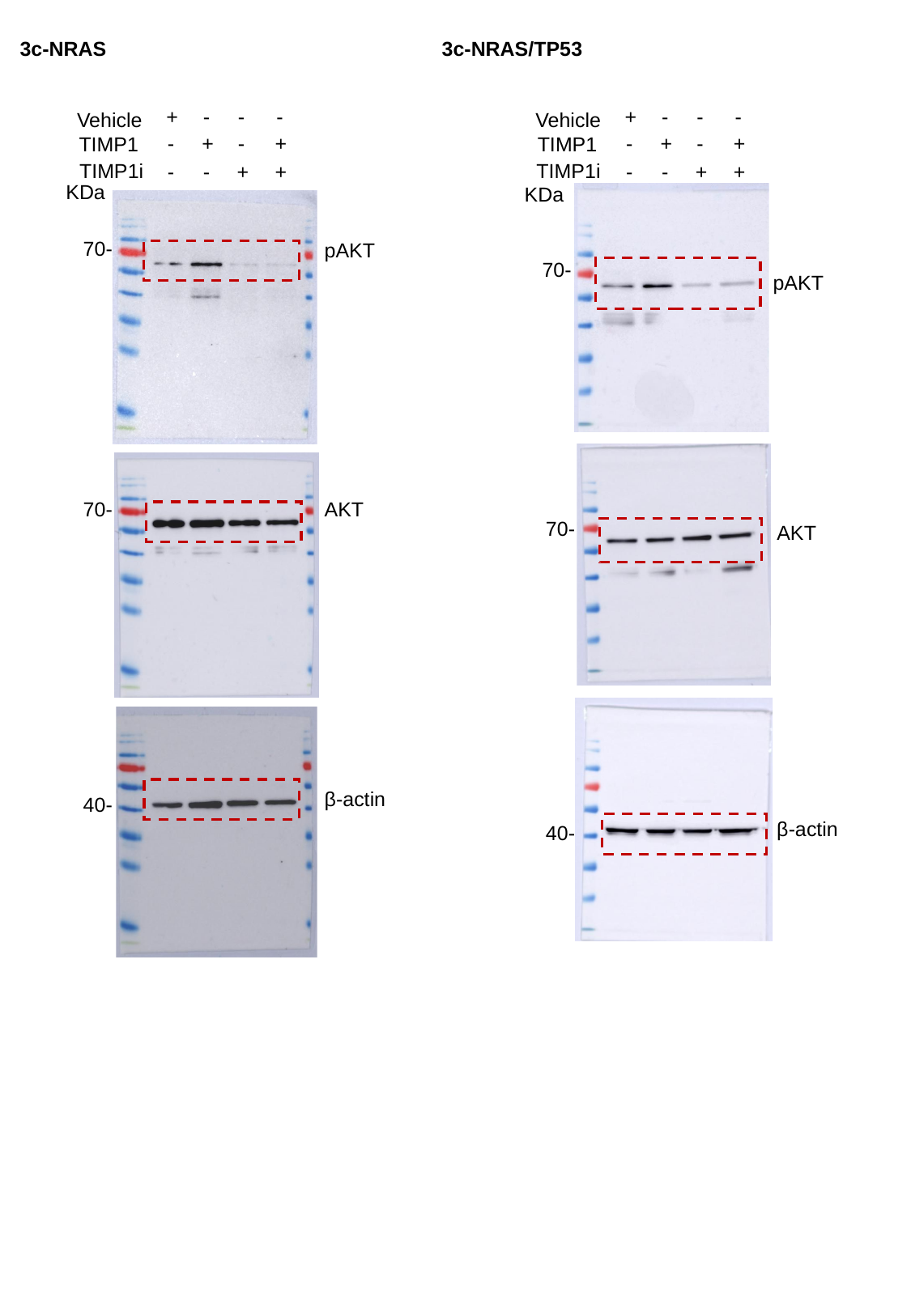

3c-NRAS
3c-NRAS/TP53
+
-
-
-
Vehicle
-
+
-
+
TIMP1
TIMP1i
-
-
+
+
KDa
70-
pAKT
70-
AKT
β-actin
40-
+
-
-
-
Vehicle
-
+
-
+
TIMP1
TIMP1i
-
-
+
+
KDa
70-
pAKT
70-
AKT
β-actin
40-

## Slide 5
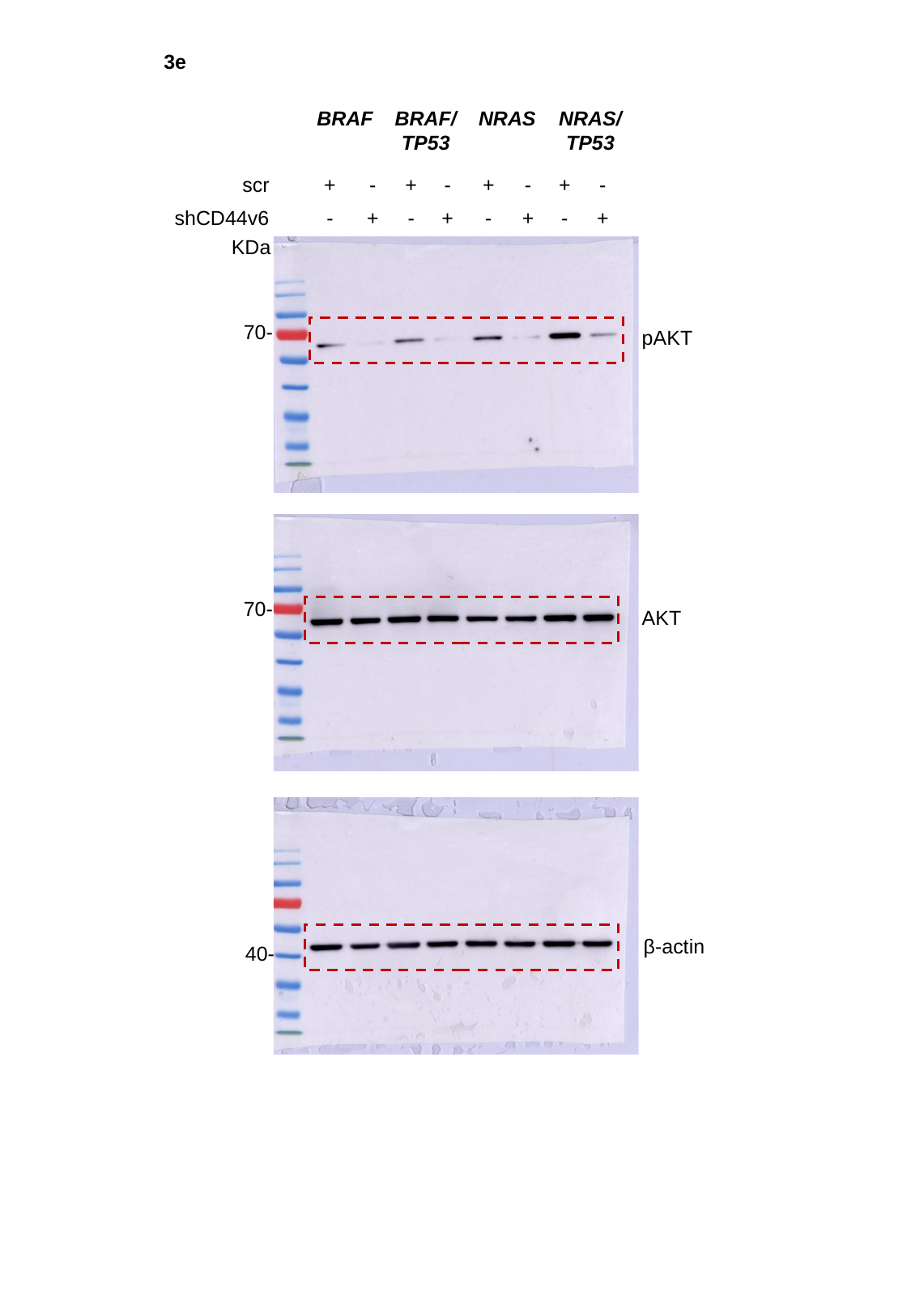

3e
BRAF/
TP53
NRAS
NRAS/
TP53
BRAF
scr
+
-
+
-
+
-
+
-
shCD44v6
-
+
-
+
-
+
-
+
KDa
70-
pAKT
70-
AKT
β-actin
40-

## Slide 6
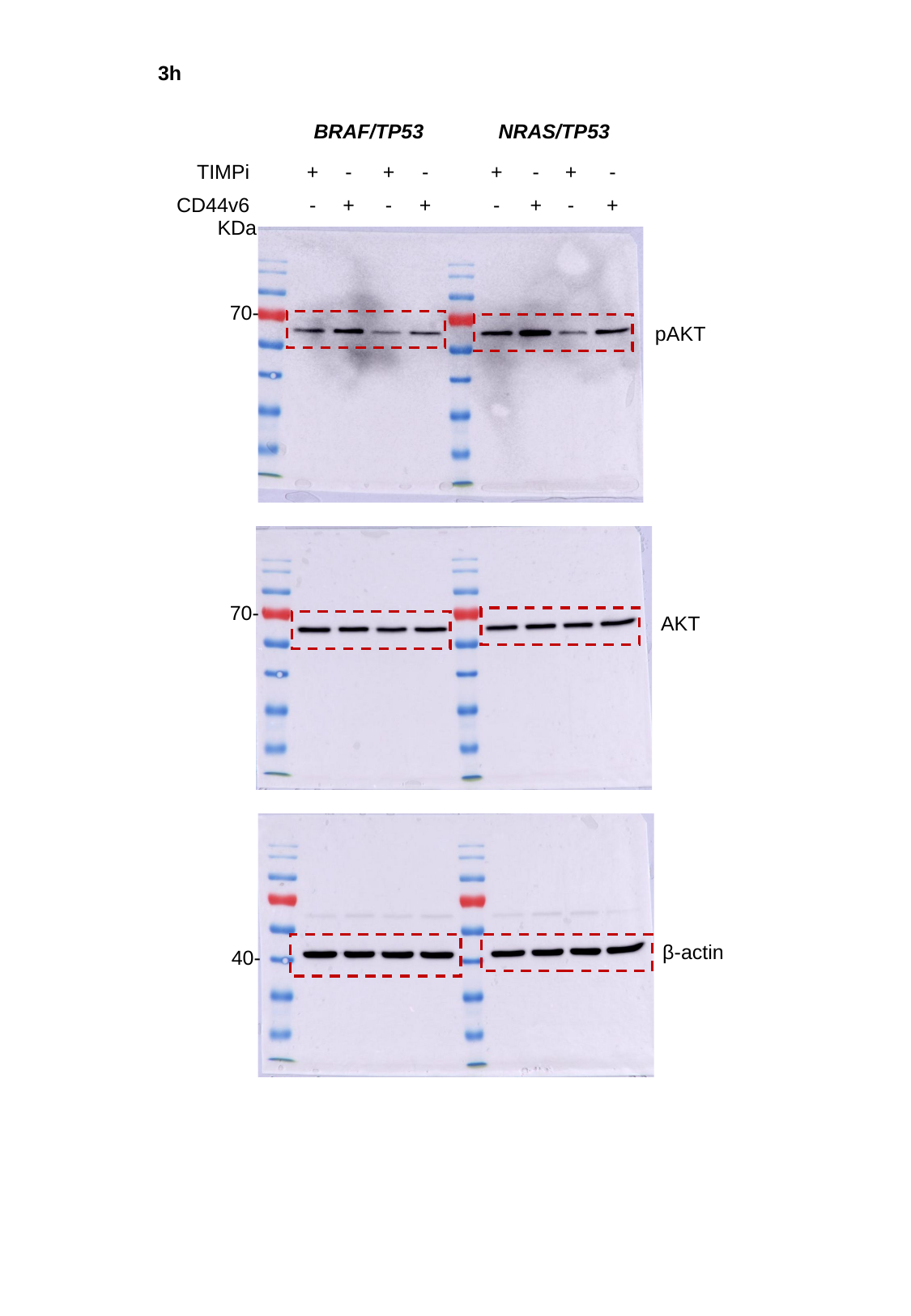

3h
BRAF/TP53
NRAS/TP53
TIMPi
+
-
+
-
+
-
+
-
CD44v6
-
+
-
+
-
+
-
+
KDa
70-
pAKT
70-
AKT
β-actin
40-

## Slide 7
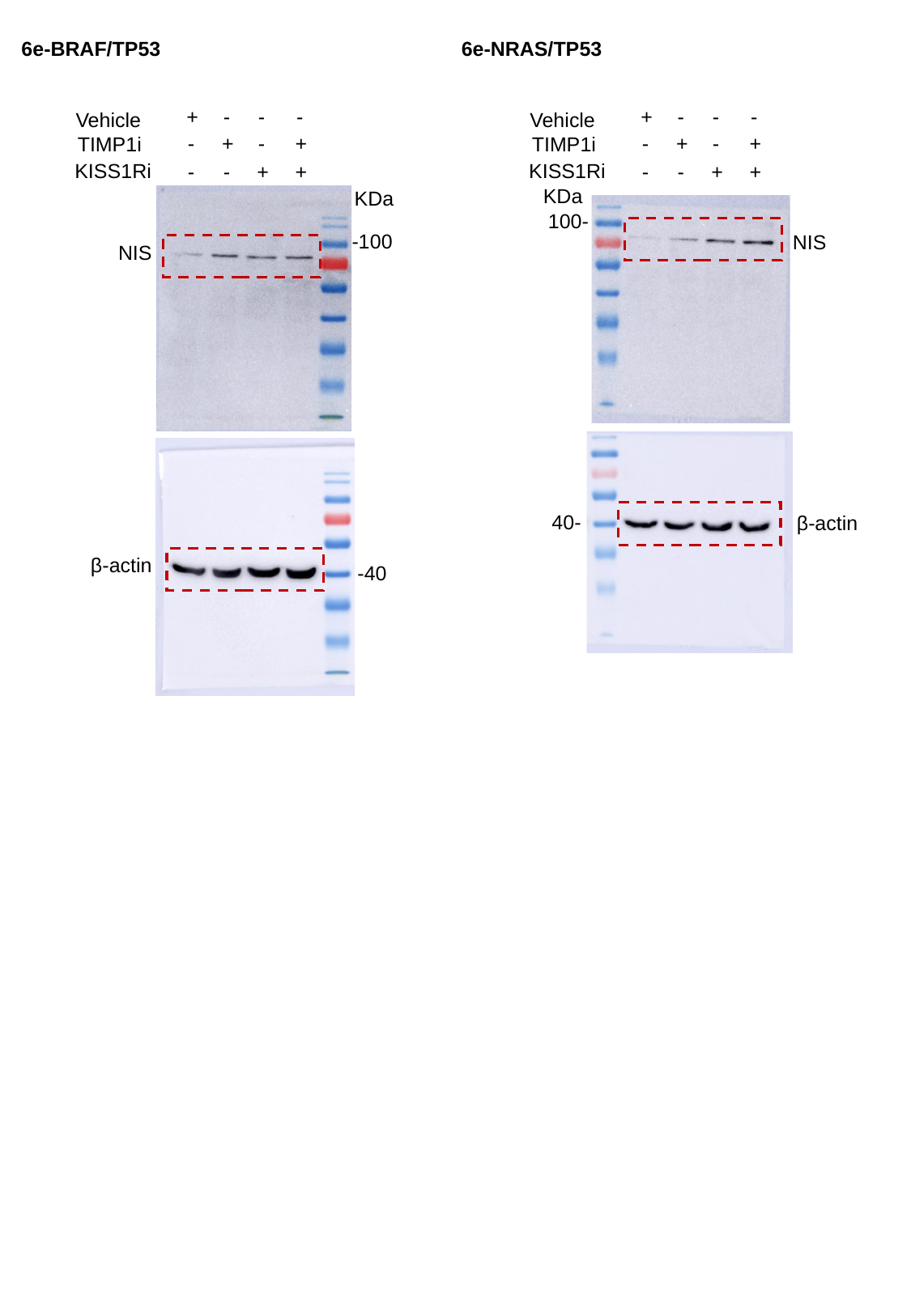

6e-BRAF/TP53
6e-NRAS/TP53
+
-
-
-
Vehicle
-
+
-
+
TIMP1i
KISS1Ri
-
-
+
+
+
-
-
-
Vehicle
-
+
-
+
TIMP1i
KISS1Ri
-
-
+
+
KDa
KDa
100-
-100
NIS
NIS
40-
β-actin
β-actin
-40

## Slide 8
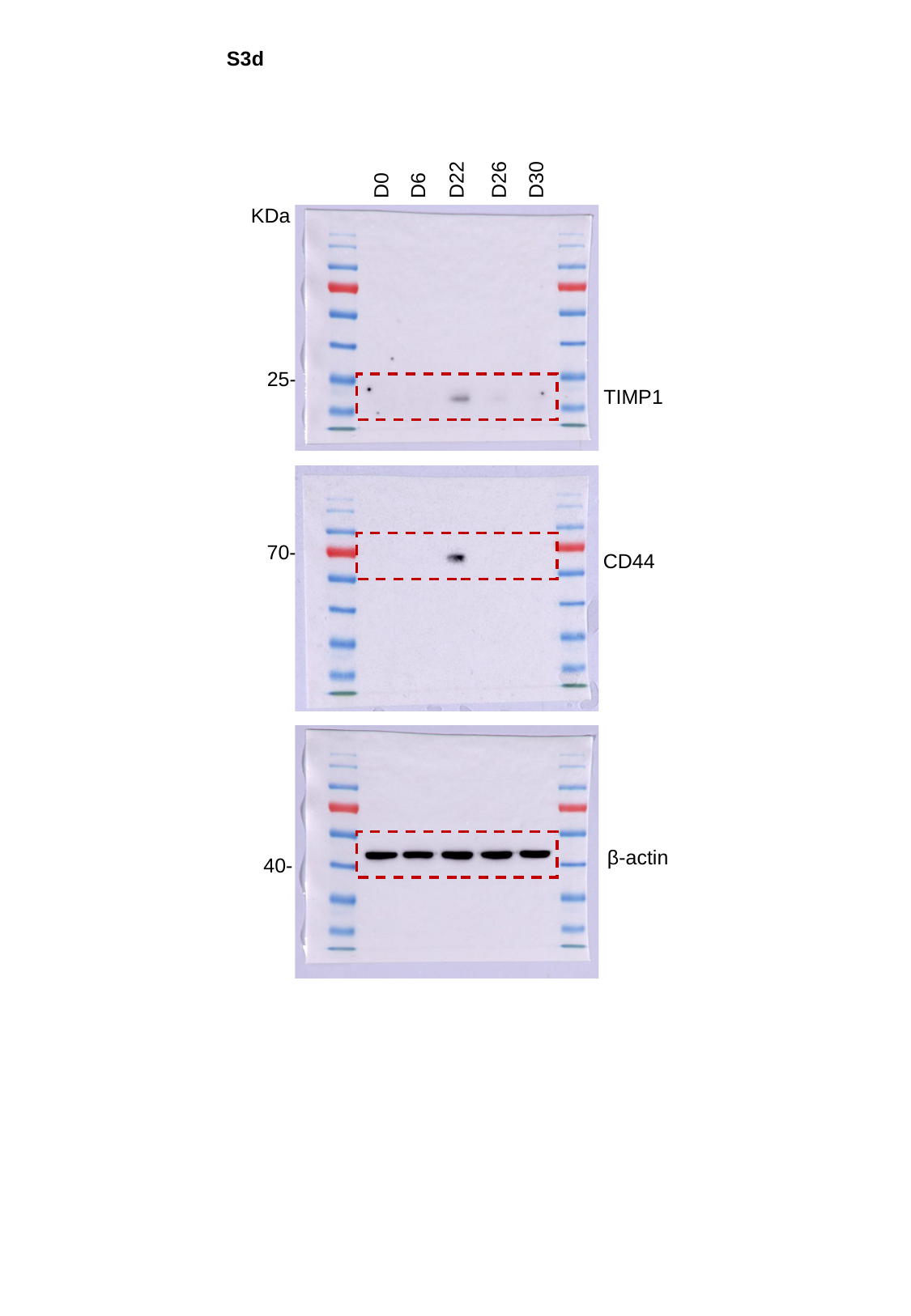

S3d
D22
D26
D30
D0
D6
KDa
25-
TIMP1
70-
CD44
β-actin
40-

## Slide 9
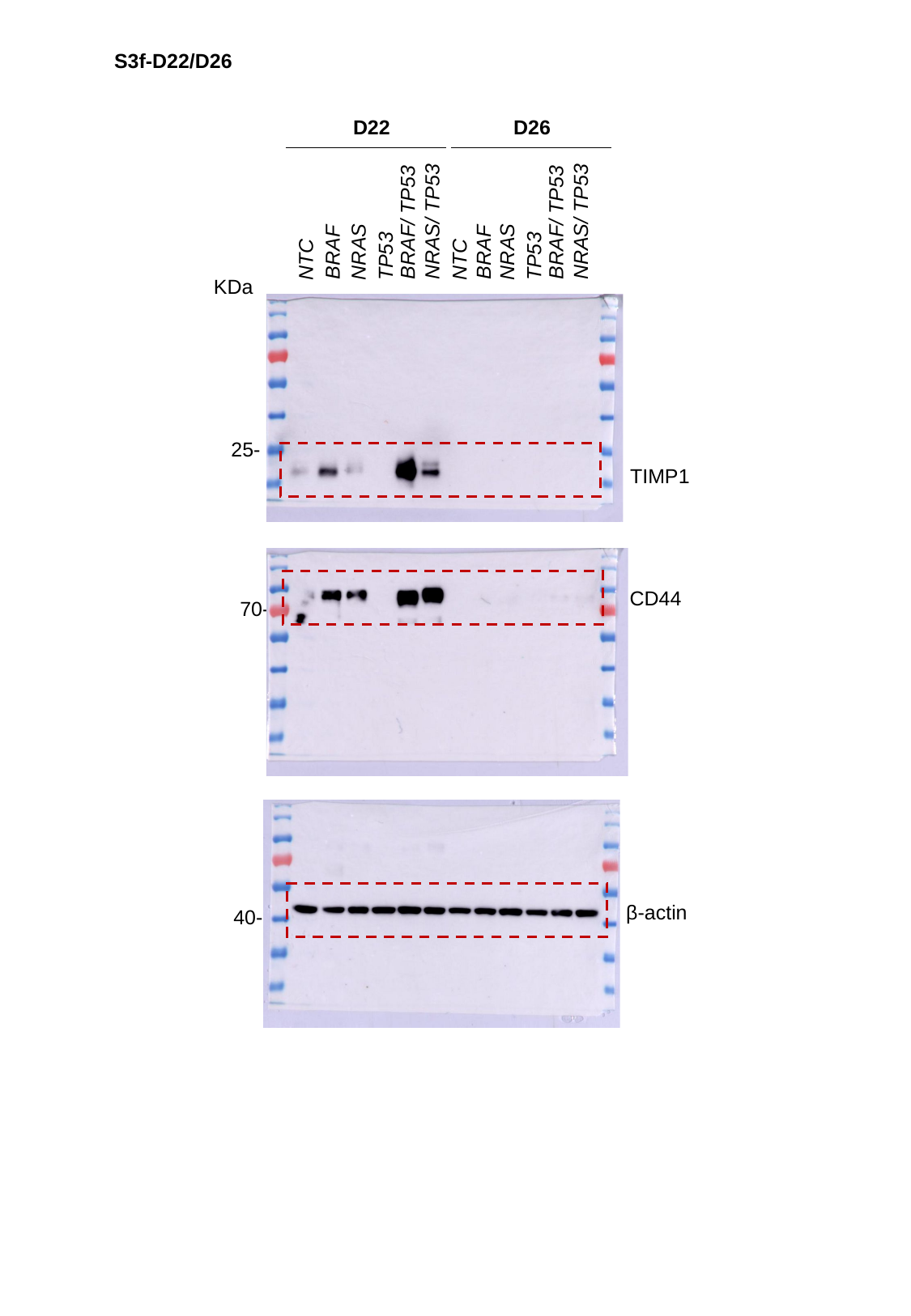

S3f-D22/D26
D22
D26
NRAS/ TP53
NRAS/ TP53
BRAF/ TP53
BRAF/ TP53
NRAS
NRAS
BRAF
BRAF
TP53
TP53
NTC
NTC
KDa
25-
TIMP1
CD44
70-
β-actin
40-

## Slide 10
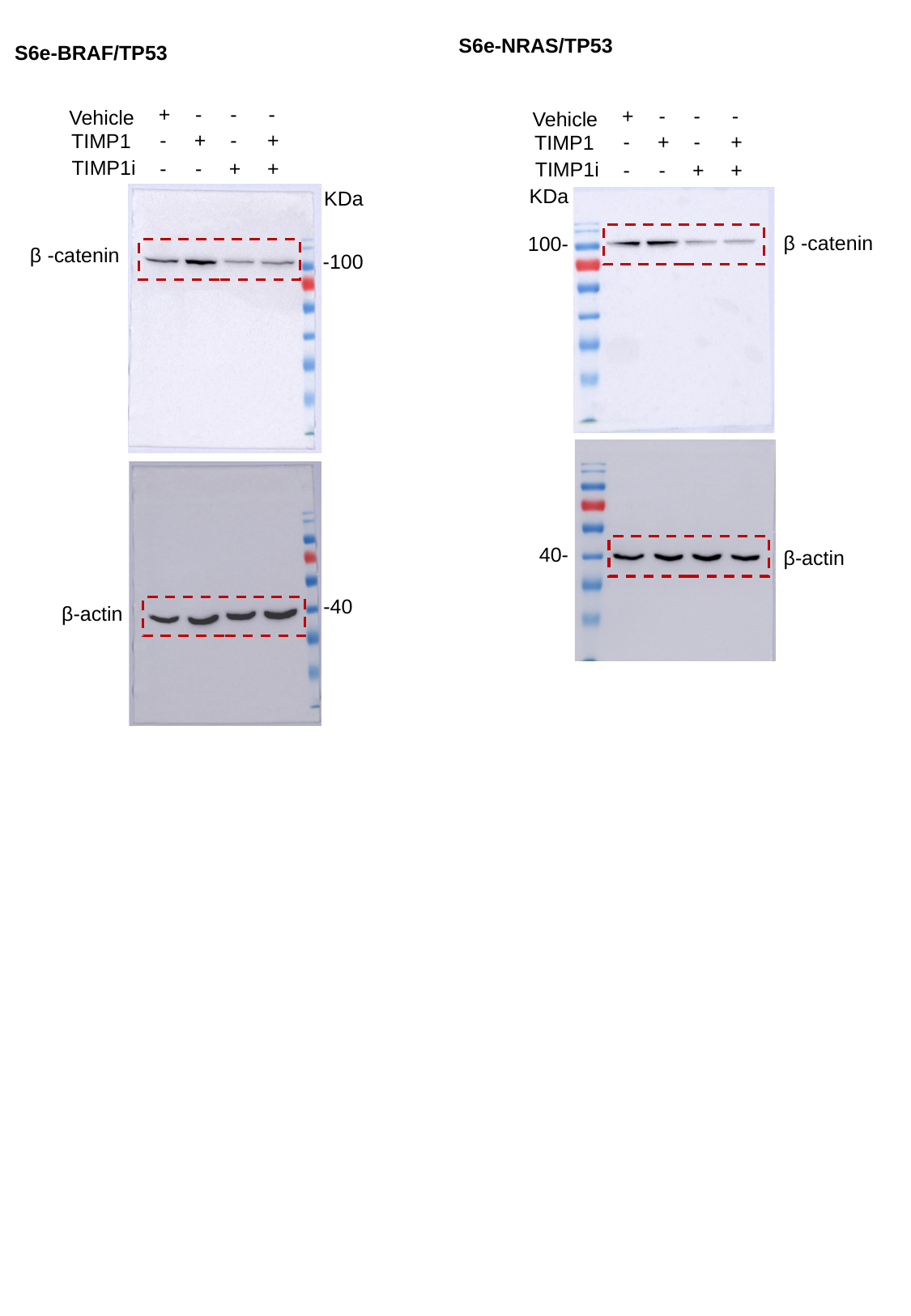

S6e-NRAS/TP53
S6e-BRAF/TP53
+
-
-
-
+
-
-
-
Vehicle
-
+
-
+
TIMP1
TIMP1i
-
-
+
+
Vehicle
-
+
-
+
TIMP1
TIMP1i
-
-
+
+
KDa
KDa
β -catenin
100-
β -catenin
-100
40-
β-actin
-40
β-actin

## Slide 11
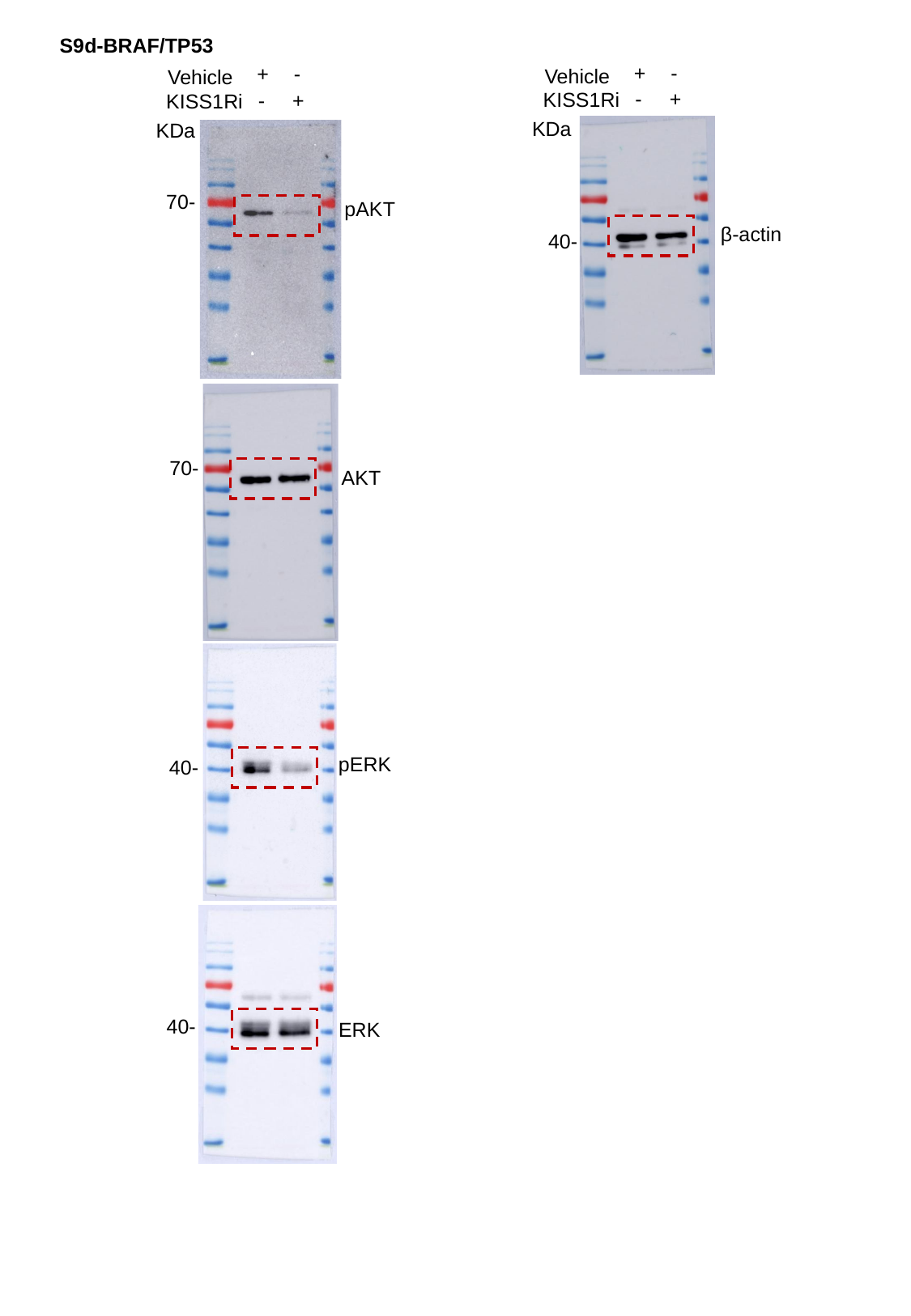

S9d-BRAF/TP53
+
-
Vehicle
-
+
KISS1Ri
+
-
Vehicle
-
+
KISS1Ri
KDa
KDa
70-
pAKT
β-actin
40-
70-
AKT
pERK
40-
40-
ERK

## Slide 12
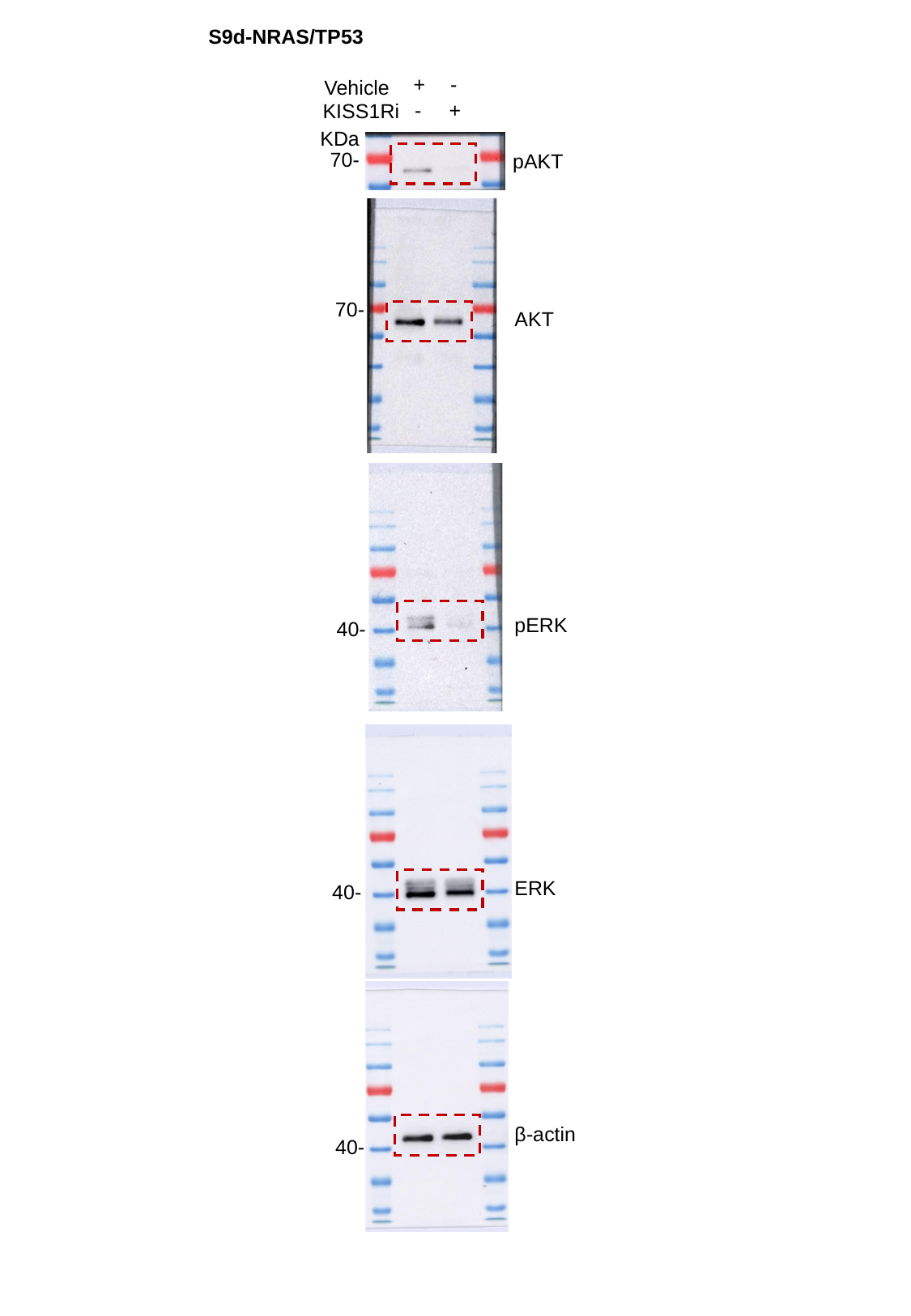

S9d-NRAS/TP53
+
-
Vehicle
-
+
KISS1Ri
KDa
70-
pAKT
70-
AKT
pERK
40-
ERK
40-
β-actin
40-
